# Analysis of tomato bacterial wilt transmission dynamics with behavioral responses: A mathematical and optimal control approach

**DOI:** 10.1016/j.parepi.2026.e00548

**Published:** 2026-09-29

**Authors:** Md. Asraful Islam, Mymuna Akther

**Affiliations:** Department of Mathematics, Jagannath University, Dhaka 1100, Bangladesh

**Keywords:** Tomato bacterial wilt disease (TBWD), Basic reproduction number, PRCC global sensitivity, Backward bifurcation Behavioral responses, Optimal control

## Abstract

In this mathematical model-based study, the transmission mechanism of bacterial wilt in tomatoes is investigated. It determines the fundamental reproduction number, assesses the equilibrium between disease-free and endemic conditions, and evaluates stability on global and local scales. According to sensitivity and global sensitivity analyses, which identify the factors with the greatest effect on the number of reproductions, infection, and transition rates, these are the most important factors to control. Studies of bifurcation have shown that backward bifurcation conditions exist. This proves that the dynamics of disease persistence are much more complicated than we initially thought. The model shows that early intervention and management of soil bacteria can help stop the development of disease. Parameters like as cost, efficacy, and transmission influence the adoption of control measures for tomato wilt, according to behavioral responses dynamics are discussed. Several iterations of robustness and stability tests have been conducted using Lyapunov exponents and the basin of attraction. Through targeted environmental treatments and biological interventions, our results identify potential methods to reduce tomato bacterial wilt.

## Highlights

• Developed a stage-structured model incorporating soil bacteria and behavioral responses in tomato bacterial wilt.
• Identified backward bifurcation, indicating disease persistence even when the reproduction number is below unity.
• Sensitivity and global analyses identify transmission rate as key, while removal and mortality reduce disease spread.
• Optimal control strategies highlight early intervention and soil management as effective measures for disease mitigation.

## 1 Introduction

Tomatoes are the most popular vegetable farmed in the world. These foods are rich in vitamins A, C, and K, as well as antioxidants like lycopene, which support the immune system. Tomatoes are a common food in many parts of the world, and they also help ensure people can readily access and eat them healthily. Farmers can make a lot of money growing tomatoes because there is strong demand for them in both fresh markets and processing firms. When grown well, tomatoes support rural economies, promote eco-friendly farming practices, and increase smallholder farmers' incomes. A mathematical model is developed to describe the dynamics of tomato bacterial wilt disease transmission in Mukono, Uganda. The Markov Chain Monte Carlo method is used to do both parameter estimation and stability analysis. Evidence indicates that crop rotation and soil treatment, integral components of integrated management systems, can significantly reduce the likelihood of disease dissemination (Remo et al., 2018). Both early and late blight still cause significant yield losses, underscoring the importance of effective diagnostic and evaluation methods (Samui et al., 2020). Because training neural networks to learn is hard, there aren't usually enough annotated datasets to consistently find tomato illnesses. A recent study aimed to enhance the accuracy of sickness detection by a data augmentation technique centered on the development of competitive designs (Deng et al., 2021).

A mathematical model was also developed to show how infectious diseases spread in farming, taking into account both direct and indirect ways germs might move. Even while deep learning models are quite good at classifying things, they are not very useful in real life because they need a lot of processing power. To solve this problem, a simple concept called Light Mixer was suggested (Cantó et al., 2021). A classification model that used a residual network architecture and bio-inspired optimization to achieve 97.3% accuracy demonstrated once again the usefulness of architectural tweaking (Ezhilarasan et al., 2024). Deep learning techniques are becoming increasingly useful for detecting diseases because they can extract features directly from image data without additional processing. Using pre-trained convolution models to distinguish healthy from sick tomato leaves works very well (Khasawneh et al., 2022; Saeed et al., 2023). This model achieves a very high level of accuracy in object classification (Trivedi et al., 2021; Cen et al., 2022). It employs hyper spectral photography and an improved support vector machine trained using genetic methods. The persistence of bacterial wilt prompted researchers in southern China to develop this model.

Other variants have also done well, and it's all because they are light and work well. A segmentation model using a Transformer architecture achieved 0.98 precision and an inference speed of 37 frames per second (Liu et al., 2023), whereas a convolution architecture reached 96% accuracy in preliminary detection of tomato leaf diseases (Najim et al., 2024**).** A convolution-based classifier achieved 99.50% accuracy when evaluated on several samples (Bayram et al., 2022; Xie et al., 2022). Another model achieved 94.44% accuracy and an F1-score of 0.9475 when presented with images with complex backgrounds and modest differences across classes. A deep learning model that combined classification with transfer learning achieved 99.62% accuracy (Mahakud et al., 2022), despite sparse, imbalanced data. The main factors used in mathematical models to forecast tomato growth in greenhouses were plant height and dry matter (Peng et al., 2023; Fang et al., 2022). A simpler eight-layer convolution network achieved 98.4% accuracy, outperforming both traditional AI and trained models (Agarwal et al., 2020). A modular robotic system was developed to detect and identify sick tomato plants in real time, enabling easier inspection (Ouyang et al., 2022). Finally, multi-scale feature fusion and attention-based procedures have made categorization even more accurate and stable (Wang et al., 2024). Using a dual-path attention model with cascaded convolution (Liang and Jiang, 2023), disease detection on tomato leaves improved significantly, and crop failure decreased at the same time.

To enhance the precision of diseased-region detection, a novel deep learning model for tomato disease segmentation was developed, comparing the infected leaf area with that of healthy leaves. Using uncontrolled pre-training and spatially regulated co-attention approaches made training far more efficient and faster (Wu et al., 2022). It was able to significantly reduce disease severity and increase tomato yields by combining the biocontrol bacterium *Bacillus subtilis* with macronutrients (nitrogen, phosphate, and potassium) and the micronutrient zinc (Awan et al., 2022). Another mathematical model examined the co-dynamics of the corona virus and human immunodeficiency virus under therapeutic and preventive strategies, indicating that integrated treatments can significantly reduce transmission (Kotola et al., 2023). Discrete susceptible-infected-recovered (SIR) models exhibited chaotic behavior, as evidenced by intricate transitions, including Neimark-Sacker bifurcations (Li et al., 2022).

A seven-compartment gender-specific coronavirus model exhibited stability via forward bifurcation, also indicating sensitivity to contact rates (Tsetimi et al., 2022). Modeling the kinetics of the human immunodeficiency virus (Srivastava and Umrao, 2023) showed that viral spread from one cell to another and immune suppression without killing cells both contribute to worsening the disease. Models depicting the interplay between tumors and the immune system, along with numerous immune cells, have elucidated therapeutic optimization and bifurcation-induced transitions (Makhlouf et al., 2020). Coupled map lattice methods were developed to investigate the development of patterns and bifurcation structures in the Gierer-Meinhardt reaction-diffusion system (Liu et al., 2022). In contrast, Lie symmetry analysis and chaos theory were applied to analyze symmetry-breaking phenomena in physical systems. A general epidemiological model for polycyclic plant pathogen outbreaks was employed to analyze the impact of practical control strategies on disease transmission (Kazmi et al., 2023; González-Domínguez et al., 2020). A convolution neural network with support vector machines, it obtains a model that can detect plant illnesses with more than 99% accuracy **(**Prince et al., 2024**)**. The accuracy of recognizing maize diseases exceeded 98% when using deep models such as CD-MobileNetV3 (Bi et al., 2023). These models were improved by using hybrid datasets and better loss functions. Several other hybrid methodologies have demonstrated successful crop classification by integrating optimization algorithms and machine learning frameworks (Yağ and Altan, 2022; Gu et al., 2020).

It was used Lyapunov stability analysis to look at pathogen-host immunological models (Liu et al., 2022), and the BBCPF framework helped us gather disease-related entities for knowledge management **(**Zhang et al., 2022**).** Recently, researchers used fractional-order epidemic models to understand better how the Ebola and COVID-19 viruses spread. These models demonstrated intricate stability tendencies **(**Nisar et al., 2024). Utilizing few-shot learning techniques and super-resolution networks significantly enhanced disease identification in intricate imaging scenarios (Fudolig and Howard, 2020).

Tomato bacterial wilt disease (TBWD) is caused by *Ralstonia solanacearum*. It remains a serious problem for tomato crops worldwide, especially in tropical and subtropical regions. It is a tough problem for farmers since it stays in the soil, can affect many different types of plants, and is hard to control with standard approaches. The pathogen can remain in the soil and re-infect crops, which is why chemical treatments and crop rotation don't work particularly well over time.

One of the primary motivations behind this research is the requirement for an improved mathematical model that integrates academic epidemiology with farm management in the real world. The research intends to expand the grasp of the mechanisms facilitating disease propagation by adding plant growth phases, soil-bacteria interactions, and complex analytical approaches such as sensitivity analysis, bifurcation analysis, and Lyapunov stability.

A major flaw in the present research is the lack of mathematically sound models that simultaneously integrate soil bacterial reservoirs, stage-structured plant dynamics, and nonlinear phenomena, such as backward bifurcation, in tomato bacterial wilt disease (TBWD). Many modeling methods still use overly simple SEIR frameworks that don't account for essential biological realities, such as how soil can indirectly spread disease or how tomato plants grow at different stages. Complicated nonlinear dynamics, which are typically not accounted for in studies, also affect control thresholds and disease persistence. Also, advanced analytical techniques such as sensitivity analysis, bifurcation analysis, and Lyapunov stability are often not given enough credit, and there is a lack of integration of control strategies that work best at several intervention points. Because of these problems, current models aren't as helpful as they could be in developing effective ways to address illness.

A novel, realistic model that incorporates soil bacterial dynamics, advanced nonlinear analysis, and multiple stages of plant growth is the most important contribution this study makes. The purpose of this work is to improve the representation of tuberculosis with diarrhea (TBWD) transmission by integrating environmental bacterial feedback and stage-structured compartments. This study departs from usual methodologies. Backward bifurcation is an important advance because it disproves traditional control assumptions by showing that sickness persistence can occur even when the basic reproduction number is less than one. This is a big advancement. In this paper, a multi-intervention optimum control framework is presented. This framework incorporates stability studies based on Lyapunov's theory and sensitivity analyses. It offers both theoretical and practical insights for the effective treatment of illness.

The introduction, the first part of the manuscript, outlines the main goals, the lack of research, and background information. Next, we have discussed how the model was built. After that, we have undertaken a mathematical analysis that examines stability, bifurcations, equilibriums, and $R_{0}$. The next step is to check the Lyapunov exponent, run numerical simulations, perform sensitivity analysis, and behavioral responses. Once the statistics have been discussed, the most effective control methods are looked at, and decisions are made.

## 2 Formulation of the model

The first system (2.1) makes a mathematical model of TBWD, which stands for tomato bacterial wilt disease. This model follows the life cycle of diseased tomato plants as they interact with soil bacteria. $Y_{T}$, and $\delta Y_{T}$ show the rates at which young, vulnerable tomatoes are recruited, infected, and naturally killed off. The second equation shows the number of infected tomatoes. The number of infected tomatoes $\left(I_{T}\right)$ increases as infection spreads from immature to mature tomatoes. However, the number of infected tomatoes $\left(I_{T}\right)$ declines due to growth, maturity, or natural selection. The rate at which tomatoes reach the transitional stage after being infected is called$\;T_{T}$. Tomatoes exit the transitional phase either through removal or upon reaching maturity, at a rate denoted as $\eta T_{T}$. In the fourth equation, a fraction $\left(1-\rho \right)$ that stands for the immature tomatoes is moved straight to the diseased class$\left(D_{T}\right)$. As they get older or die, they get smaller. In the fifth equation, the mature tomatoes $\left(M_{T}\right)$ gain additional members and lose some due to natural causes during the infected and unwell stages. Lastly, the bacterial population in soil B increases because both healthy and ill plants are present. The population then declines at a natural rate of decay, represented by the symbolω. With this method, we can follow the steps that lead to infection as tomatoes grow and see how soil bacteria affect and are affected by those steps.

(2.1) $$\left\{\begin{array}{c} \frac{dY_{T}}{\mathrm{dt}}=\Lambda -\rho Y_{T}I_{T}-\delta Y_{T}\\ \frac{dI_{T}}{\mathrm{dt}}=\rho Y_{T}I_{T}-\left(\mu +\tau +\delta \right)I_{T}\\ \frac{dT_{T}}{\mathrm{dt}}=\tau I_{T}-\eta T_{T}\\ \frac{dD_{T}}{\mathrm{dt}}=\left(1-\rho \right)Y_{T}-\left(\mu +q\right)D_{T}\\ \frac{dM_{T}}{\mathrm{dt}}=\xi I_{T}+qD_{T}-\mu M_{T}\\ \frac{\mathrm{dB}}{\mathrm{dt}}=\delta I_{T}-\xi I_{T}+\mu M_{T}-\mathrm{\omega B} \end{array}\right)$$

With initial conditions,

(2.2) $$Y_{T}\geq 0,I_{T}\geq 0,T_{T}\geq 0,D_{T}\geq 0,M_{T}\geq 0,B\geq 0$$

From the Fig. 1 and Fig. 2, the deterministic compartmental model was chosen due to the persistence of causative infection in the environment and the development of the disease during several periods of plant growth. Therefore, the tomato population is denoted as $Y_{T}$= Young (Susceptible) tomatoes, $I_{T}=$ Infected tomatoes, $T_{T}$= Transitioning tomatoes, $D_{T=}$ Diseased tomatoes, $M_{T}$= Mature tomatoes, B = Bacterial population in soil.

**Fig. 1:**
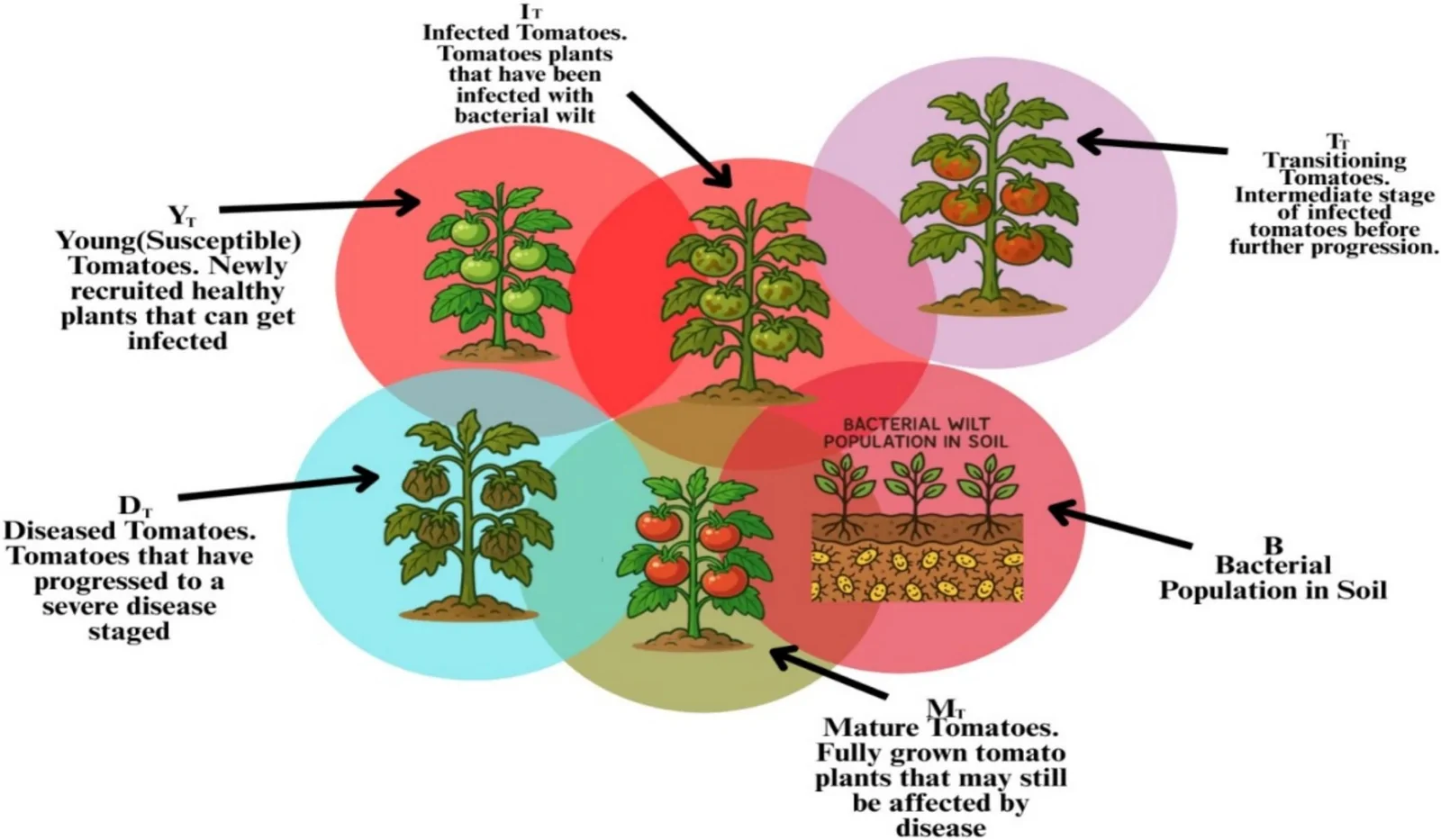
The tomato bacterial wilt model is a compartmental model that demonstrates how plants go from being young (Y) to infected (I), then to transitional (T), sick (D), mature (M), and finally to soil bacteria (B).

**Fig. 2:**
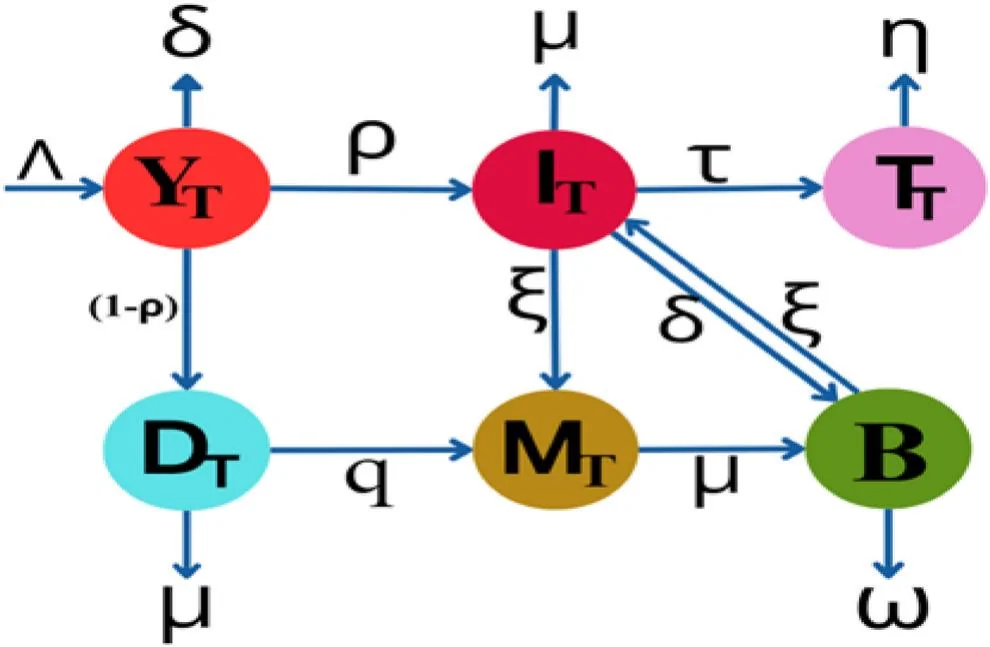
The biological process of tomato bacterial wilt disease transmission as depicted in a compartmental diagram.

This system enables us to analyze simultaneously the emergence, elimination, maturation of diseases, and persistence of bacteria in the environment, as well as the progression of plant stages, infection dynamics, and disease development. The recruitment of susceptible plants occurs at a constant rate, the infection transmission technique is an effective mass-action approach, and the average rates of transition between compartments are constant. Infection or mature plant processes increase the soil bacterial population, and then bacterial breakdown causes a natural decrease. With these assumptions, we may study intervention possibilities in a mathematically tractable and medically interpretable framework, study stability and bifurcation, investigate the disease-free and endemic equilibria, and compute $R_{0}$. The model is assumed to remain constant over the study period with respect to effective transmission and model parameters. The dominant mechanism of infection in this model is the transmission term to plants, whereas the ambient bacterial compartment represents pathogen clearance and persistence. This can pave the way for future improvements to the model that take into account factors such as spatial heterogeneity, seasonal variation, stochastic environmental impacts, and explicit transmission from soil to plants.

The values of Λ,ρ,δ,τ,η, and q are chosen as baseline parameters from the numerical study of Remo et al. (2018). There were no experimental estimates that directly matched the effect of infected tomatoes on bacterial dynamics in the model and on natural bacterial degradation in the soil; thus, suitable baseline values for ξ and ω were chosen. Rather than treating these assumed values as fixed constants, we use them in exploratory numerical analysis to examine the impact of varying parameters or conducting alternative sensitivity analyses on the model's dynamics.

## 3 Analysis of the model

The theory that assures positive initial values for the system reactions (2.1) is where everything begins.

(3.1) $$\Omega =\left\{\left(Y_{T},I_{T},T_{T},D_{T},M_{T},B\right)\in R_{+}^{6}:0\leq Y_{T}+I_{T}+T_{T}+D_{T}+M_{T}+B\leq \frac{\Lambda }{\delta }\right\}$$

### 3.1 Positivity results

One of the main purposes of this research is to find out whether, at all times *t*, none of the system's operational parameters (2.1) are negative. $Y_{T}\left(0\right),I_{T}\left(0\right),T_{T}\left(0\right),D_{T}\left(0\right)$,$M_{T}\left(0\right),$and$\;B\left(0\right)$ are the initial values used in this study.

Theorem 1 For everyt>0, if all the solutions ($Y_{T},I_{T},T_{T},D_{T}$,$M_{T},B)$ of the system (2.1) with the initial values (2.2) fulfill $Y_{T}>0,I_{T}>0,T_{T}>0,D_{T}>0,M_{T}>0,B>0$ for all t>0, then system (2.1) is positively invariant & attracts inside$\;R_{+}^{6}$.

Proof: This behavior could be caused by the first Eq. (2.1):

$$\frac{dY_{T}}{\mathrm{dt}}=\Lambda -\rho Y_{T}I_{T}-\delta Y_{T}$$

$$=\Lambda -\left(\rho I_{T}+\delta \right)Y_{T}$$

$$=\Lambda -\psi \left(t\right)Y_{T}$$

Where $\psi \left(t\right)=\left(\rho I_{T}+\delta \right)$

Once it's finished, we can integrate to get the formula from (Samui et al., 2020).

$$Y_{T}=Y_{0}\exp\left(-\int_{0}^{t}\psi \left(t\right)dY_{T}\right)+\lambda_{Y_{T}}\exp\left(-\int_{0}^{t}\psi \left(t\right)dY_{T}\right)\;\left(-\int_{0}^{t}\psi \left(u\right)\mathrm{du}\right)>0$$

This shows that $Y_{T}$ is always non-negative. For every value of *t*, $Y_{T},I_{T},T_{T},D_{T},M_{T}$, and *B* are all greater than or equal to 0. After the simulations that came before this one are done. The study shows that all solution pathways of system (2.1) have a positive value for all t>0.

### 3.2 Boundedness

Theorem 2 The starting point of data is given by$\;Y_{T}\left(0\right)\geq 0,I_{T}\left(0\right)\geq 0,T_{T}\left(0\right)\geq 0,D_{T}\left(0\right)\geq 0,M_{T}\left(0\right)\geq 0,B(0\geq 0\in \Omega$. The set of solutions to the system Eq. (2.1), which includes$\;Y_{T},I_{T},T_{T},D_{T},M_{T},B$, is non-negative and bounded (Samui et al., 2020) for all t≥0.

**Proof:**

Simplifying the systems (2.1), $\frac{\mathrm{dN}\left(t\right)}{\mathrm{dt}}=\Lambda -\mu \left(Y_{t}+I_{t}+T_{t}+D_{t}+M_{t}\right)-\delta D_{t}-\mathrm{\omega B}$

Since $D_{t}\geq 0$ and $B\left(t\right)\geq 0$ for all t≥0,we have $\frac{\mathrm{dN}\left(t\right)}{\mathrm{dt}}\leq \Lambda -\mu \left(Y_{t}+I_{t}+T_{t}+D_{t}+M_{t}\right)-\mathrm{\omega B}$

$$\kappa =\min\left\{\mu ,\omega \right\}>0.$$

(3.2) $$\frac{\mathrm{dN}\left(t\right)}{\mathrm{dt}}=\Lambda -\mathrm{\kappa N}\left(t\right)$$

Consider the auxiliary linear differential equation

(3.3) $$\frac{\mathrm{dZ}\left(t\right)}{\mathrm{dt}}=\Lambda -\mathrm{\kappa Z}\left(t\right),Z\left(0\right)=N\left(0\right)$$

The solution of (3.3) is $Z\left(t\right)=\frac{\Lambda }{\kappa }-\left(N\left(0\right)-\frac{\Lambda }{\kappa }\right)$ $e^{\mathrm{kt}}$

By the comparison principle for ordinary differential inequality (3.2) implies$\;N\left(t\right)\leq Z\left(t\right)$, ∀t≥0.

Taking the limit superior as t→∞

$$\lim_{t\to \infty }\mathrm{Sup}\;N\left(t\right)\leq \frac{\Lambda }{\kappa }$$

Since each state variables is non-negative and bounded by $N\left(t\right)$, we conclude that $0\leq Y_{t,}I_{t},T_{t},D_{t},M_{t},B\leq \frac{\Lambda }{\kappa },\forall t\geq 0.$

Thus, all solutions of system (2.1) are uniformly bounded in Ω.

This is the end of the proof for Theorem 2.

The theory outlined above posits that every solution related to the model is both nonnegative and constrained. In other words, the model behaves in line with biological principles.

### 3.3 Tomato disease free equilibrium point (DFE)

Each of the compartments' time derivatives was set to zero to discover the system's DFE point. This is in line with the scenario where the population stays the same throughout time. It is time to set:

$$\frac{dY_{T}}{\mathrm{dt}}=\frac{dI_{T}}{\mathrm{dt}}=\frac{dT_{T}}{\mathrm{dt}}=\frac{dD_{T}}{\mathrm{dt}}=\frac{dM_{T}}{\mathrm{dt}}=\frac{\mathrm{dB}}{\mathrm{dt}}=0$$

Solving first equation of the model (2.1):$\frac{dY_{T}}{\mathrm{dt}}=0⟹\Lambda -\rho Y_{T}I_{T}-\delta Y_{T}=0$

Since we are seeking the disease-free scenario (i.e., persons without infection), we can set $I_{T}=0$. So,

$$\Lambda -\rho Y_{T}I_{T}-\delta Y_{T}=0⟹Y_{T}=\frac{\Lambda }{\delta }$$

The only non-zero value is $Y_{T}=\frac{\Lambda }{\delta }$, which represents the number of individuals in the susceptibles compartment. Hence the Disease Free Equilibrium point is:$\;E_{0}=\left(Y_{T},I_{T},T_{T},D_{T},M_{T},B\right)=\left(\frac{\Lambda }{\delta },00000\right)$

### 3.4 Existence of endemic equilibrium point (EE)

Let, E = ($Y_{T}^{*},I_{T}^{*},T_{T}^{*},D_{T}^{*},M_{T,}^{*}B)$ be the endemic equilibrium point. For this point put $Y_{T}^{*}\neq 0,$

$E_{T}^{*}\neq 0,I_{T}^{*}\neq 0,T_{T}^{*}\neq 0,R_{T}^{*}\neq 0$, B≠0.

Solving the Eq. (2.1):$\;Y_{T}=\frac{\Lambda }{\left(\rho I_{T}+\delta \right)}$, $I_{T}=\frac{\rho \Lambda -\left(\mu +\tau +\delta \right)\delta }{\left(\mu +\tau +\delta \right)\rho }$, $T_{T}=\frac{\tau I_{T}^{*}}{\eta }$, $D_{T}=\frac{\left(1-\rho \right)\Lambda }{\left(\mu +q\right)\left(\rho I_{T}+\delta \right)}$, $M_{T}=\frac{\xi I_{T}^{*}+qD_{T}^{*}}{\mu }$, $B=\frac{\left(\delta -\xi \right)I_{T}^{*}+{\mathrm{\mu M}}_{T}^{*}}{\omega }$

The Endemic equilibrium point is

$$E=\left(Y_{T}^{*},I_{T}^{*},T_{T}^{*},D_{T}^{*},M_{T,}^{*}B\right)=\left(\frac{\Lambda }{\left(\rho I_{T}+\delta \right)},\frac{\rho \Lambda -\left(\mu +\tau +\delta \right)\delta }{\left(\mu +\tau +\delta \right)\rho },\frac{\tau I_{T}^{*}}{\eta },\frac{\left(1-\rho \right)\Lambda }{\left(\mu +q\right)\left(\rho I_{T}+\delta \right)},\frac{\xi I_{T}^{*}+qD_{T}^{*}}{\mu },\frac{\left(\delta -\xi \right)I_{T}^{*}+{\mathrm{\mu M}}_{T}^{*}}{\omega }\;\right).$$

### 3.5 Basic reproduction number ($R_{o})$

The basic reproduction number$\left(R_{o}\right)$ is an important threshold factor when it comes to traditional epidemiological approaches for predicting how illnesses will spread. This method lets us figure out how many additional illnesses a sick person usually generates in a community that is particularly likely to get sick while they are out sick. $R_{o}$ is an important figure to consider when forecasting the likelihood that a community can eliminate or greatly slow the spread of a contagious disease. We use the generation matrix (Samui et al., 2020) below to show the basic reproduction number for system (2.1).

(3.4) $$\left\{\begin{array}{c} \frac{dI_{T}}{\mathrm{dt}}=\rho Y_{T}I_{T}-\left(\mu +\tau +\delta \right)I_{T}\\ \frac{dM_{T}}{\mathrm{dt}}==\xi I_{T}+qD_{T}-\mu M_{T})\\ \frac{\mathrm{dB}}{\mathrm{dt}}=\delta I_{T}-\xi I_{T}+\mu M_{T}-\mathrm{\omega B} \end{array}\right)$$

Then infection matrix, F=$\left[\begin{array}{ccc} \rho Y_{T} & 0 & 0\\ 0 & 0 & 0\\ 0 & 0 & 0 \end{array}\right]\;$and V=$\left[\begin{array}{c} -\left(\mu +\tau +\delta \right)I_{T}\\ \xi I_{T}+qD_{T}-\mu M_{T}\\ \delta I_{T}-\xi I_{T}+\mu M_{T}-\mathrm{\omega B} \end{array}\right]$

The elimination of diseased cells as a result of development is shown by the transition matrix V. Thus the transition matrix is, V=$\left[\begin{array}{ccc} -\left(\mu +\tau +\delta \right) & 0 & 0\\ \xi & -\mu & 0\\ \delta -\xi & \mu & -\omega \end{array}\right]$

$$∴V^{-1}=\frac{1}{\omega \mu \left(\mu +\tau +\delta \right)}\left[\begin{array}{ccc} \omega \mu & 0 & 0\\ \omega \;\xi & \omega \;\left(\mu +\tau +\delta \right) & 0\\ \mu \delta & -\mu \left(\mu +\tau +\delta \right) & \mu \left(\mu +\tau +\delta \right) \end{array}\right]$$

$$=\left[\begin{array}{ccc} \frac{1}{\left(\mu +\tau +\delta \right)} & 0 & 0\\ \frac{\;\xi }{\mu \left(\mu +\tau +\delta \right)} & \frac{1}{\mu } & 0\\ \frac{\delta }{\omega \left(\mu +\tau +\delta \right)} & \frac{1}{\omega } & \frac{1}{\omega } \end{array}\right]$$

$$∴FV^{-1}=\left[\begin{array}{ccc} \rho Y_{T} & 0 & 0\\ 0 & 0 & 0\\ 0 & 0 & 0 \end{array}\right]\left[\begin{array}{ccc} \frac{1}{\left(\mu +\tau +\delta \right)} & 0 & 0\\ \frac{\;\xi }{\mu \left(\mu +\tau +\delta \right)} & \frac{1}{\mu } & 0\\ \frac{\delta }{\omega \left(\mu +\tau +\delta \right)} & \frac{1}{\omega } & \frac{1}{\omega } \end{array}\right]$$

$$=\left[\begin{array}{ccc} \frac{\rho Y_{T}}{\left(\mu +\tau +\delta \right)} & 0 & 0\\ 0 & 0 & 0\\ 0 & 0 & 0 \end{array}\right]$$

$$∴\left|FV^{-1}-\mathrm{\lambda I}\right|=\left|\begin{array}{ccc} \frac{\rho Y_{T}}{\left(\mu +\tau +\delta \right)}-\lambda & 0 & 0\\ 0 & -\lambda & 0\\ 0 & 0 & -\lambda \end{array}\right|=0$$

$\lambda_{1}=\left|\frac{\rho Y_{T}}{\left(\mu +\tau +\delta \right)}\right|=\frac{\rho Y_{T}}{\left(\mu +\tau +\delta \right)}=R_{0}$ is the largest eigen value.

$$\lambda_{2}=\lambda_{3}=0$$

$R_{0}=\frac{\rho Y_{T}}{\left(\mu +\tau +\delta \right)}$, Suppose$\;Y_{T}=1$, then the basic reproduction number is,

(3.5) $$R_{0}=\frac{\rho }{\left(\mu +\tau +\delta \right)}$$

The basic reproduction number $R_{0}$ is strongly affected by the balance between the transmission rate ρ and the transition rate τ, as seen in Fig. 3. An increase inρ is also an increase in $R_{0}$, so a stronger likelihood for tomato bacterial wilt to infiltrate and remain in the crop population. Conversely, increasing τ results in a fall in $R_{0}$ because infected plants go through the stages more rapidly, lowering the time available for transmission. Fig. 4 also shows that increasing ρ leads to an increase in $R_{0}$ in the present model, while decreasing the disease-induced mortality rate δ leads to a fall in $R_{0}$. From an epidemiological point of view, it represents the removal of disease-carrying plants from the transmission channel. These results suggest that the major objectives of disease control techniques should be to restrict effective transmission and the duration of time during which diseased plants are transmitting disease. Therefore, by early detection and removal of contaminated plants, proper sanitation and other procedures that restrict effective transmission, continued spread of disease can be minimized by reducing $R_{0}$ to below unity.

**Fig. 3:**
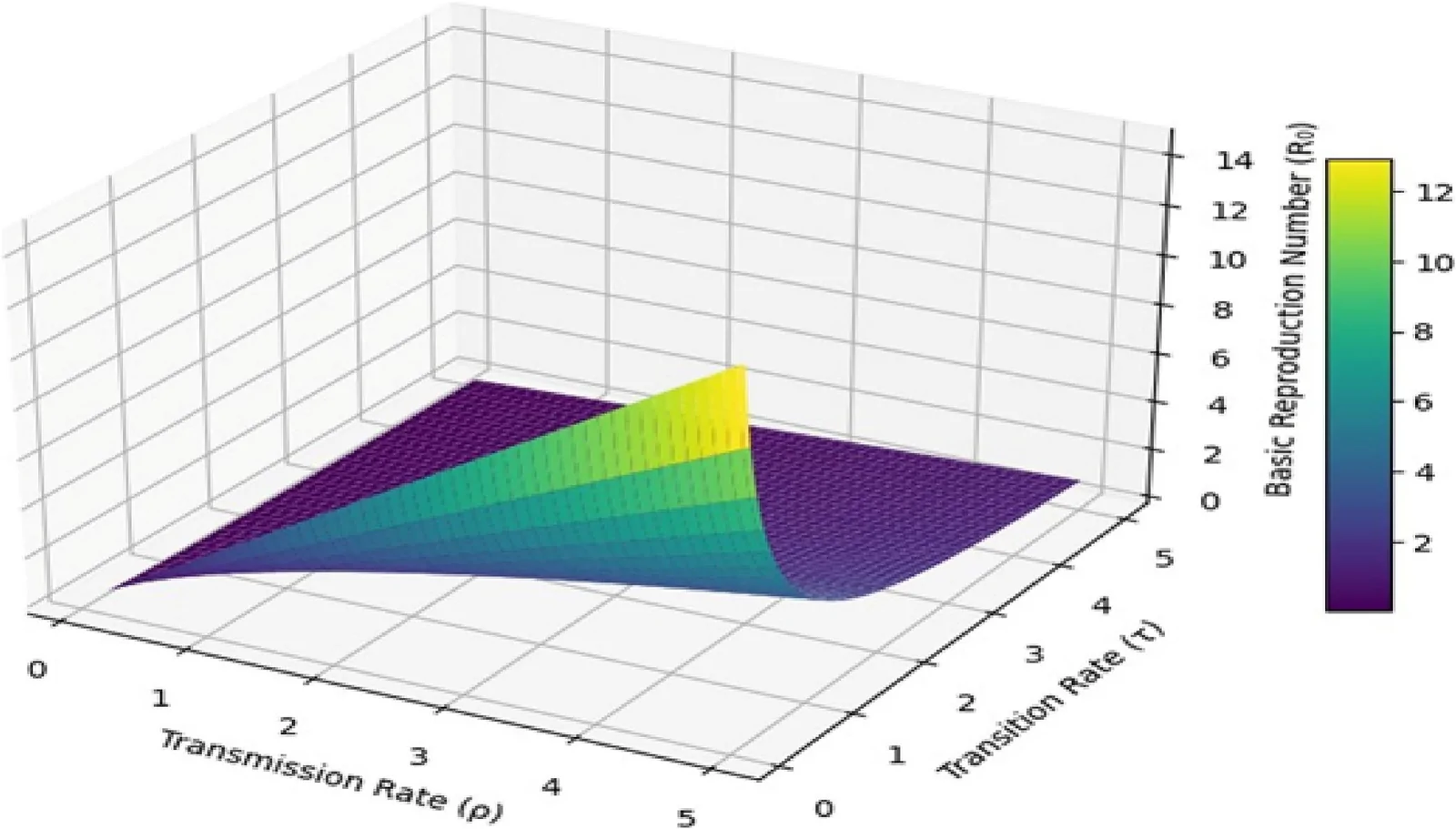
3D surface plot showing the basic reproduction number $\left(R_{0}\right)$ in connection to the transmission rate $\left(\rho \right)$and the transition rate $\left(\tau \right)\;$using the Eq. (3.5).

**Fig. 4:**
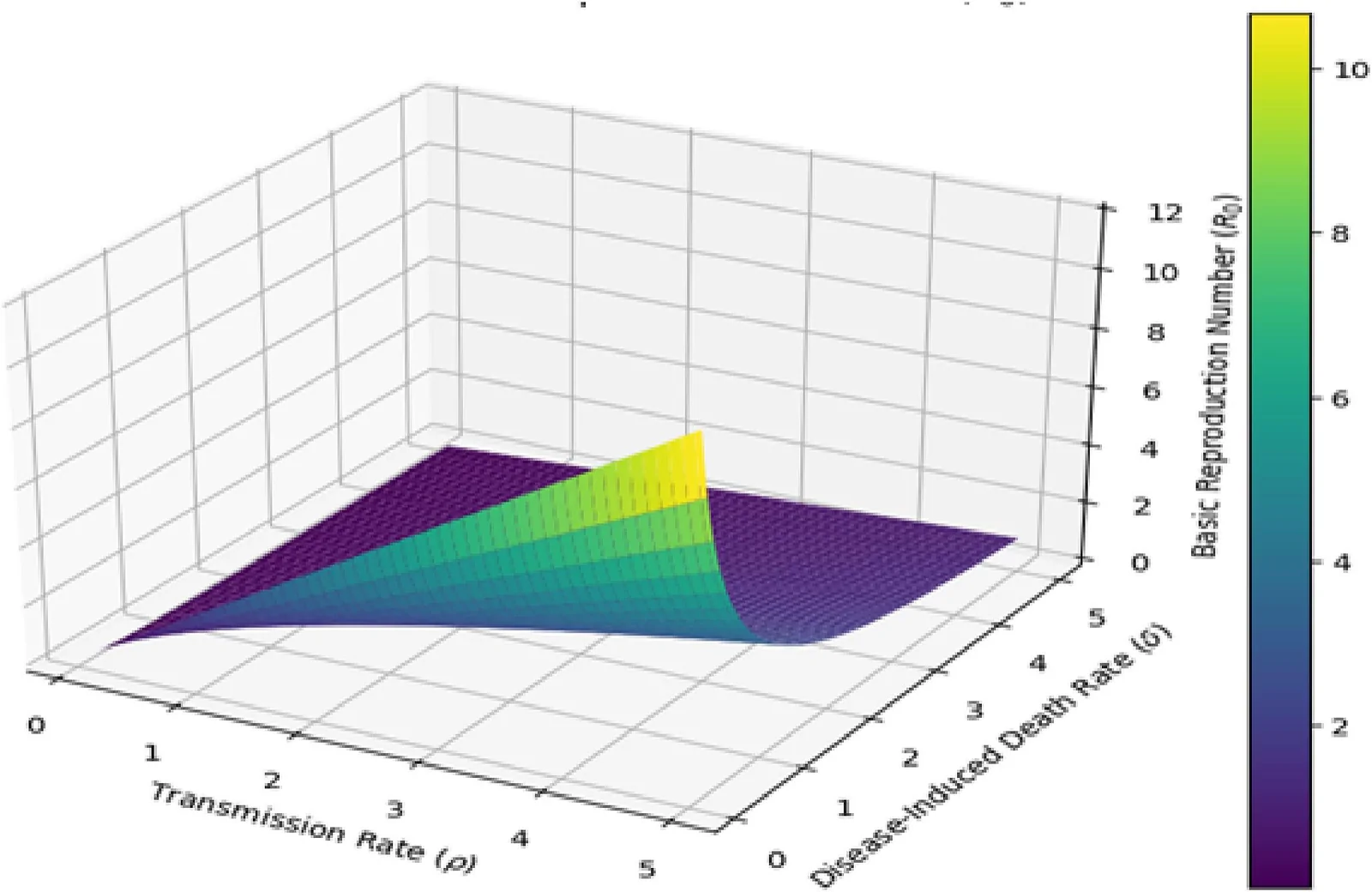
3D surface plot showing the basic reproduction number $\left(R_{0}\right)$ in connection to the transmission rate $\left(\rho \right)$and the disease-induced death rate (δ) using the Eq. (3.5).

### 3.6 Analysis of stability

The stability of the disease-free balance can be measured by eliminating the system (2.1) and then getting the Jacobian matrix:

(3.6) $$J=\left[\begin{array}{cccccc} -\left(\rho I_{T}+\delta \right) & -\left(\rho I_{T}\right) & 0 & 0 & 0 & 0\\ \rho I_{T} & \rho Y_{T}-\left(\mu +\tau +\delta \right) & 0 & 0 & 0 & 0\\ 0 & \tau & -\eta \; & 0 & 0 & 0\\ \left(1-\rho \right) & 0 & 0 & -\left(\mu +q\right) & 0 & 0\\ 0 & \xi & 0 & q & -\mu & 0\\ 0 & \delta -\xi & 0 & 0 & \mu & -\omega \end{array}\right]$$

### 3.7 Analysis of local stability

Theorem 3 The disease free equilibrium point $E_{0}=\left(\frac{\Lambda }{\delta },00000\right)$ exists and it is locally asymptotically stable for $R_{0}<1$ otherwise unstable.

**Proof:** To determine the local stability of $E_{0}=\left(\frac{\Lambda }{\delta },00000\right),$we calculates the Jacobian Matrix using the Eq. (7) around the DFE, $E_{0}$ is given by:

$$∴J\left(E_{0}\right)=\left[\begin{array}{cccccc} -\delta & 0 & 0 & 0 & 0 & 0\\ 0 & \rho \frac{\Lambda }{\delta }-\left(\mu +\tau +\delta \right) & 0 & 0 & 0 & 0\\ 0 & \tau & -\eta & 0 & 0 & 0\\ \left(1-\rho \right) & 0 & 0 & -\left(\mu +q\right) & 0 & 0\\ 0 & \xi & 0 & q & -\mu & 0\\ 0 & \delta -\xi & 0 & 0 & \mu & -\omega \end{array}\right]$$

The characteristic equation of $J\left(E_{0}\right)$ corresponding to the eigenvalues is

$$\left|J\left(E_{0}\right)-\lambda I_{6}\right|=0$$

The eigenvalues of *J(*$E_{0})$*are:*$\;\lambda_{1}=-\delta <0$*,*$\;\lambda_{2}=\rho \frac{\Lambda }{\delta }-\left(\mu +\tau +\delta \right)<0$*,*

$$∴\rho <\frac{\left(\mu +\tau +\delta \right)\delta }{\Lambda }$$

$\lambda_{3}=-\eta <0$, $\lambda_{4}=-\left(\mu +q\right)<0$, $\lambda_{5}=-\mu <0,\lambda_{6}=-\omega <0$.

Here all parameters are positive and $\rho <\frac{\left(\mu +\tau +\delta \right)\delta }{\Lambda }$.

It has observed that $\lambda_{1},\lambda_{2},\lambda_{3},\lambda_{4},\lambda_{5},\lambda_{6}<0$ if $R_{0}<1\left(i.e.\rho <\frac{\left(\mu +\tau +\delta \right)\delta }{\Lambda }\right)$ and $\lambda_{1},\lambda_{2},\lambda_{3},\lambda_{4},\lambda_{5},\lambda_{6}>0$ if $R_{0}>1\left(i.e.\rho >\frac{\left(\mu +\tau +\delta \right)\delta }{\Lambda }\right)$. Hence the disease free equilibrium point (DFE) is locally asymptotically stable if $R_{0}<1$ and it is unstable if $R_{0}>1$.

### 3.8 Global stability of the tomato disease free state

In this part, we talk about stable equilibrium points on a global scale. Using the La Salle invariance principle and a suitable Lyapunov function, we show that the DFE point is globally stable. The theorem below shows that both equilibrium points must be stable at every point.

Theorem 4 The DFE point ($E_{0}$) is globally asymptotically stable (GAS) if $R_{0}\leq 1.$

**Proof:** We look into a Lyapunov function (Samui et al., 2020),

(3.7) $$L_{1}=I_{T}-I_{T}^{0}-I_{T}^{0}\ln\frac{I_{T}}{I_{T}^{0}}+M_{T}+B$$

As we apply system (2.1) to our Lyapunov function (3.7), we get the time derivative as

$$\frac{dL_{1}}{\mathrm{dt}}=\frac{dI_{T}}{\mathrm{dt}}-I_{T}^{0}-\frac{d}{\mathrm{dt}}\left[\ln\left(\frac{I_{T}}{I_{T}^{0}}\right)\right]+\frac{dM_{T}}{\mathrm{dt}}+\frac{\mathrm{dB}}{\mathrm{dt}}=\frac{dI_{T}}{\mathrm{dt}}-I_{T}^{0}.\frac{1}{I_{T}}\frac{dI_{T}}{\mathrm{dt}}+\frac{dM_{T}}{\mathrm{dt}}+\frac{\mathrm{dB}}{\mathrm{dt}}$$

$$=\left(1-\frac{I_{T}^{0}}{I_{T}}\right)\frac{dI_{T}}{\mathrm{dt}}+\frac{dM_{T}}{\mathrm{dt}}+\frac{\mathrm{dB}}{\mathrm{dt}}=\left(1-\frac{I_{T}^{0}}{I_{T}}\right)\left[\rho Y_{T}I_{T}-\left(\mu +\tau +\delta \right)I_{T}\right]+\left(\xi I_{T}+qD_{T}-\mu M_{T}\right)+\left(\delta I_{T}-\xi I_{T}+\mu M_{T}-\mathrm{\omega B}\right)$$

$$=\left(1-\frac{I_{T}^{0}}{I_{T}}\right)\left[\rho Y_{T}I_{T}-\left(\mu +\tau +\delta \right)I_{T}\right]+qD_{T}+\delta I_{T}-\mathrm{\omega B}=\left(1-\frac{I_{T}^{0}}{I_{T}}\right)\left[\left(\mu +\tau +\delta \right)I_{T}\left(\frac{\rho }{\left(\mu +\tau +\delta \right)}-1\right)\right]+qD_{T}+\delta I_{T}-\mathrm{\omega B}$$

$$=\left(1-\frac{I_{T}^{0}}{I_{T}}\right)\left[\left(\mu +\tau +\delta \right)I_{T}\left(R_{0}-1\right)\right]+qD_{T}+\delta I_{T}-\mathrm{\omega B}$$

$$=\left(\mu +\tau +\delta \right)\left(R_{0}-1\right)(\left(1-\frac{I_{T}^{0}}{I_{T}}\right)I_{T}+qD_{T}+\delta I_{T}-\mathrm{\omega B}$$

$$∴\frac{dL_{1}}{\mathrm{dt}}=-\left(\mu +\tau +\delta \right)\left(1-R_{0}\right)(\left(1-\frac{I_{T}^{0}}{I_{T}}\right)I_{T}+qD_{T}+\delta I_{T}-\mathrm{\omega B}$$

Thus we have $\frac{dL_{1}}{\mathrm{dt}}<0$ if $R_{0}\leq 0$ for all ($I_{T},M_{T},B)\neq \left(I_{T}^{0},00\right)$. Also $\frac{dI_{T}}{\mathrm{dt}}=0$ if and only if ($I_{T},M_{T},B)=\left(I_{T}^{0},00\right)$. Hence the La sale invariance principle states that the point$\left(I_{T}^{0}\right)$ of the system (1) is GAS.

### 3.9 Backward bifurcation

Theorem 5 At the stage where the backward bifurcation (Saha and Ghosh, 2021; Saha et al., 2025) parameter $\rho =\left(\mu +\tau +\delta \right)$is introduced, the model (2.1) shows backward bifurcation at $\;R_{0}=$1.

***Proof*:** Let, $\;x_{1}=Y_{T},I_{T}=x_{2},T_{T}=x_{3},D_{T}=x_{4},M_{T}=x_{5}\;\mathrm{and}\;B=x_{6}$. Afterwards, the model given by Eq. (2.1) is written as,

(3.8) $$\left\{\begin{array}{c} \frac{d\;x_{1}}{\mathrm{dt}}=\Lambda -\rho x_{1}\;x_{2}-\delta \;x_{1}\\ \frac{d\;x_{2}}{\mathrm{dt}}=\rho x_{1}\;x_{2}-\left(\mu +\tau +\delta \right)\;x_{2}\\ \frac{d\;x_{3}}{\mathrm{dt}}=\tau \;x_{2}-\eta \;x_{3}\\ \frac{d\;x_{4}}{\mathrm{dt}}=\left(1-\rho \right)x_{1}-\left(\mu +q\right)x_{4}\\ \frac{d\;x_{5}}{\mathrm{dt}}=\xi x_{2}+qx_{4}-\mu x_{5}\\ \frac{d\;x_{6}}{\mathrm{dt}}=\delta x_{2}-\xi x_{2}+\mu x_{5}-\omega x_{6} \end{array}\right)$$

Basic reproduction number,$\;R_{0}=\frac{\rho }{\left(\mu +\tau +\delta \right)}$

The transmission rate ρ is taken into consideration as a bifurcation parameter so that $\;R_{0}=$1 if and only if$\;\rho =\rho^{*}=\left(\mu +\tau +\delta \right).$

The linearization matrix of the Eq. (3.8) at the equilibrium point where tomato bacterial wilt is absent ($E_{0}$) is provided as follows:

$$J\left(E_{0}\right)=\left[\begin{array}{cccccc} -\delta & 0 & 0 & 0 & 0 & 0\\ 0 & \rho \frac{\Lambda }{\delta }-\left(\mu +\tau +\delta \right) & 0 & 0 & 0 & 0\\ 0 & \tau & -\eta \; & 0 & 0 & 0\\ \left(1-\rho \right) & 0 & 0 & -\left(\mu +q\right) & 0 & 0\\ 0 & \xi & 0 & q & -\mu & 0\\ 0 & \delta -\xi & 0 & 0 & \mu & -\omega \end{array}\right]$$

It establishes the system of equations, including the following: we look at the system $J_{w}=0$ to identify the right eigenvector$\;w={\left(w_{1},w_{2},w_{3},w_{4},w_{5},w_{6}\right)}^{T}$.

(3.9) $$\left\{\begin{array}{c} -\delta w_{1}=0\\ \left(\rho -\left(\mu +\tau +\delta \right)\right)w_{2}=0\\ \tau w_{2}-\eta w_{3}=0\\ \left(1-\rho \right)w_{1}-\left(\mu +q\right)w_{4}=0\\ \xi w_{2}+qw_{4}-\mu w_{5}=0\\ \left(\delta -\xi \right)w_{2}+\mu w_{5}-\omega w_{6}=0 \end{array}\right)$$

Attempting to solve Eq. (3.8), that we get$\;w_{1}=1=w_{2},w_{3}=\frac{\tau }{\eta }$, $w_{4}=\frac{\left(1-\rho \right)}{\left(\mu +q\right)}$, $w_{5}=\frac{\xi w_{2}+qw_{4}}{\mu }=\frac{\xi +q\frac{\left(1-\rho \right)}{\left(\mu +q\right)}}{\mu },w_{6}=\frac{\delta +q\frac{\left(1-\rho \right)}{\left(\mu +q\right)}}{\omega }$

The left eigenvector,$v={\left(v_{1},v_{2},v_{3},v_{4},v_{5},v_{6}\right)}^{T}$,is computed from $J_{v}=0,$and we obtain,

$$v_{1}=0,v_{2}=1,v_{3}=0,v_{4}=0,v_{5}=0,v_{6}=0$$

By applying the Castillo-Chavez-Song theorem, we can find the bifurcation coefficients a and b.

$$a=\sum_{k,i,j=1}^{6}v_{k}w_{i}w_{j}\frac{\partial^{2}f_{k}}{{\partial x}_{i}{\partial x}_{j}}\left(E_{0}\right),b=\sum_{k,i=1}^{6}v_{k}w_{i}\frac{\partial^{2}f_{k}}{{\partial x}_{i}\partial \rho }\left(E_{0}\right),$$

$∴a=v_{2}\left[w_{1}w_{2}\frac{\partial^{2}f_{2}}{\partial Y_{T}{\partial I}_{T}}\right]=1\left(w_{1}w_{2}.\rho \right)=\rho$, since $w_{1}=w_{2}=1$

and $b=v_{2}\left[w_{1}.\frac{\partial^{2}f_{2}}{\partial Y_{T}\partial \rho }+w_{2}\frac{\partial^{2}f_{2}}{\partial I_{T}\partial \rho }\right]=1∙\left[w_{1}I_{T}+w_{2}Y_{T}\right]$

At $E_{0},I_{T}=0,$So,$\;b=\frac{\Lambda }{\delta }$

It ultimately get: a>0and b>0.Because a and b are both positive, the model goes through a backward bifurcation. This means that the condition $R_{0}<1$ is both appropriate and sufficient to eradicate the tomato disease.

#### 3.9.1 Numerical illustration of backward bifurcation

Using the parameter values in Table 1, ρ=μ+τ+δ=0.22+0.10+0.02=0.34.

**Table 1:** The description of model parameters.

Symbol	Physical meaning	Estimated value	Units
Λ	Recruitment rate of susceptible tomatoes	1.0	plants · time^−1^
ρ	Transmission rate	0.33	**(plants · time)**^**−1**^
δ	Disease-induced death rate	0.02	day^−1^
μ	Mortality rate	0.22	day^−1^
τ	Transition rate	0.1	day^−1^
η	Removal rate of transitioning tomatoes	0.3	day^−1^
q	Development rate of diseased tomatoes	0.031	day^−1^
ξ	Contribution of infected tomatoes	0.03	day^−1^
ω	Bacterial natural death rate	0.45	day^−1^

All parameter values and supporting references are adapted from Remo et al., 2018.

$a=0.34,b=\frac{1}{0.02}=50,\beta =\mathrm{b\rho}=50\left(0.34\right)=17.$ For $c=0.01,R_{c}=1-\frac{\alpha^{2}}{4\mathrm{c\beta}}=1-\frac{{\left(0.34\right)}^{2}}{4\left(0.01\right)17}=0.83.$

Thus the numerical illustration produces the backward-bifurcation$\;0.83<R_{0}<1.$

To complement the analytical center-manifold discovery, the local bifurcation structure was investigated numerically using the third-order reduced normal form close to the critical point $R_{0}=1$. From the model parameters, we have ρ=0.34. The previously obtained Castillo-Chavez-Song coefficients satisfy a=0.34>0and b>0. For the reduced normal form we preserved a stabilizing third order term, to numerically resolve the saddle-node structure. The bifurcation diagram below, shows a secondary threshold $R_{c}\approx 0.81<1.$

Fig. 5 demonstrates the link between the fundamental reproduction number $R_{0}$ and the equilibrium infected tomato population ${I_{T}}^{*}.$In a system of equations stable equilibrium is represented by a solid curve and an unstable equilibrium by a dotted curve. When $R_{c}<1$ a saddle node bifurcation occurs. The stable disease-free equilibrium is separated from the stable endemic equilibrium by an unstable endemic branch in the interval $R_{c}<R_{0}<1.$The bistability illustrates the backward bifurcation phenomenon and shows that a slight reduction of $R_{0}$ below unity may not be sufficient to eliminate bacterial wilt in tomatoes.

**Fig. 5:**
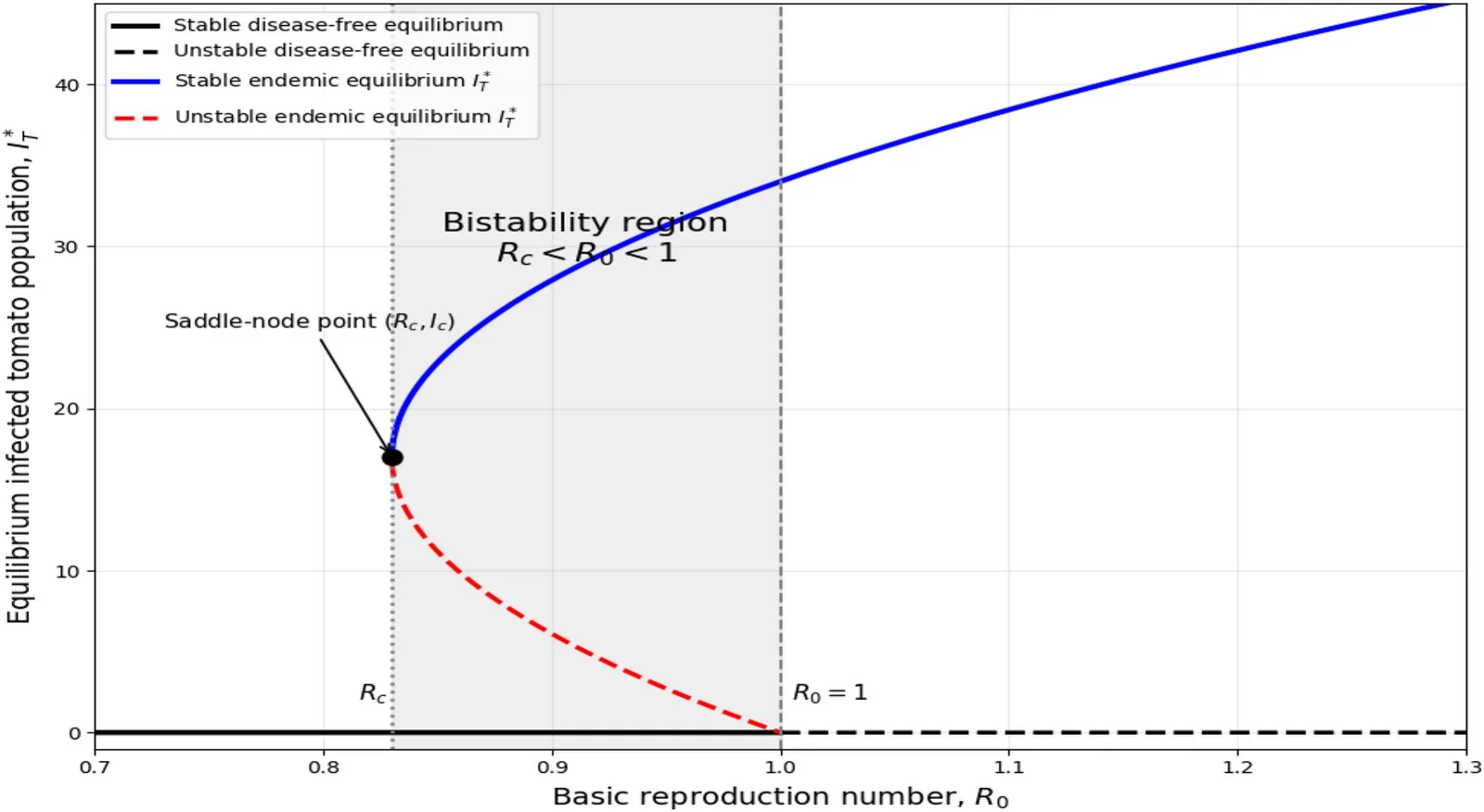
Numerical illustration of backward bifurcation in the tomato bacterial wilt model.

#### 3.9.2 Biological significance

A detailed description of the crucial biological significance. Backward bifurcation means that $R_{0}=1$is not enough to guarantee eradication. In the bistability interval $R_{c}<R_{0}<1$, the final fate is determined by the baseline sickness burden and strength of therapy. However, the system may attain a stable endemic equilibrium when the number of infected plants or the ambient disease pressure is sufficiently high, even if $R_{0}<1.$ For effective disease control, we show, for example, that it is more important to remove or treat sick plants early, manage the bacterial burden in the soil, and reduce transmission to levels below the secondary threshold Rc than to reduce $R_{0}$ slightly below 1.

## 4 Dynamics and numerical simulation of the model (2.1)

According to simulations, transmission and transition rates have a big effect on how quickly tomato bacterial wilt spreads. Good control settings help keep the system stable, while a higher transmission rate prolongs the illness.

### 4.1 Results and discussion of the model 2.1

Fig. 5 shows backward bifurcation, where stable disease-free and endemic equilibria coexist, indicating that reducing the reproduction number below unity may not guarantee elimination of tomato bacterial wilt. From the Fig. 6, the maximum of the transitional tomato population is about 6–7 plants and the minimum of the sick population is 2–3 plants. Eventually both populations revert to their ideal levels. Such behavior indicates that the disease progresses rapidly in the beginning and then stabilizes when the plants move through the different epidemiological phases. Fig. 7, Fig. 8, Fig. 9, Fig. 10 demonstrate the effect of key factors on transmission, transition, elimination, and disease development. As the transmission rate increases from 0.1 to 0.7, the susceptible tomato population $Y_{T}$ decreases sharply, indicating that the susceptible plant population is rapidly depleted under rising transmission pressure. In contrast, when the transition rate τ is increased from 0.05 to 0.6, the stability rate increases and the infected population $I_{T}$ decreases. In the same way, the transient population is much reduced when the elimination rate η is increased from 0.1 to 0.7, while the sick population is less reduced when q is increased. These simulations indicate that one method to minimize disease persistence is to reduce effective transmission and raise the rates at which plants escape infection-associated compartments.

**Fig. 6:**
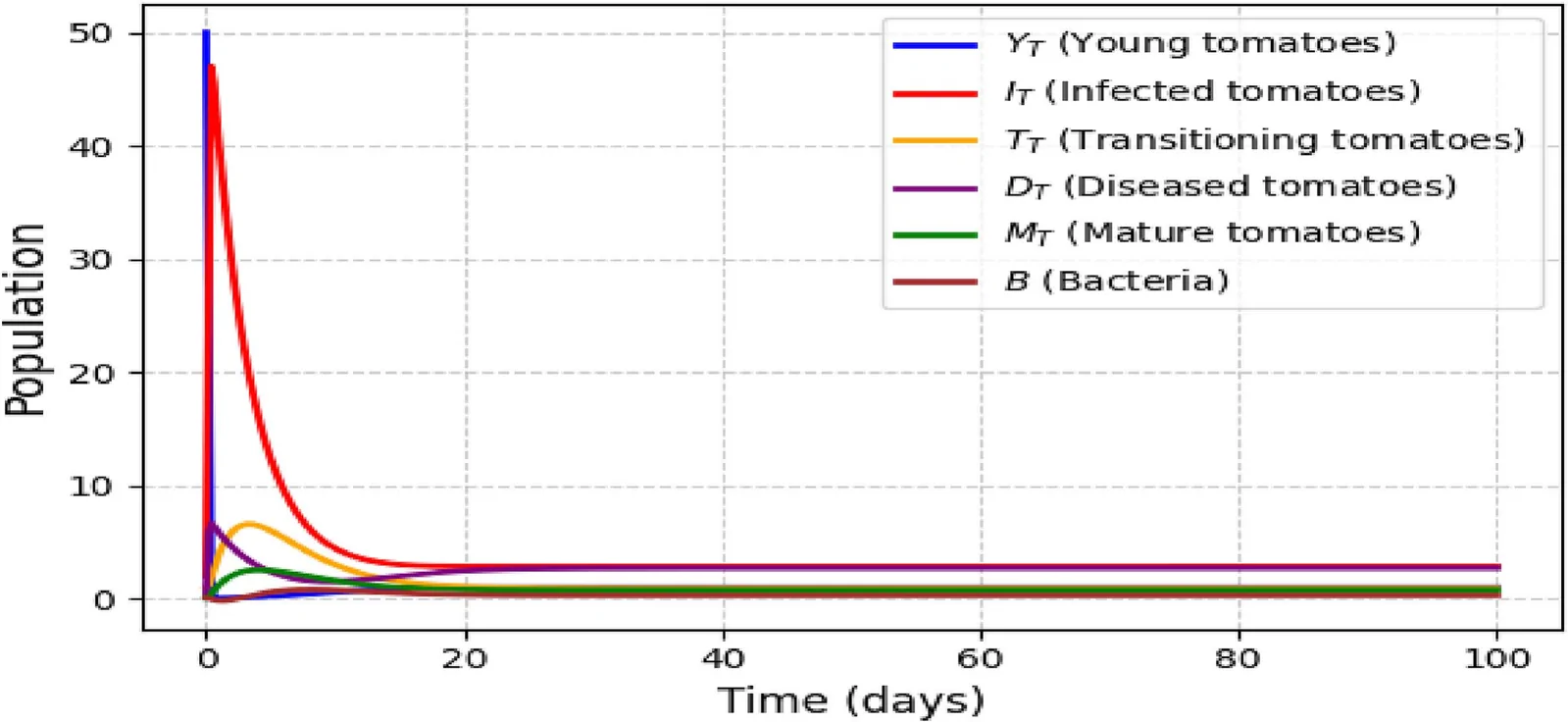
Dynamics of tomato bacterial wilt disease show how the parts of the plant and the level of bacteria in the soil vary over 100 days.

**Fig. 7:**
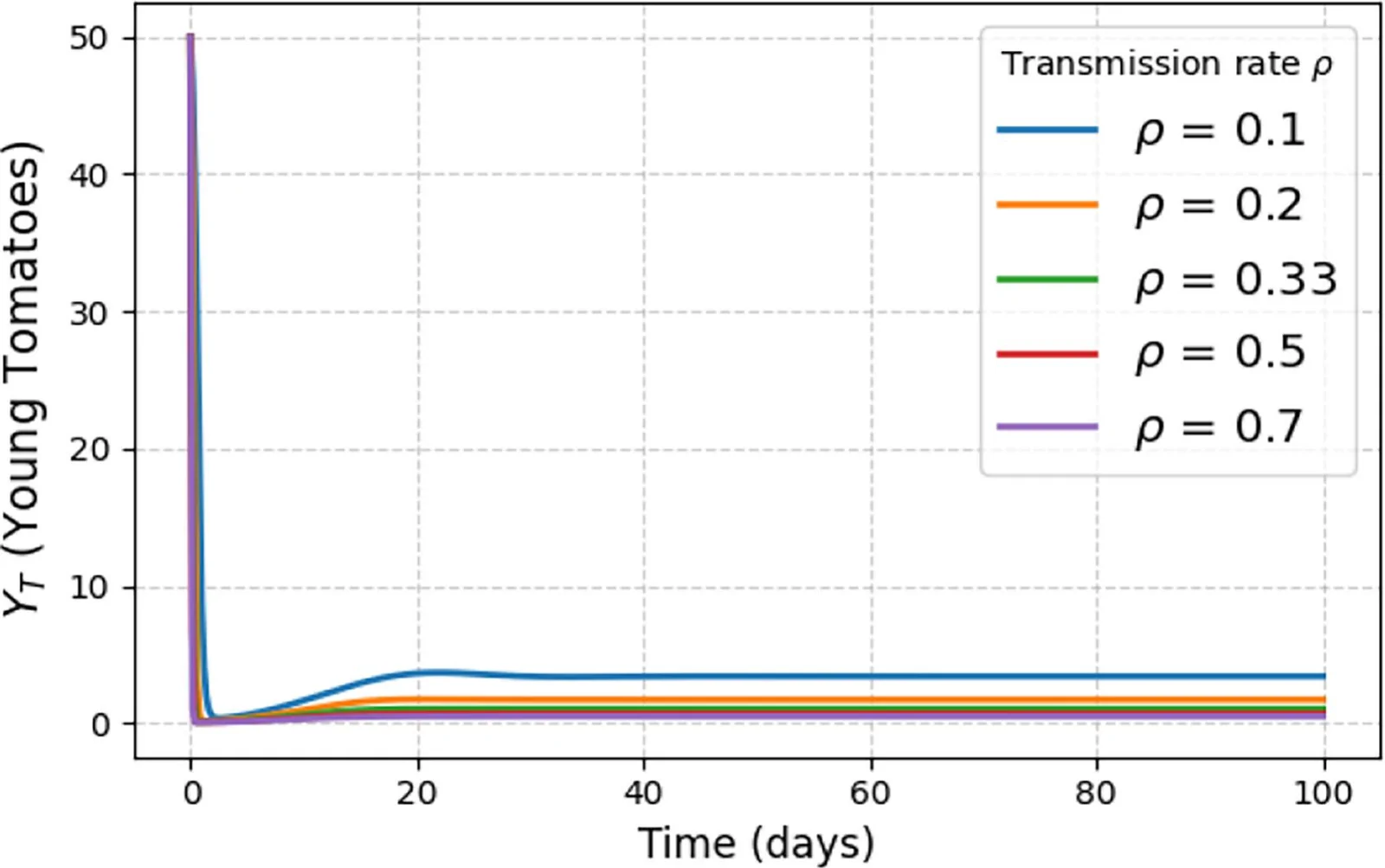
The dynamics of young tomatoes $\left(Y_{T}\right)\;$over time are analyzed, based on various transmission rates (ρ), particularlyρ=0.1,0.2,0.33,0.5,and 0.7.

**Fig. 8:**
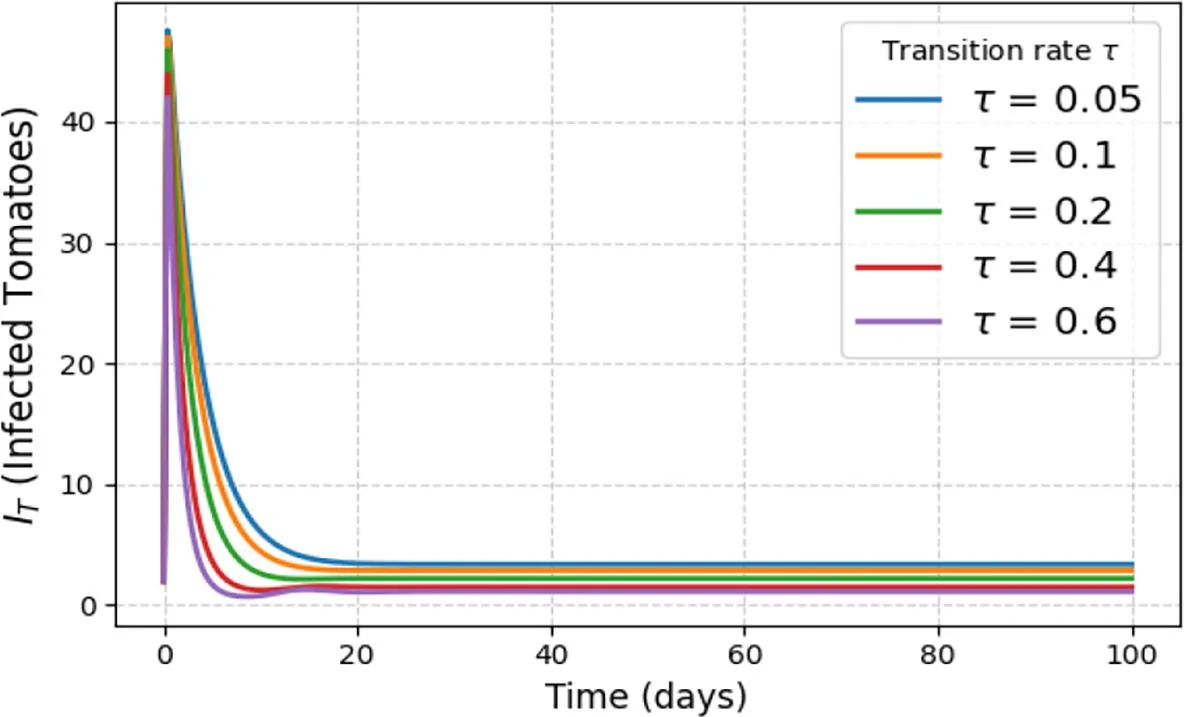
The dynamics of young tomatoes $\left(I_{T}\right)\;$over time are analyzed, based on various transition rateτ, particularlyτ=0.05,0.1,0.2,0.4,and 0.6.

**Fig. 9:**
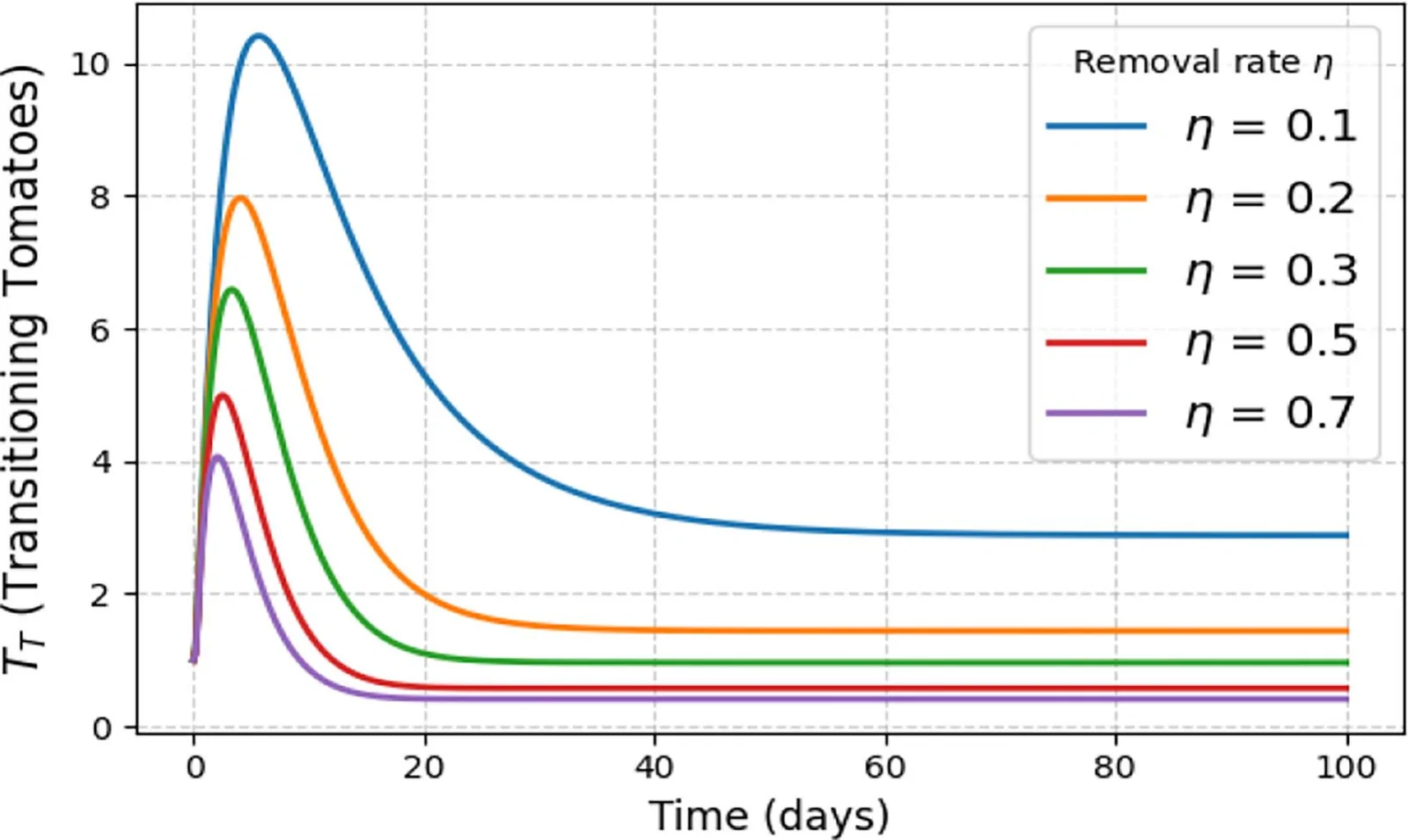
The dynamics of transitioning tomatoes $\left(T_{T}\right)\;$over time are analyzed, based on various removal rates (η), particularlyη=0.1,0.2,0.3,0.5,and 0.7.

**Fig. 10:**
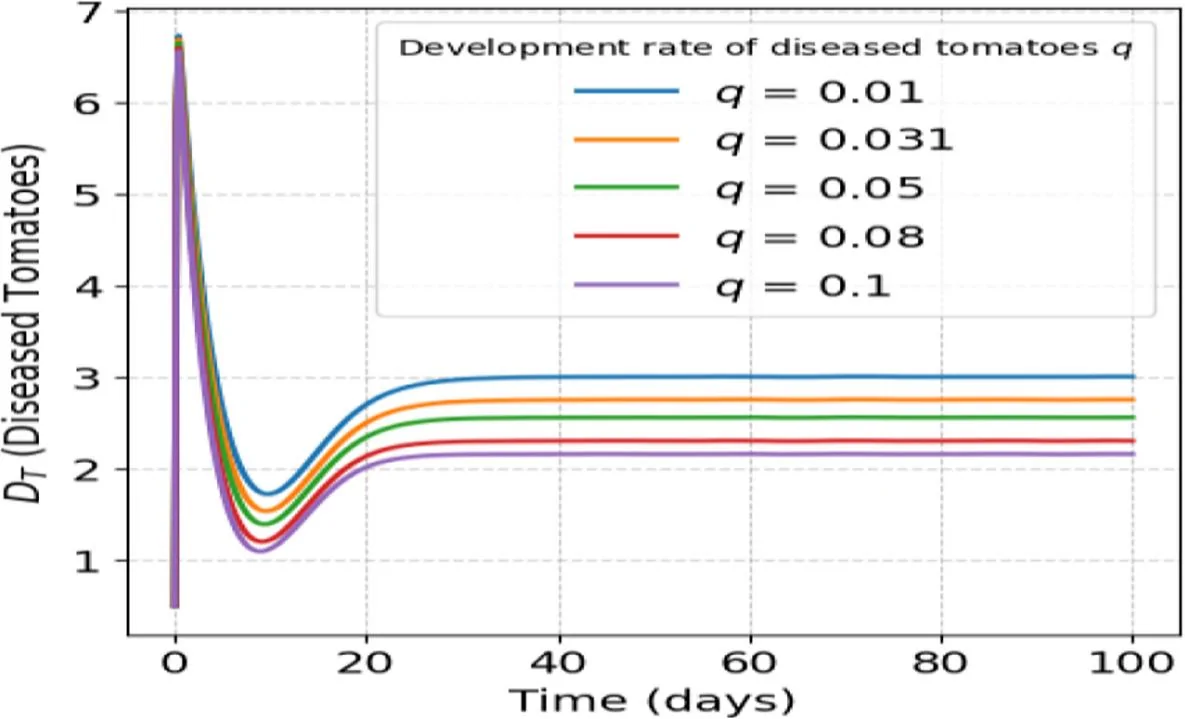
The dynamics of diseased tomatoes $\left(D_{T}\right)\;$over time are analyzed, based on various development rate of diseased tomatoes (q), particularlyq=0.01,0.031,0.05,0.08,and 0.1.

It can be shown that the peak and equilibrium levels of ripe tomato $M_{T}$ are enhanced asξ increases from 0.01 to 0.08 (Fig. 11). Changes in the disease-induced mortality parameter δ also affect the transitory bacterial population (Fig. 12). Here, we reveal indirect consequences of systems governing plant growth and decay on mature plant abundance and bacterial dynamics. The durability of the condition is represented in two different combinations of parameters in Fig. 13, Fig. 14. As can be seen in Fig. 13, the susceptible population almost gets lost in 40–50 days whereas the infected population climbs from 10 to 65 about day 20 and then drops. The vulnerable plant population drops more slowly, and the infected plant population reaches 80 plants around days 45–50 and is relatively high thereafter (Fig. 14). These findings suggest that the interaction of numerous epidemiological characteristics may considerably influence the incidence, intensity and duration of bacterial wilt disease.

**Fig. 11:**
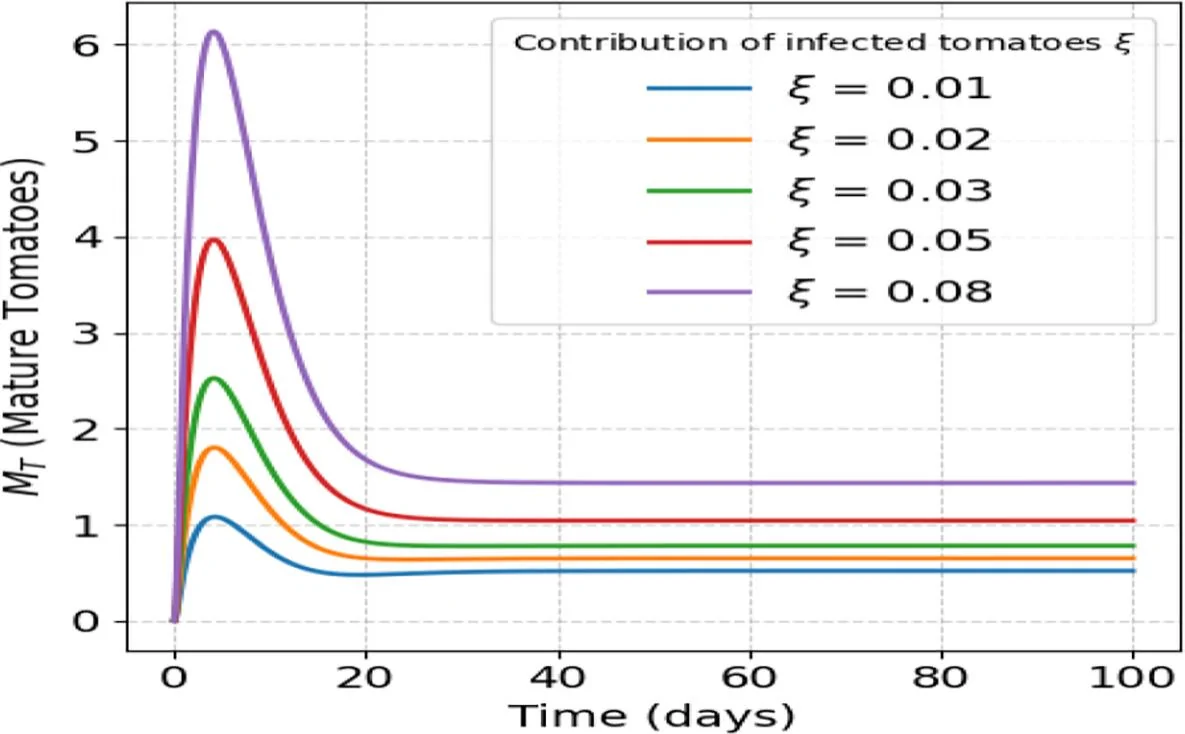
The dynamics of mature tomatoes $\left(M_{T}\right)\;$over time are analyzed, based on various contribution of infected tomatoes (ξ), particularlyξ=0.01,0.02,0.03,0.05,and 0.08.

**Fig. 12:**
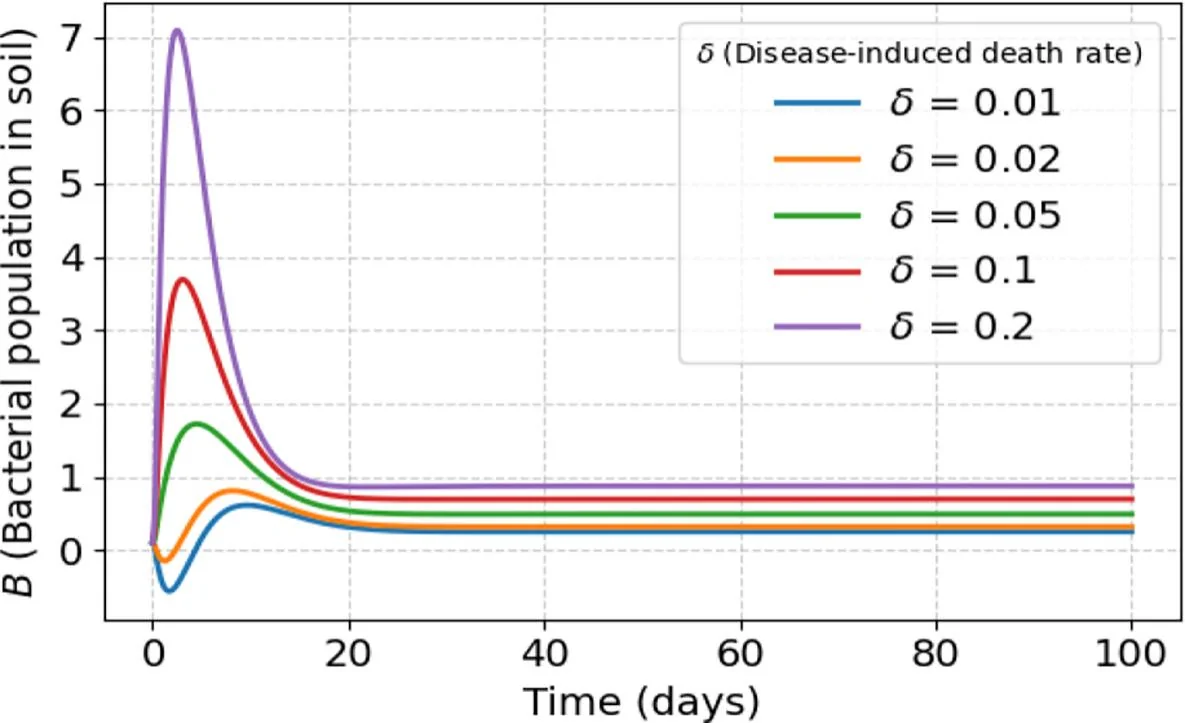
The dynamics of bacterial population in soil $\left(B\right)\;$over time are analyzed, based on various removal rates (η), particularlyB=0.01,0.02,0.05,0.1,and 0.2.

**Fig. 13:**
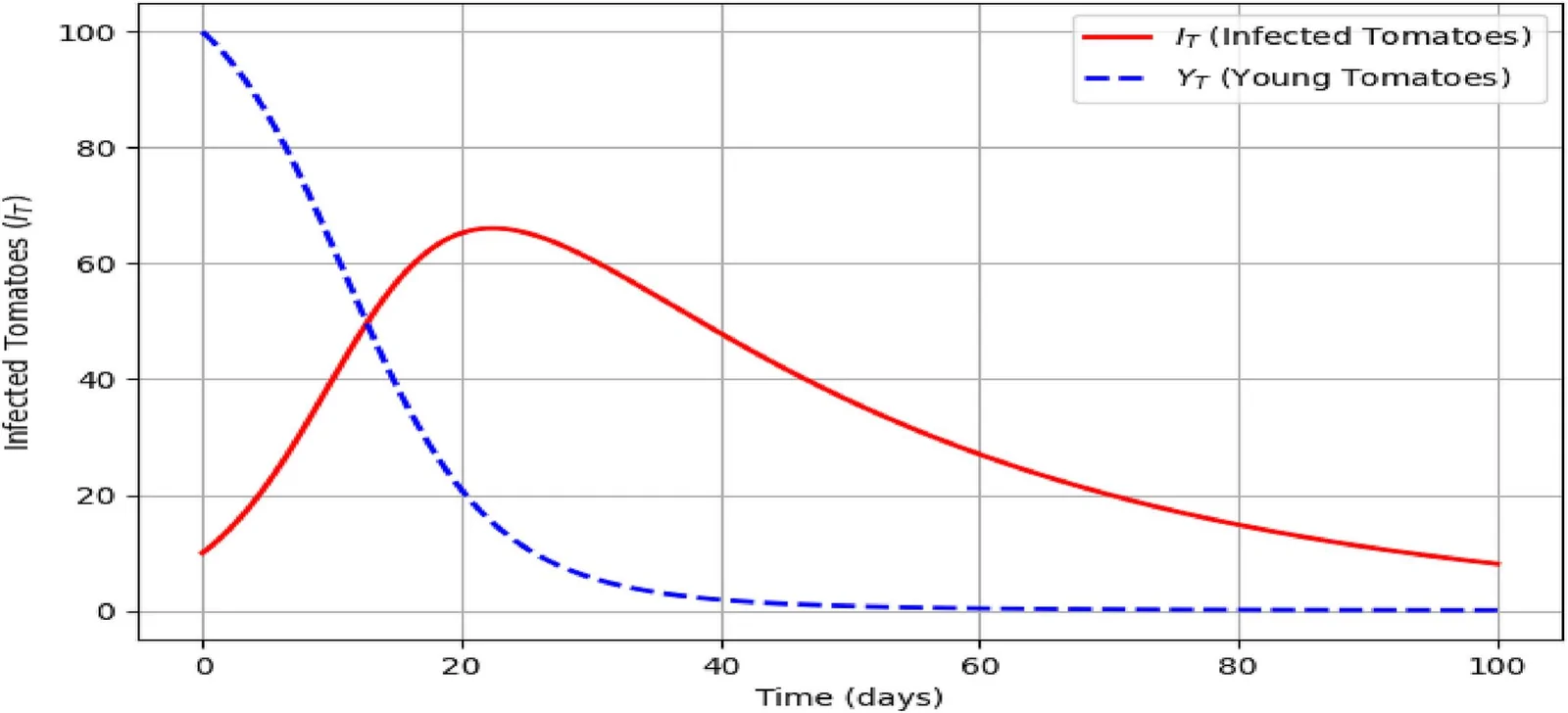
Dynamics of Infected Tomatoes ($I_{T})$ over time with ρ = 0.002, δ = 0.005,μ=0.01andτ=0.02.

**Fig. 14:**
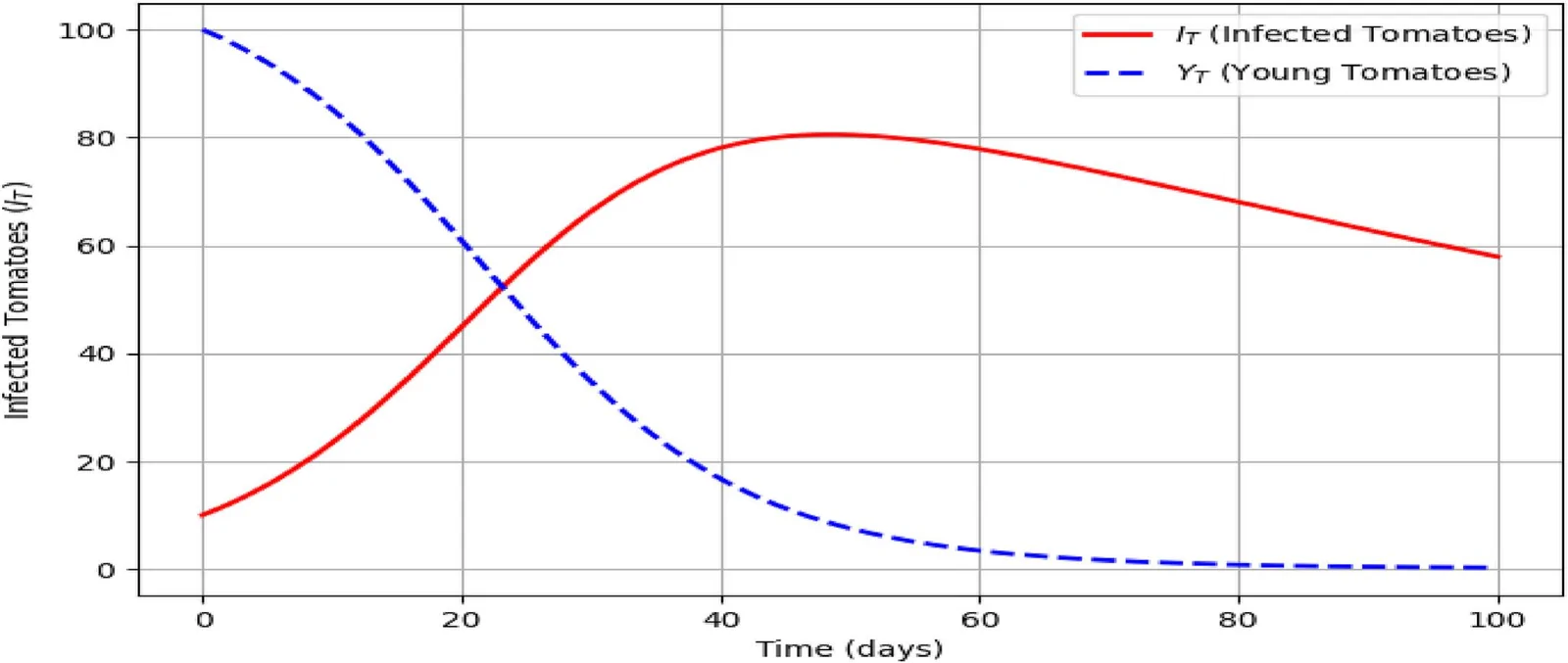
Dynamics of Infected Tomatoes ($I_{T})$ over time with ρ = 0.001, δ = 0.0005, μ=0.004andτ=0.004.

The determinants of mortality and their contribution to fully ripe tomatoes are shown in Fig. 15, Fig. 16, respectively. Under a moderate contribution rate and a low mortality rate, the mature population increases and reaches a high steady state. Higher mortality coupled with a relatively low contribution rate has led to a substantially diminished mature population. Hence, the long-term supply of mature plants depends significantly on the balance between plant growth and mortality. The significance of persistence in soil bacteria is highlighted in Fig. 17, Fig. 18. Fig. 17 shows an increase in the population size of bacteria during the simulation, indicating that the input of microbes into the soil is greater than the natural degradation of microbes. Fig. 18 demonstrates smaller oscillations, with the bacterial population stabilizing to approximately 22–24 units. The difference between these simulations suggests that the balance between the bacterial contribution and the natural decay is an important determinant in the long-term persistence of environmental diseases. Thus, the decrease in soil microbial input and the increase in pathogen clearance may be very important strategy for preventing tomato bacterial wilt.

**Fig. 15:**
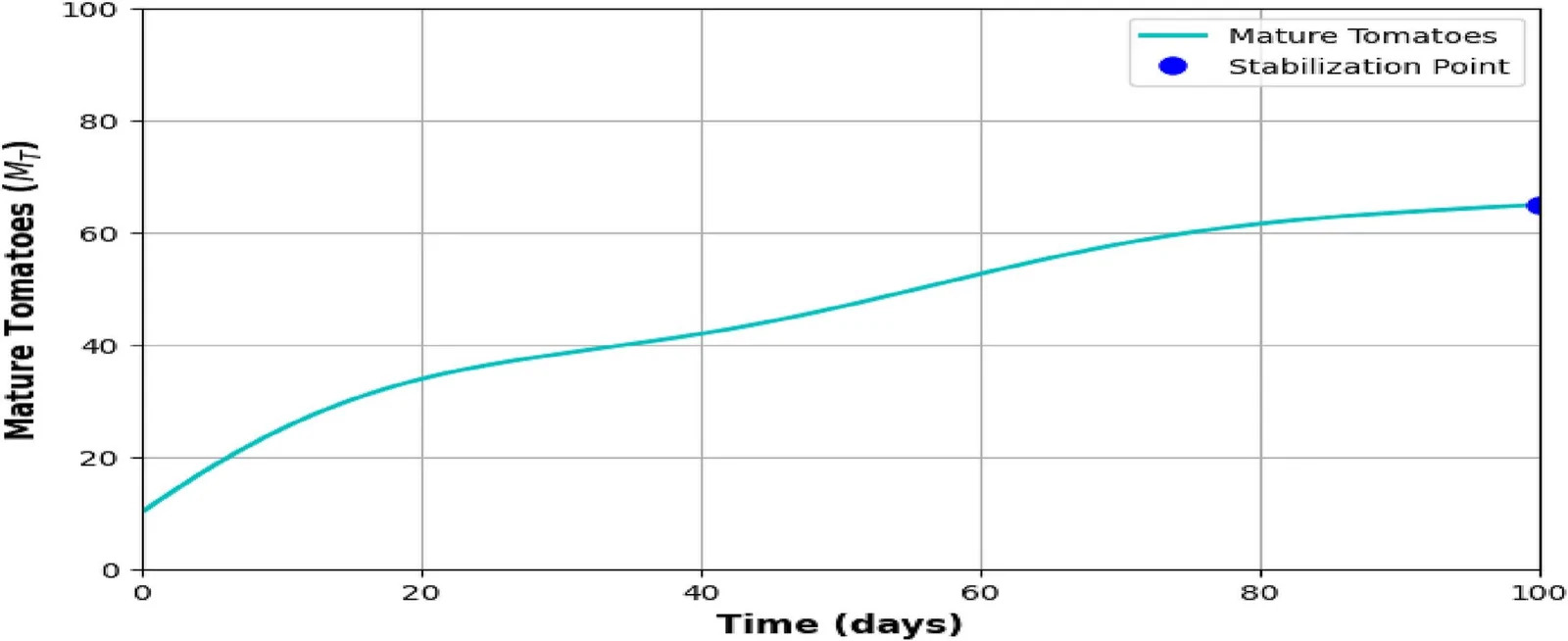
Dynamics of Mature Tomatoes ($M_{T})$ over time with ξ=0.03,μ=0.01andq=0.031.

**Fig. 16:**
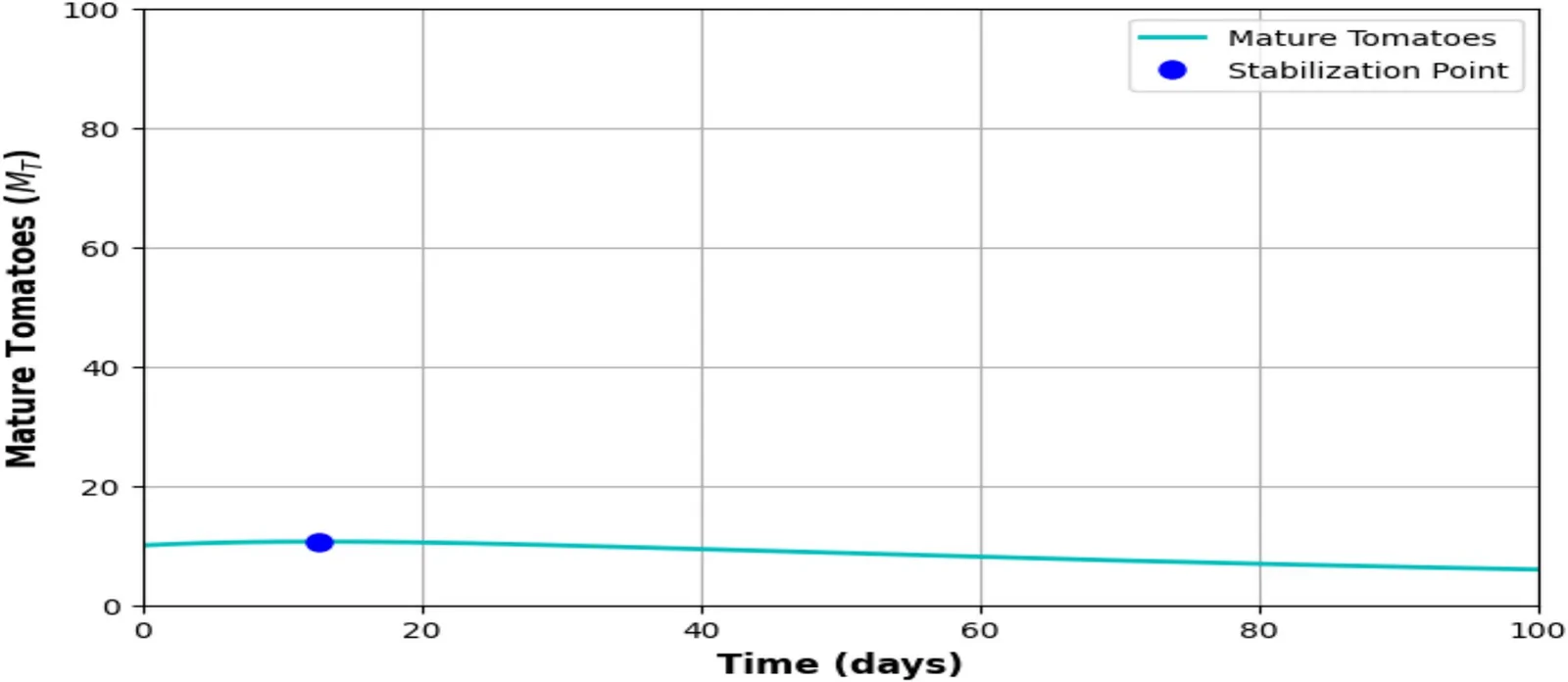
Dynamics of Mature Tomatoes ($M_{T})$ over time with ξ=0.0003,μ=0.05andq=0.0301.

**Fig. 17:**
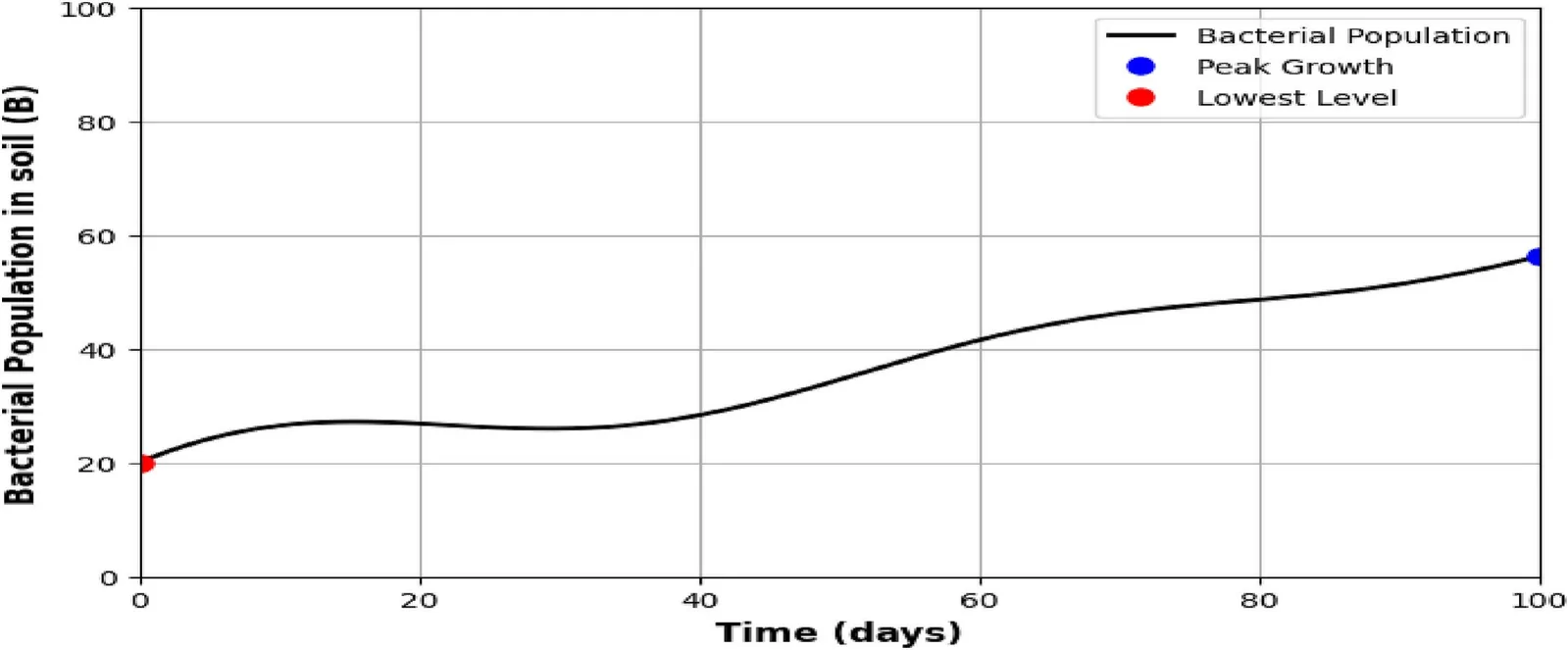
Dynamics of Bacterial population (B) over time with ξ=0.05,μ=0.05,δ=0.007andω=0.006.

**Fig. 18:**
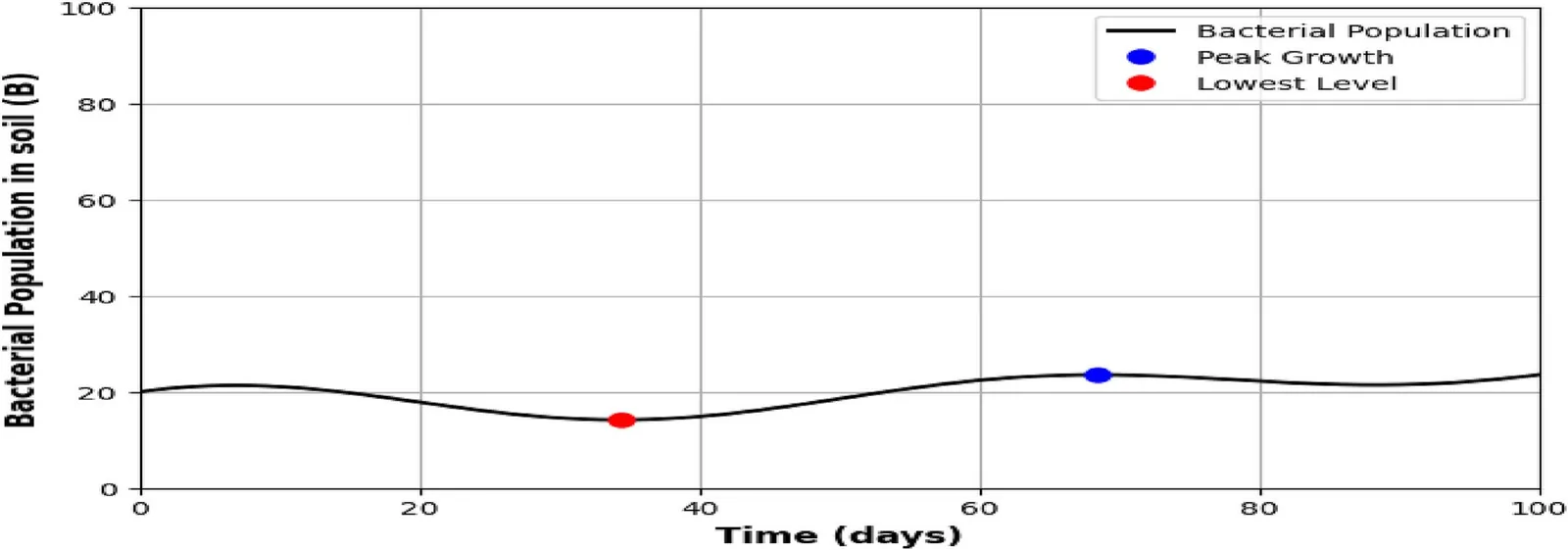
Dynamics of Bacterial population (B) over time with ξ=0.04,μ=0.03,δ=0.003andω=0.003.

Finally, the effect of changes in the transmission rate ρ on the long-term dynamics is demonstrated in Fig. 19, Fig. 20, Fig. 21. As ρ increases, the susceptible population reduces sharply, while the infected population climbs even faster. The results indicate more complex disease dynamics as transmission rates increase, especially above ρ = 0.3 in the present simulations. In the Fig. 21, variation in the dynamical regions of epidemics and endemics with changes in the bifurcation parameter. Our results emphasize the necessity of targeting transmission intensity in control interventions, given its capacity to dramatically alter disease persistence and system stability.

**Fig. 19:**
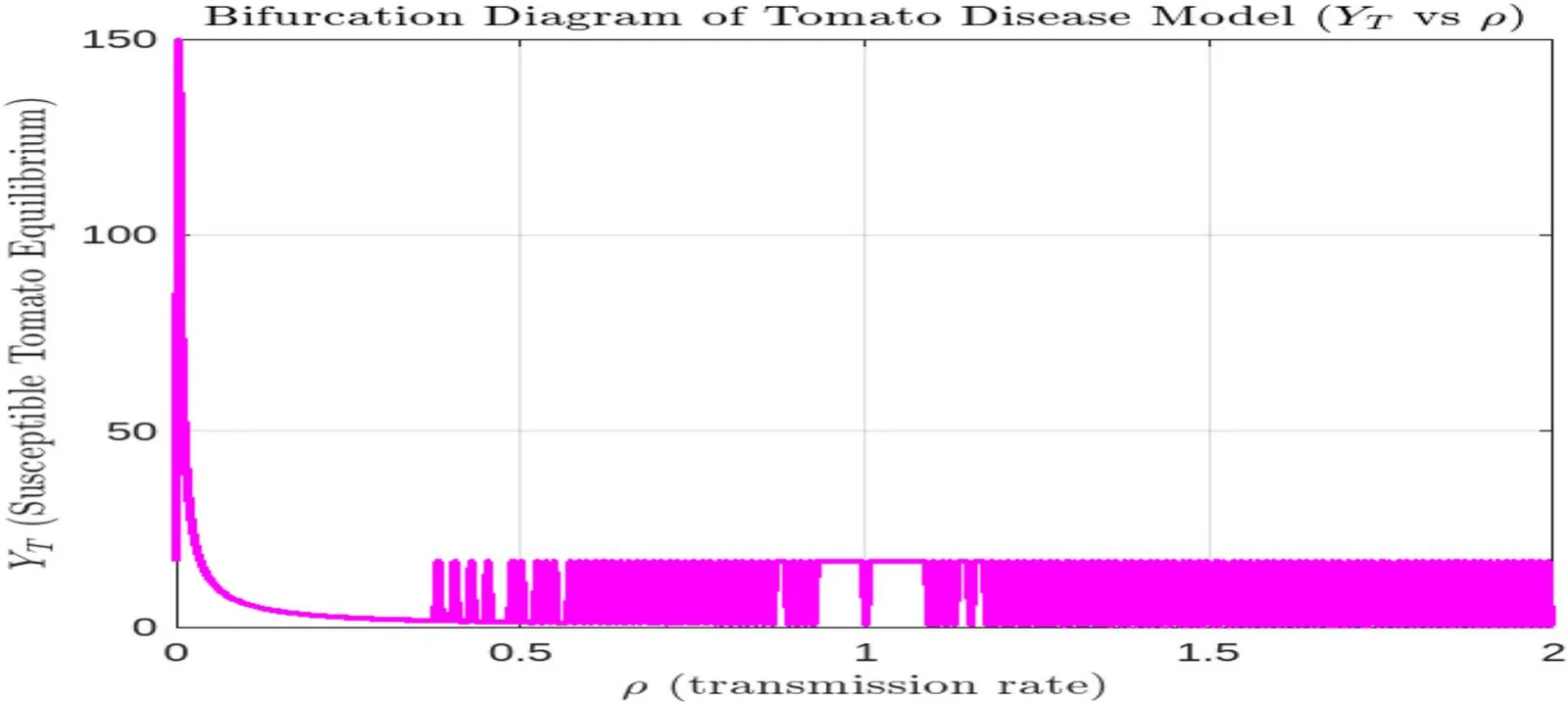
Bifurcation diagram showing the variation of the susceptible tomato population equilibrium ($Y_{T}$) with respect to the transmission rate (ρ).

**Fig. 20:**
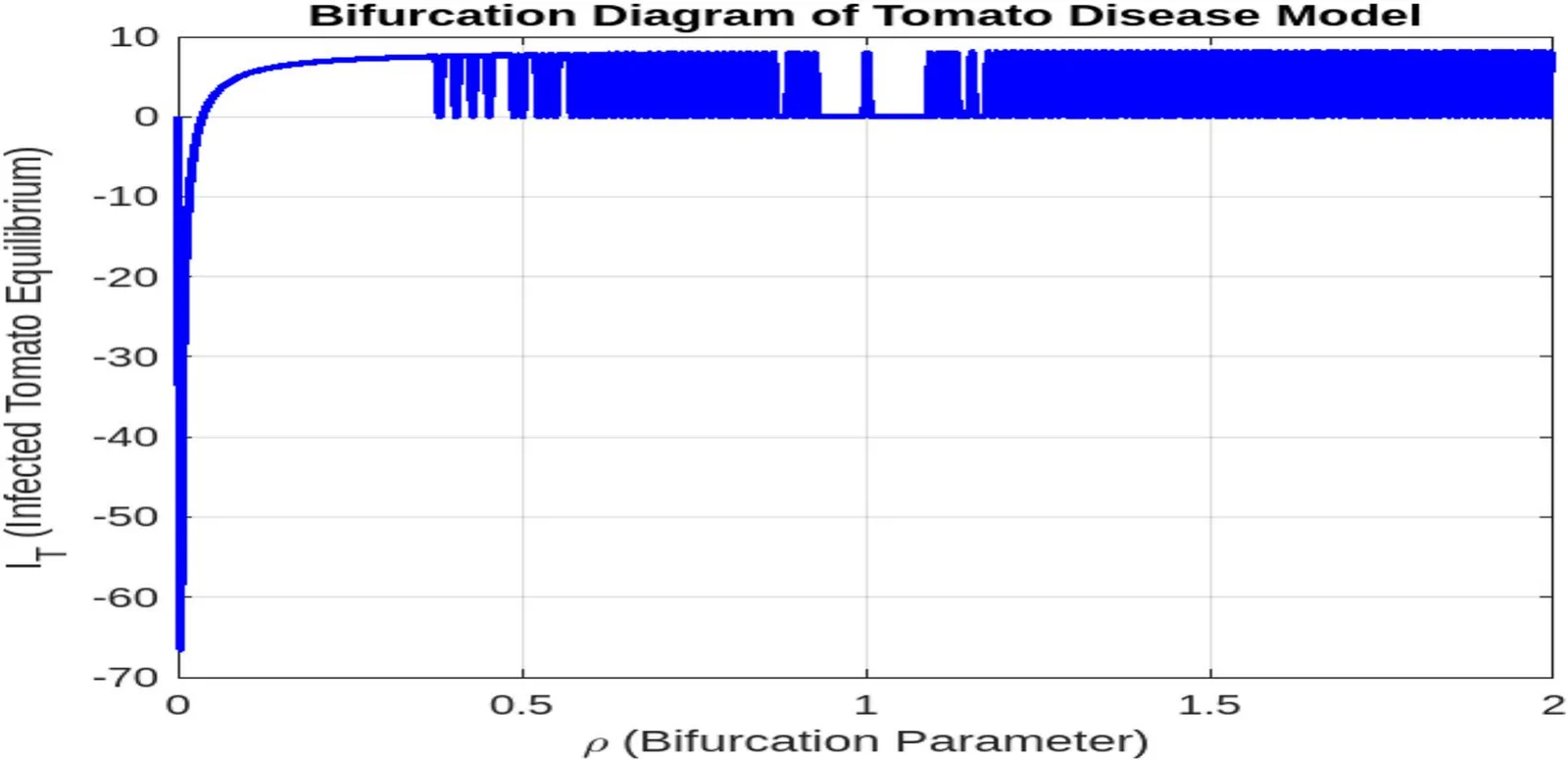
Bifurcation diagram of the infected tomato population $I_{T}$ with respect to the bifurcation parameter ρ, representing the transmission rate in a tomato disease model. $Y_{T}.$

**Fig. 21:**
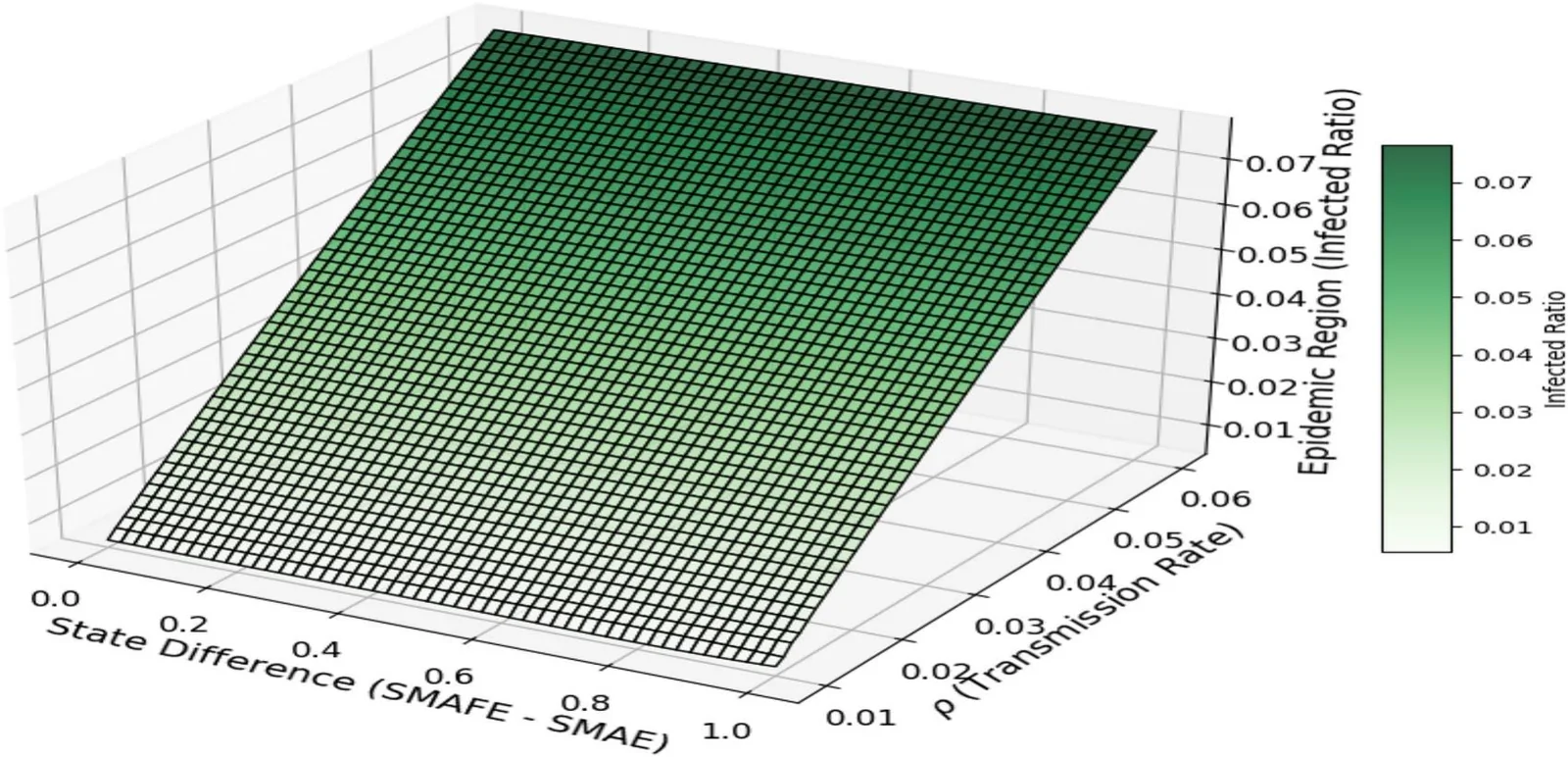
3D bifurcation diagram showing epidemic region variation with transmission rate $\left(\rho \right).$

## 5 Sensitivity analysis

To determine which model parameters are most important for controlling Tomato Wilt Virus, it uses a sensitivity analysis method to perform a bifurcation analysis. It calculates $R_{0}$ and does a sensitivity analysis here (Samui et al., 2020) based on the model parameter. The following is the formula for the sensitivity index for$\;\theta_{i}$, according to the basic reproduction number

(5.1) $$R_{0}:\Upsilon_{\theta_{i}}^{R_{0}}=\frac{\partial R_{0}}{\partial \theta_{i}}\times \frac{\theta_{i}}{R_{0}}$$

Using Fig. 22**,** Table 1 and Eq. (5.1), we calculated the $R_{0}$ sensitivity indices and presented the results in Table 2. The highest positive sensitivity index (+1) shows that when the direct transmission rate $\left(\rho \right)$ varies by 1%, the percentage change in $R_{0}$ is the same. Still, v's effect on the other variables with negative indices is the most noticeable. To stop the transmission of disease, our data show that ρ should be lower and treatment and removal rates should be higher.

**Fig. 22:**
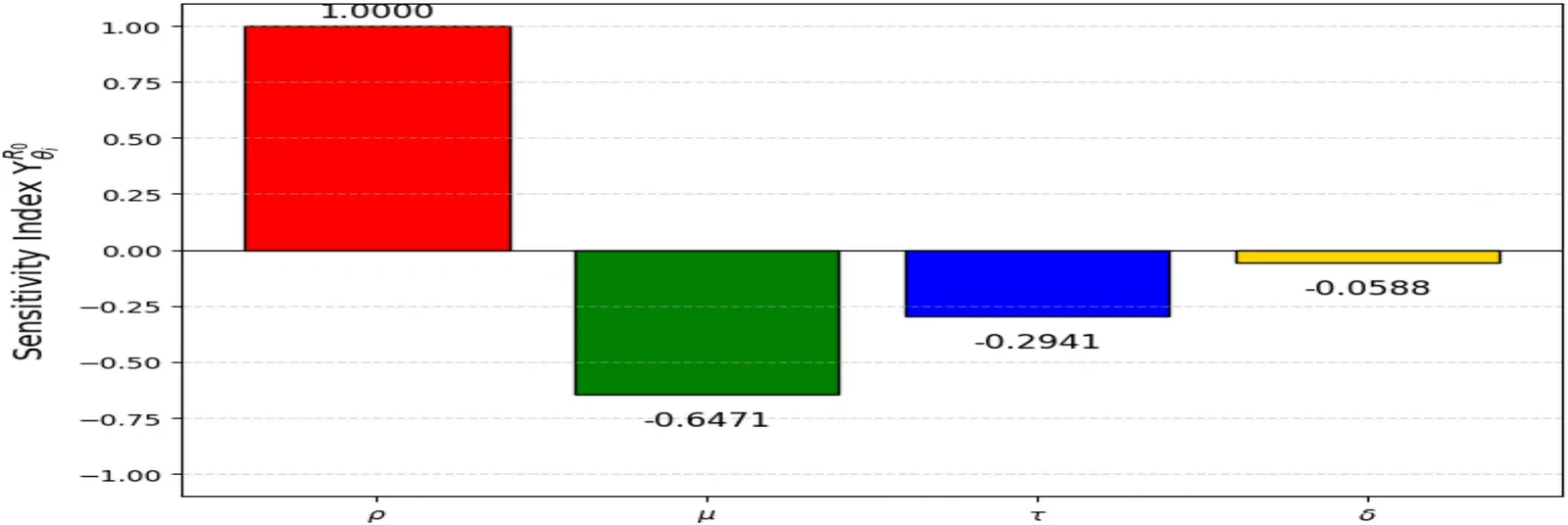
Sensitivity analysis of $R_{0}$ showing that the transmission rate ρ has the strongest positive effect, while μ, τ, and δ reduce $R_{0}$.

**Table 2:** An overview of sensitivity assessment.

Parameters	Sensitivity Index $\Upsilon_{\theta_{i}}^{R_{0}}$	Effect on $R_{0}$
ρ	+1.0000	Strongest positive impact(↑spread)
μ	−0.6471	Major negative impact(↓ spread)
τ	−0.2941	Major negative impact(↓ spread)
δ	−0.0588	Smallest negative impact

## 6 Global sensitivity analysis

**Table:** 

Parameter	PRCC value	Sensitivity effect on $R_{0}$	Interpretation
ρ	+0.925	Strong positive	Dominant driver of disease transmission; increasing ρ greatly increases $R_{0}$.
μ	−0.637	Strong negative	Higher mortality significantly reduces $R_{0}$.
τ	−0.062	Weak negative	Minor influence; faster transition slightly lowers$\;R_{0}$.
δ	+0.338	Moderate positive	Increase in δ moderately increases $R_{0}$ under global interaction effects.

The findings of the global sensitivity analysis are shown in Fig. 23, which indicates that the transmission rate$\left(\rho \right)$ primarily positively affects $R_{0}$ (PRCC=+0.925). In contrast, the mortality rate$\;\left(\mu \right)$significantly affects $R_{0}\;$negatively (−0.637). In contrast to the disease-induced death rate $\left(\delta \right)$, which has a significantly favorable influence (+0.338), the transition rate $\left(\tau \right)$ has a little negative effect (−0.062). According to the statistics, the transmission of diseases is the primary factor in their rapid spread, and the best strategy to curb infection is to raise death rates.

**Fig. 23:**
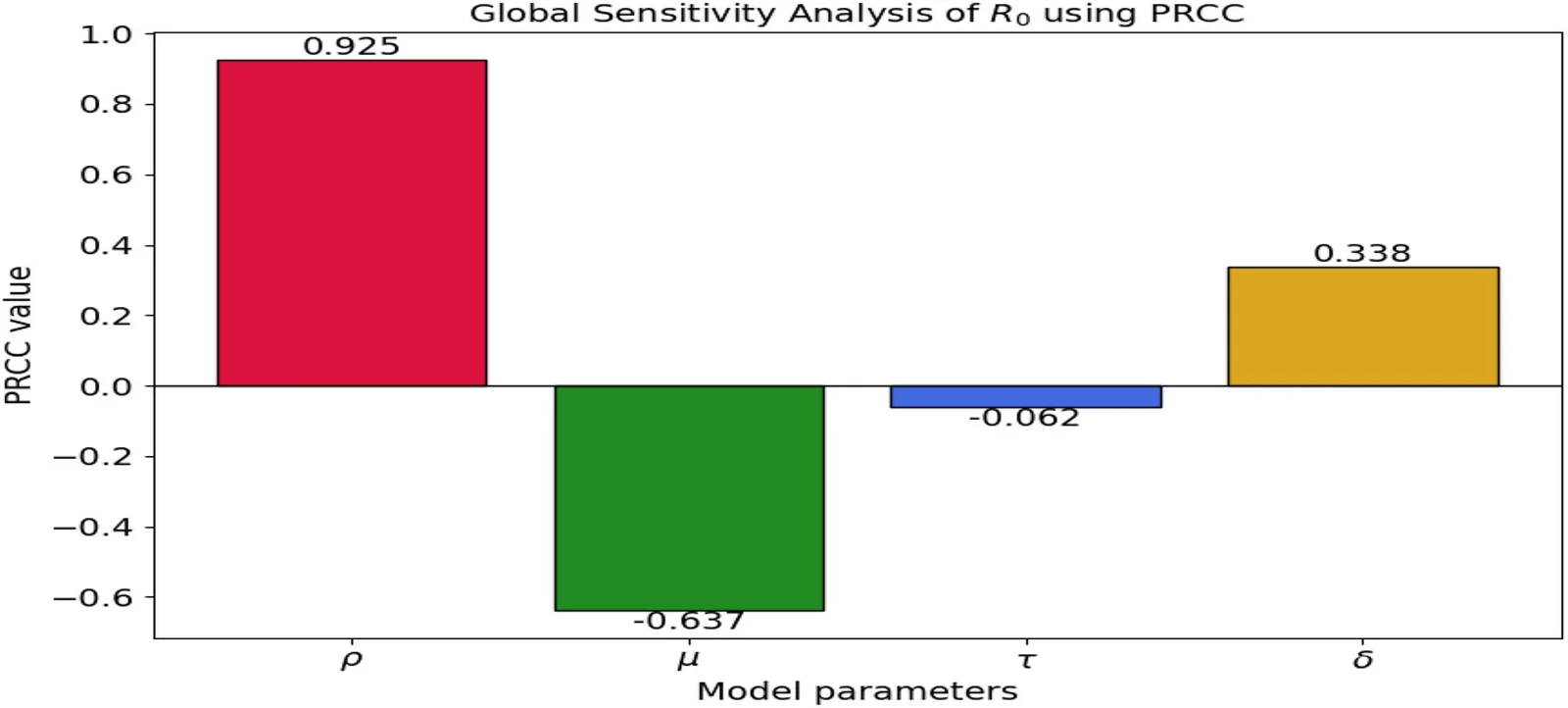
PRCC-based global sensitivity analysis of the basic reproduction number $R_{0}.$

## 7 Lyapunov exponents

It can learn a lot about the stability and dynamics of the six-dimensional system from the calculated Lyapunov exponents.

**Table:** 

Index	Lyapunov exponent $\lambda_{i}$	Interpretation
$\lambda_{1}$	−0.023	Slow contraction – dominant long-term epidemiological mode
$\lambda_{2}$	0.014	Slow contraction – secondary epidemiological mode
$\lambda_{3}$	−0.300	Intermediate damping – bacterial reduction via treatment
$\lambda_{4}$	−0.105	Intermediate contraction – plant immune recovery
$\lambda_{5}$	−0.358	Rapid stabilization – removal of diseased plants
$\lambda_{6}$	−1.138	Strong rapid stabilization – decline of soil bacteria

Fig. 24 demonstrates that all the exponents in the tomato bacterial wilt disease model are negative. This means that the system is globally asymptotically stable with the chosen parameters. This means that long-term changes in epidemiology, such as eliminating sick plants and bacteria from the soil, will occur quickly. The spectral gap between $\lambda_{1}$ and $\lambda_{2}$ indicates that the recovery processes are gradual rather than degenerative. Slow and fast dynamics are very different since the stiffness ratio $\left(\frac{\lambda_{6}}{\lambda_{1}}\approx 49.4\right)$ is very high. These results indicate that the illness can be swiftly alleviated with short-term therapy. To speed up the recovery process, long-term control needs to be achieved through continuous transmission reduction and long-term soil management.

**Fig. 24:**
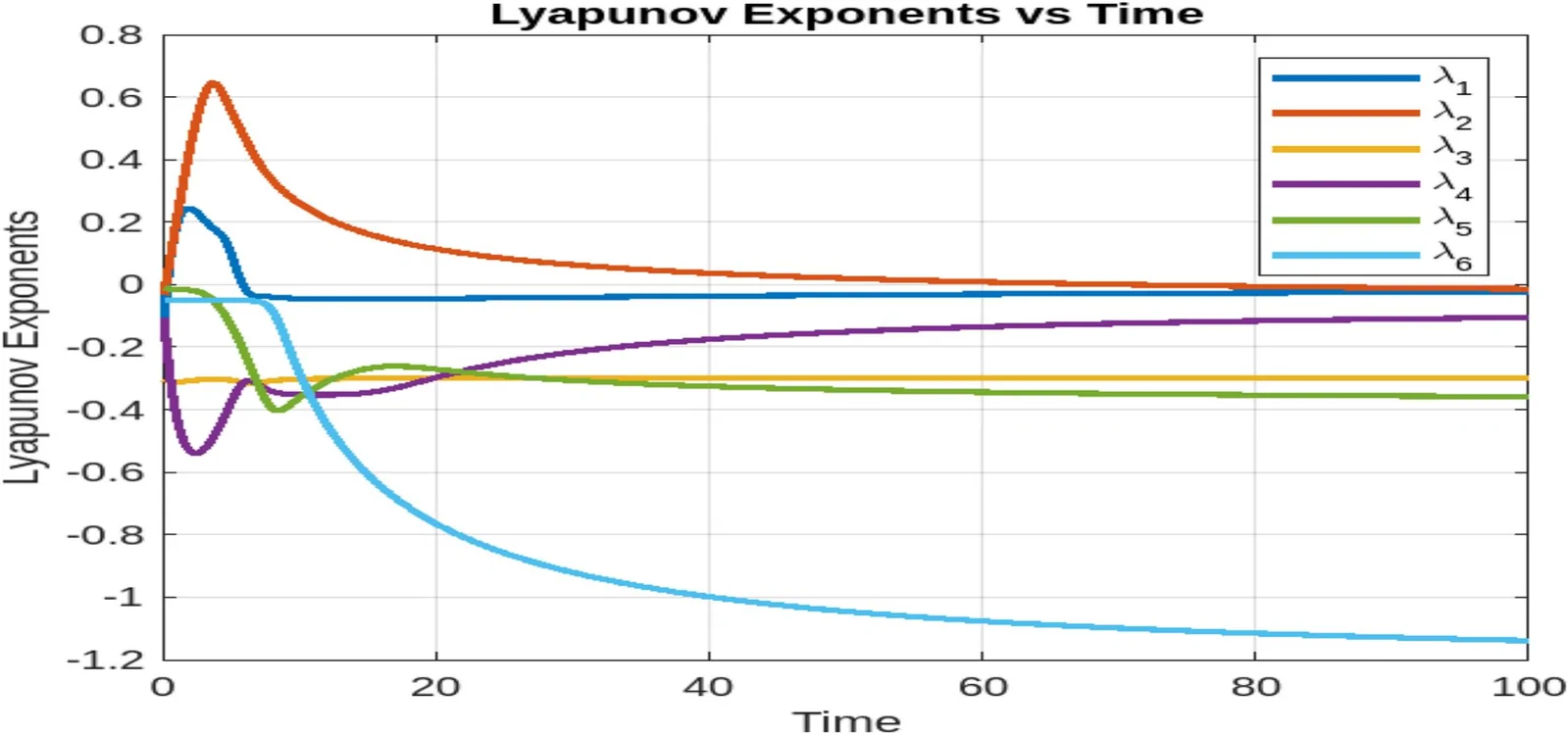
Time evolution of Lyapunov exponents $\left(\lambda_{1}-\lambda_{6}\;\right)\;$illustrating convergence to a stable fixed point.

## 8 Behavioral responses

This study examines two management approaches: control measures (C) and no controls (D). These solutions are better than doing nothing, since they give you ways to deal with tomato bacterial wilt, such as treating the plants, removing sick plants, and cleaning the soil. The predicted return that is manageable is described as

$$\pi_{C}=Y_{T}\left(\beta -C_{V}\right)+\left(1-\eta \right)\rho \left(-C_{V}-C_{I}\right)$$

$Y_{T}$ is the number of healthy tomatoes that are likely to go sick, and β is the benefit (such as yield or economic return) that healthy plants give. The equation $\left(1-\eta \right)\rho$ gives the effective infection pressure, where ρ is the transmission rate, and η is the control efficacy. The value $C_{I}$ shows the amount of money lost due to the infection. Control reduces the effects of infection, but it has costs associated with its execution; this formulation recognizes that. The anticipated return without control is given by

$$\pi_{D}=Y_{T}\left(\beta \right)+\left(1-\eta \right)\rho \left(-C_{I}\right)$$

at the same level of transmission pressure, when there is no control to prevent intervention costs, but allows infection-related losses to continue occurring. There is a relationship between the replicator equation and the evolutionary dynamics of technique adoption.

$$\frac{\mathrm{dx}}{\mathrm{dt}}=\mathrm{mx}\left(1-x\right)\left(\pi_{C}-\pi_{D}\right)$$

m is the pace of behavioral or management change, and x is the percentage of tomato systems that are under control. The term $\left(\pi_{C}-\pi_{D}\right)$tells us which way evolution is going. When the benefit of controlling something outweighs the cost of not controlling it, more people adopt a control strategy, which lowers the prevalence of the disease. If control is too expensive or doesn't work, on the other hand, non-control methods take over, and the sickness continues.

This method combines epidemiological dynamics with economic decision-making to demonstrate the importance of effective, efficient control techniques for tomato bacterial wilt.

$C_{r}=\eta =$ Control effectiveness level. $C_{r}\in \left[01\right]$

Fig. 25 shows how the control cost $C_{V}$, the control parameter $C_{r}$, the transmission rate ρ, and the efficacy η affect the adoption of equilibrium control. The fact that low-value regions are more common at higher prices shows that control adoption has dropped sharply when $C_{V}\;$rises across all panels. Cost remains the main factor in choosing a strategy, but changes in $C_{r}\;$have little effect. The transition border is somewhat altered by raising the transmission rate ρ, though it has less overall effect than cost. Similarly, there is less growth in places that are easy to regulate when control efficacy (η) increases. Findings show that the most important thing to do to get more people to use it is to minimize the cost of control. Differences in transmission intensity and efficacy also help.

**Fig. 25:**
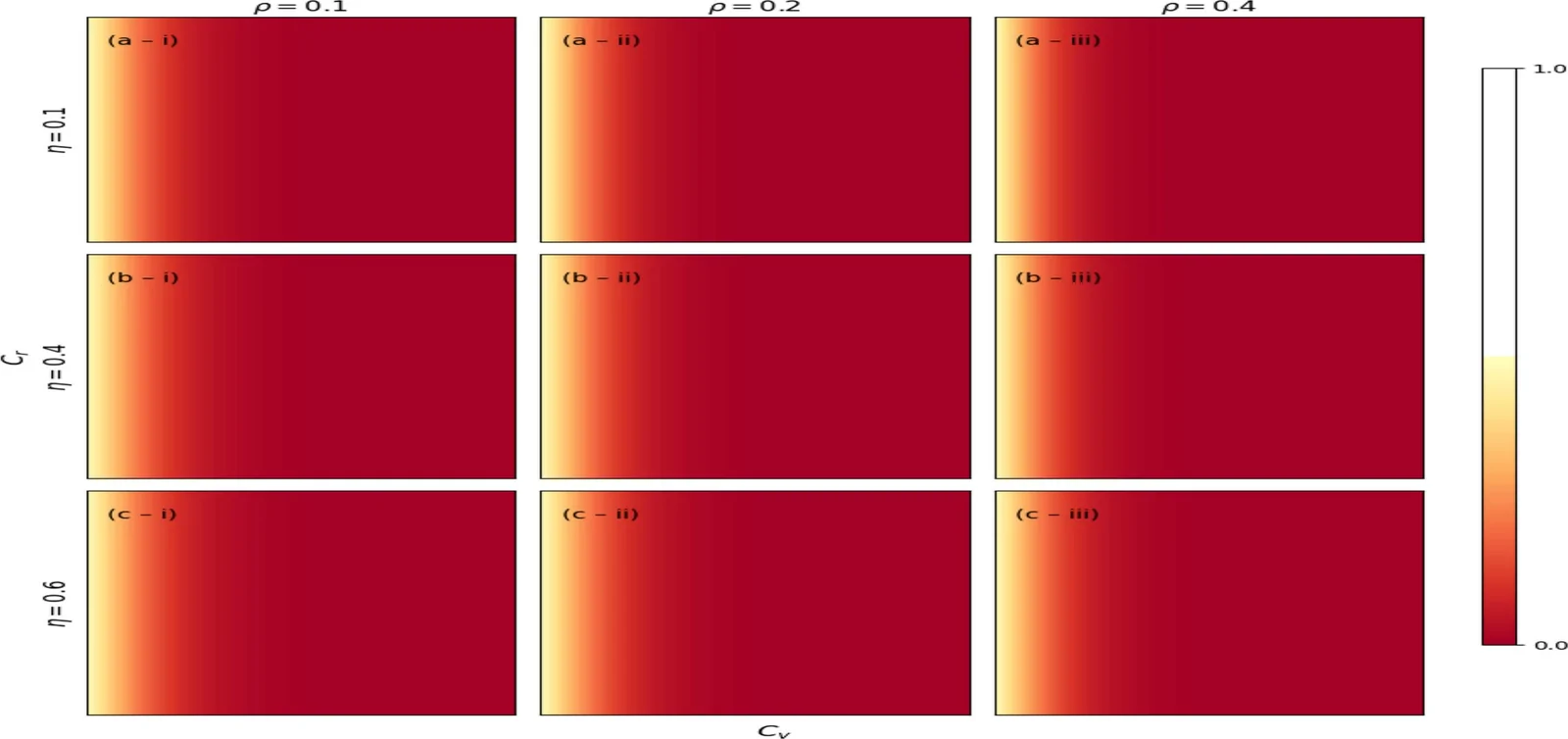
Two-dimensional heat maps of the equilibrium control adoption level in the tomato bacterial wilt model as functions of control cost $C_{V}$ (x-axis) and control-related parameter $C_{r}$ (y-axis). Rows correspond to control effectiveness levelsη=0.1 (a–i), η=0.4 (b–ii), and η=0.6 (c–iii), while columns represent transmission rates ρ=0.1 (a–i), ρ=0.2 (b–ii), andρ=0.4. (c–iii). Other parameters are fixed at baseline values consistent with the tomato bacterial wilt system.

The heat map in Fig. 26 shows that control adoption is low across most parameter ranges, except for a small area at very low $C_{V}$. Changes in the transmission rate ρ and the control efficacy η do not modify the general pattern. This suggests that the high cost of control makes it very hard to implement.

**Fig. 26:**
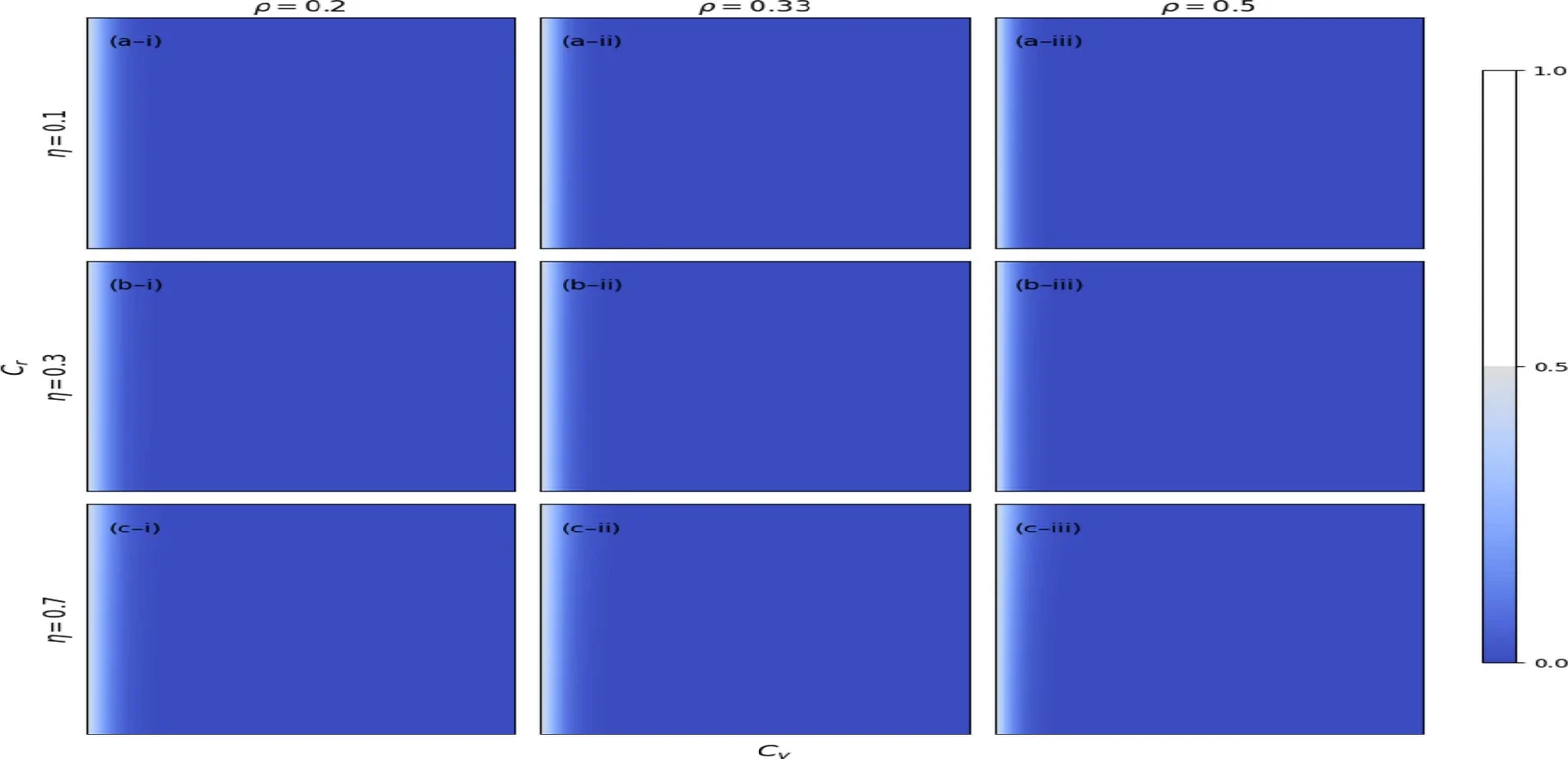
Two-dimensional heat maps of the equilibrium control adoption level in the tomato bacterial wilt model as functions of control cost $C_{V}$ (x-axis) and control-related parameter $C_{r}$ (y-axis). Rows correspond to control effectiveness levelsη=0.1 (a–i), η=0.3 (b–ii), and η=0.7 (c–iii), while columns represent transmission rates ρ=0.2 (a–i), ρ=0.33 (b–ii), andρ=0.5. (c–iii). Other parameters are fixed at baseline values consistent with the tomato bacterial wilt system.

The heat maps in Fig. 27 reveal that control adoption is essentially absent across a substantial portion of the parameter space. An outlier under this rule is a small area with a high adoption rate and a low control cost ($C_{V}$). Changes in control efficacy (η) have a small effect on this confined area, but increases in the transmission rate (ρ) make it a little wider. The results reveal that reducing control costs is very important for managing diseases. This is because the data reveal that increased control costs make it much harder for people to adopt.

**Fig. 27:**
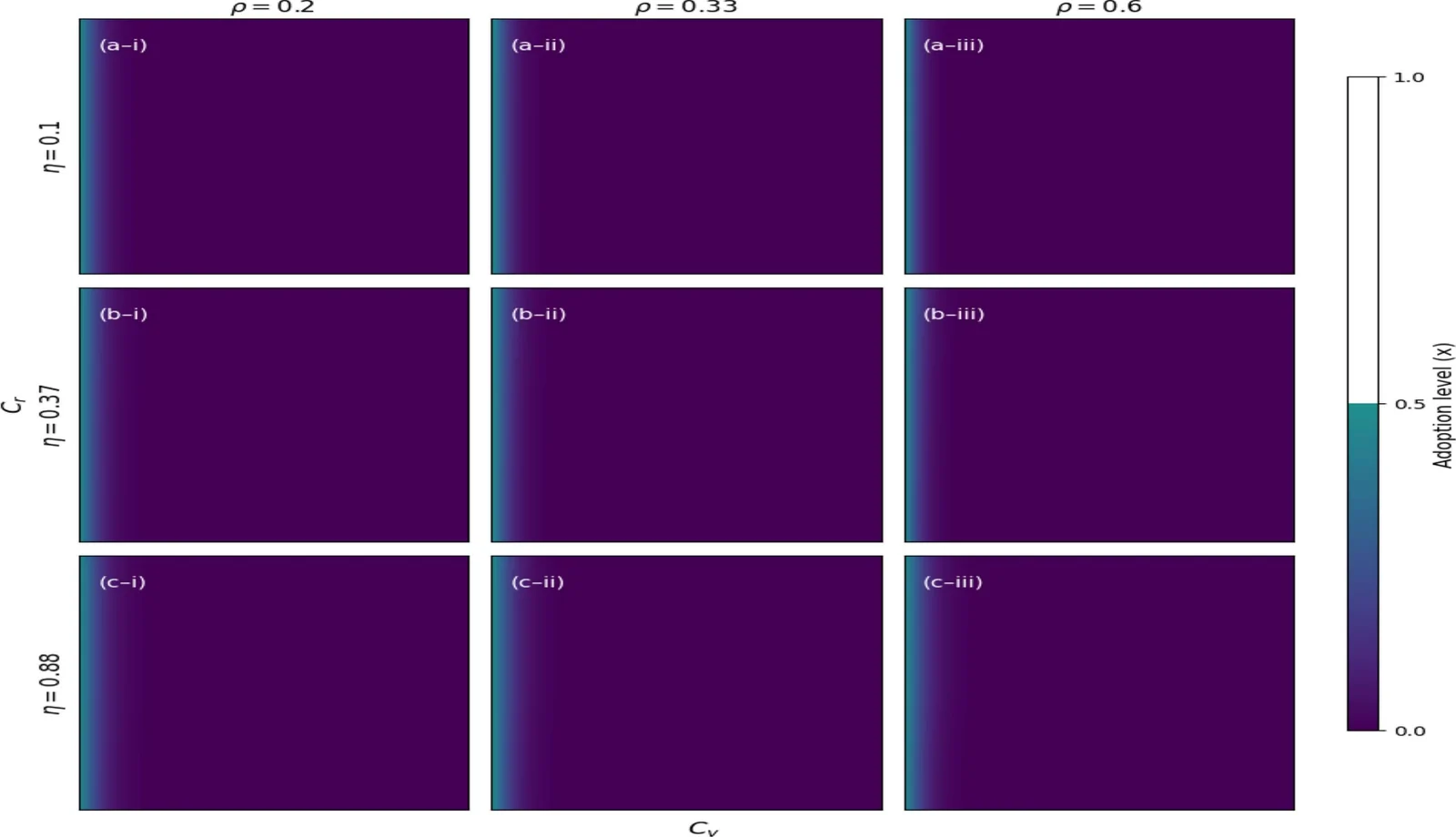
Two-dimensional heat maps of the equilibrium control adoption level in the tomato bacterial wilt model as functions of control cost $C_{V}$ (x-axis) and control-related parameter $C_{r}$ (y-axis). Rows correspond to control effectiveness levelsη=0.1 (a–i), η=0.37 (b–ii), and η=0.88 (c–iii), while columns represent transmission rates ρ=0.2 (a–i), ρ=0.33 (b–ii), andρ=0.6. (c–iii). Other parameters are fixed at baseline values consistent with the tomato bacterial wilt system.

## 9 Optimal control strategy

The six best controls are interventions that complement one another in the treatment of bacterial wilt in tomatoes. $u_{2}\left(t\right)$ assaults diseased plants to reduce their infectious burden and the disease's progression, while control $u_{1}\left(t\right)$ acts preventively, reducing effective susceptibility and transmission among new tomato plants. $u_{3}\left(t\right)$ is for the fast harvesting of transitional-stage plants. $u_{4}\left(t\right)$ targets sick plants and reduces their ability to remain in the crop. In the simulated production system, control $u_{5}\left(t\right)$ corresponds to the timely removal of mature plants, while control $u_{6}\left(t\right)$ corresponds to a direct reduction of the bacterial reservoir in the soil. Control strategies consequently include preventive measures, treatment or removal of infected plants, management of crop stages, and management of environmental infections. The numerical findings show that the appropriate intervention can accelerate the decline of the soil bacterial population and lead to a faster decrease in infected and transitional plants than in the unmanaged system. Furthermore, the optimal control profiles are maximal at the very beginning and then decrease, indicating that a management strategy with intensive early intervention followed by reduced maintenance effort would be efficient. Management of transmission, timely processing of sick and diseased plants, and suppression of the soil bacterial reservoir are required for sustained disease mitigation. To summarize, the control of bacterial wilt of tomato cannot be done with a single measure. This interpretation is based on similar research on epidemic control. It follows the broader optimal-control paradigm, using Pontryagin's maximum principle (Islam and Arman, 2026; Kamal et al., 2025) to balance the effort (or expense) of adopting measures against the reduction in disease burden. The major objectives of optimal control are the minimization of the diseased population and the expense of applied control (Saha and Ghosh, 2023; Saha et al., 2023). Optimal control theory concerns time-dependent control functions and when Eq. (1) uses the best controls, the improved model becomes clear.

(9.1) $$\left\{\begin{array}{c} \frac{dY_{T}}{\mathrm{dt}}=\Lambda -\rho \left(1-u_{1}\right)Y_{T}I_{T}-\delta Y_{T}\\ \frac{dI_{T}}{\mathrm{dt}}=\rho \left(1-u_{1}\right)Y_{T}I_{T}-\left(\mu +\tau +\delta +u_{2}\right)I_{T}\\ \frac{dT_{T}}{\mathrm{dt}}=\left(\tau +u_{2}\right)I_{T}-\left(\eta +u_{3}\right)T_{T}\\ \frac{dD_{T}}{\mathrm{dt}}=\left(1-\rho \right)Y_{T}-\left(\mu +q+u_{4}\right)D_{T}\\ \frac{dM_{T}}{\mathrm{dt}}=\xi I_{T}+qD_{T}-\left(\mu +u_{5}\right)M_{T}\\ \frac{\mathrm{dB}}{\mathrm{dt}}=\left(\delta -\xi \right)I_{T}+\mu M_{T}-\left(\omega +u_{6}\right)B \end{array}\right)$$

With initial conditions,

(9.2) $$Y_{T}\geq 0,I_{T}\geq 0,T_{T}\geq 0,D_{T}\geq 0,M_{T}\geq 0,B\geq 0$$

Where, $u_{1}\left(t\right)$=Reduce susceptibility in $Y_{T}$

$u_{2}\left(t\right)$=Treatment of infected $I_{T}$

$u_{3}\left(t\right)$=Removal from transitioning $T_{T}$

$u_{4}\left(t\right)$=Treatment of disease $D_{T}$

$u_{5}\left(t\right)$=Harvest of mature $M_{T}$

$u_{6}\left(t\right)$=Bacterial removal from soil B

Theorem 6 (Existence of Optimal Control)

Let u be the group of controls that can be used:

$u=\left\{\left(u_{1},u_{2},u_{3},u_{4},u_{5},u_{6}\right)\epsilon \left[0,u_{1,\max}\right]\times ∙∙∙\times \left[0,u_{6,\max}\right]\;\text{quantifiable}\;\mathrm{on}\;\left[0,T\right]\right\}$. Then there exist an optimal control $u_{i}^{*}=\left(u_{1}^{*},u_{2}^{*},u_{3}^{*},u_{4}^{*},u_{5}^{*},u_{6}^{*}\right)\epsilon \;u$ and corresponding optimal state trajectory$\left(Y_{T}^{*},I_{T}^{*},T_{T}^{*},D_{T}^{*},M_{T}^{*},B^{*}\right)\;$that minimizes the cost functional:$\;j=\int_{0}^{T}\left[A_{1}I_{T}+A_{2}D_{T}+A_{3}B+A_{4}M_{T}+\sum_{i=1}^{6}\frac{1}{2}c_{i}{u_{i}}^{2}\right]\;$subject to the controlled system (9.1) with (9.2).

**Proof:**

The Hamiltonian for this system is:

$$H=A_{1}I_{T}+A_{2}D_{T}+A_{3}B+A_{4}M_{T}+\sum_{i=1}^{6}\frac{1}{2}c_{i}{u_{i}}^{2}+\lambda_{1}\left(\Lambda -\rho \left(1-u_{1}\right)Y_{T}I_{T}-\delta Y_{T}\right)+\lambda_{2}\left(\rho \left(1-u_{1}\right)Y_{T}I_{T}-\left(\mu +\tau +\delta +u_{2}\right)I_{T}\right)+\lambda_{3}\left(\left(\tau +u_{2}\right)I_{T}-\left(\eta +u_{3}\right)T_{T}\right)+\lambda_{4}\left(\left(1-\rho \right)Y_{T}-\left(\mu +q+u_{4}\right)D_{T}\right)+\lambda_{5}\left(\xi I_{T}+qD_{T}-\left(\mu +u_{5}\right)M_{T}\right)+\lambda_{6}\left(\left(\delta -\xi \right)I_{T}+\mu M_{T}-\left(\omega +u_{6}\right)B\right)$$

The adjoint system is given by:$\;\frac{d\lambda_{i}}{\mathrm{dt}}=-\frac{\partial H}{\partial x_{i}},\lambda_{i}\left(t\right)=0\;\mathrm{for}\;i=1,\ldots ,6.$

Optimality conditions: $\frac{\partial H}{\partial u_{i}}=0\Rightarrow u_{i}^{*}=\min\left(\;u_{i},\max\left(0,\frac{\mathrm{expression involving}\;\lambda_{j}}{C_{i}}\right)\right)$

$$\frac{d\lambda_{1}}{\mathrm{dt}}=\lambda_{1}\left(\rho \left(1-u_{1}\right)I_{T}-\delta \right)-\lambda_{2}\rho \left(1-u_{1}\right)I_{T}-\lambda_{4}\left(1-\rho \right)$$

$$\frac{d\lambda_{2}}{\mathrm{dt}}=-A_{1}+\lambda_{1}\rho \left(1-u_{1}\right)Y_{T}-\lambda_{2}\left[\rho \left(1-u_{1}\right)Y_{T}-\left(\mu +\tau +\delta +u_{2}\right)\right]-\lambda_{3}\left(\tau +u_{2}\right)-\lambda_{5}\xi -\lambda_{5}\left(\delta -\xi \right)$$

$$\frac{d\lambda_{3}}{\mathrm{dt}}=\lambda_{3}\left(\eta +u_{3}\right)$$

$$\frac{d\lambda_{4}}{\mathrm{dt}}=-A_{2}+\lambda_{4}\left(\mu +q+u_{4}\right)-\lambda_{5}q$$

$$\frac{d\lambda_{5}}{\mathrm{dt}}=-A_{4}+\lambda_{5}\left(\mu +u_{5}\right)-\lambda_{6}\mu$$

$$\frac{d\lambda_{6}}{\mathrm{dt}}=-A_{3}+\lambda_{6}\left(\omega +u_{6}\right)$$

Transversality conditions$\;\lambda_{i}\left(t\right)=0$, for *i* = 1, 2, 3, 4, 5, 6.

Characterization of Optimal Controls: Using Pontryagin's Maximum Principle, the optimal controls are:

$$u_{1}^{*}\left(t\right)=\min\left(\max\left(0,\frac{\left(\lambda_{1}-\lambda_{2}\right)\rho Y_{T}I_{T}}{C_{1}}\right),u_{1,\max}\right)$$

$$u_{2}^{*}\left(t\right)=\min\left(\max\left(0,\frac{\left(\lambda_{2}-\lambda_{3}\right)I_{T}}{C_{2}}\right),u_{2,\max}\right)$$

$$u_{3}^{*}\left(t\right)=\min\left(\max\left(0,\frac{\lambda_{3}T_{T}}{C_{3}}\right),u_{3,\max}\right)$$

$$u_{4}^{*}\left(t\right)=\min\left(\max\left(0,\frac{\lambda_{4}D_{T}}{C_{4}}\right),u_{4,\max}\right)$$

$$u_{5}^{*}\left(t\right)=\min\left(\max\left(0,\frac{\lambda_{5}M_{T}}{C_{5}}\right),u_{5,\max}\right)$$

$$u_{6}^{*}\left(t\right)=\min\left(\max\left(0,\frac{\lambda_{6}B}{C_{6}}\right),u_{6,\max}\right)$$

## 10 Numerical simulations and discussion of the optimal control strategy for tomato disease

It used numerical simulations to test the indicated best methods for controlling tomato bacterial wilt. The study found that the disease's spread and the number of cases can be greatly reduced by implementing controls at the right time and in the right amount. These controls include steps to reduce the likelihood of plant illness, treat sick plants, remove crops that are changing, harvest, and clear soil infected with bacteria. Farmers and the economy as a whole benefit from early, flexible strategy adoption, which leads to the largest reduction in yield loss. These results show how important it is to use timely, tailored field-based adaptive management strategies to ensure that tomatoes can be grown successfully in the long term.

### 10.1 Results and discussion of the model (9.1)

According to Fig. 28, Fig. 29, $Y_{T}$ drops from around 100 to nearly 0 in 5 to 10 days, whereas $I_{T}$ drops from about 90 to nearly 0 in 10 to 15 days. After that, both variables drop to very low levels, suggesting they will soon settle.

**Fig. 28:**
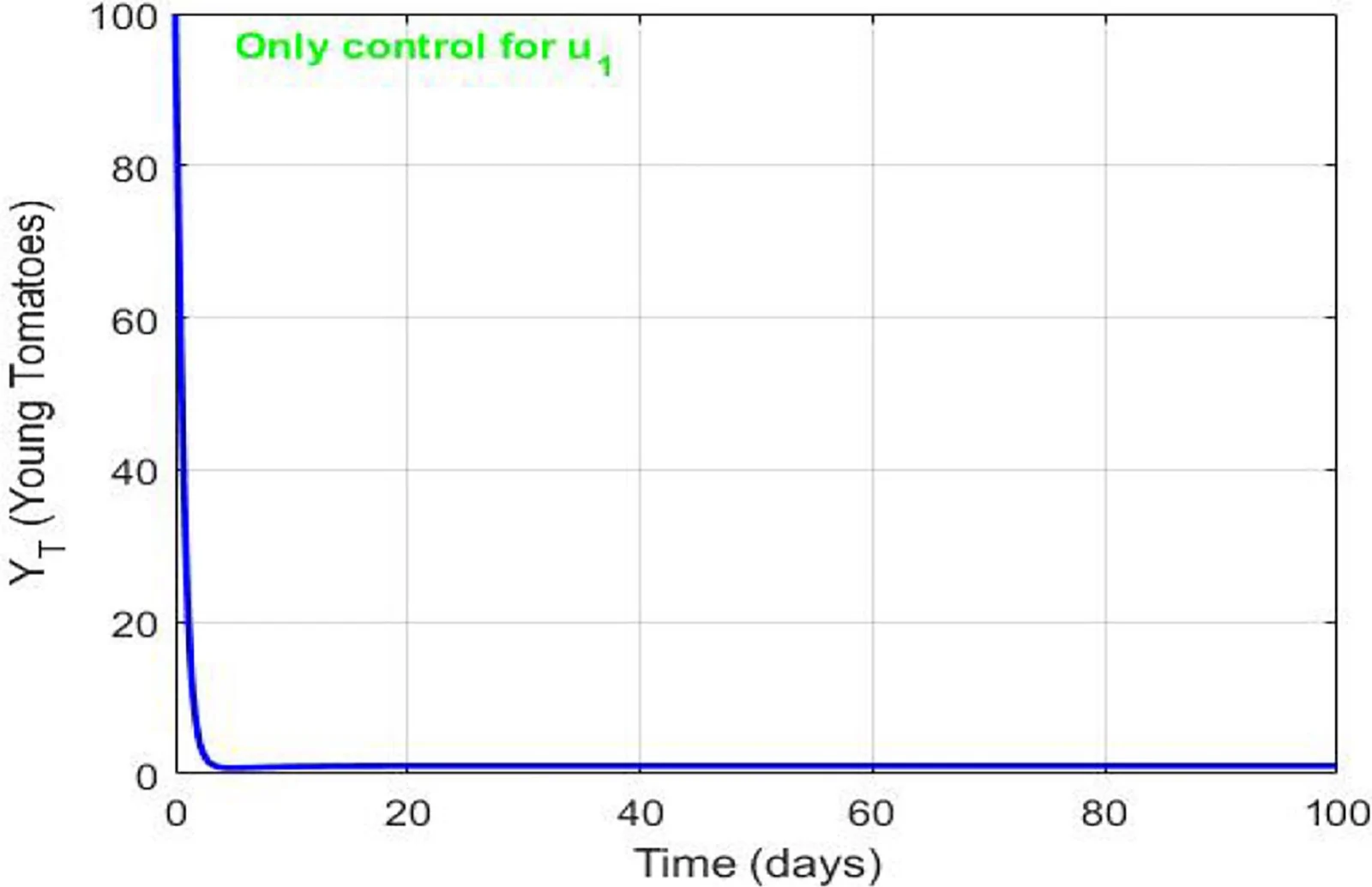
Dynamics of the young tomatoes $\left(Y_{T}\right)$ under under control $u_{2}$ over 100 days.

**Fig. 29:**
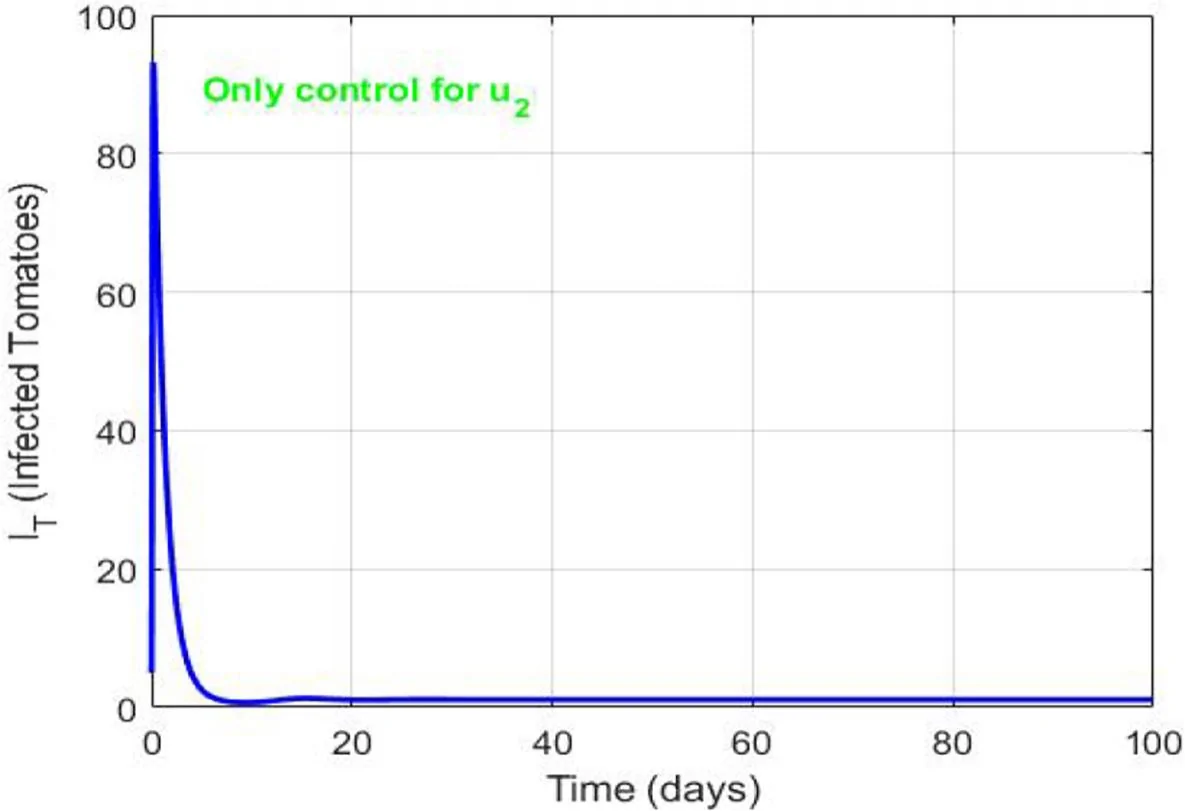
Dynamics of the infected tomatoes $\left(I_{T}\right)$ control $u_{1}$ over 100 days.

The findings indicate that the controls $u_{1}$ and $u_{2}$ effectively eliminate the disease by diminishing the population of susceptible and ill individuals. Fig. 30, Fig. 31 show that when $u_{3}$ is managed, transitional tomatoes $\left(T_{T}\right)$ decline from a peak of about 7–8 to nearly zero over 10–15 days. When under control, $u_{4}$, diseased tomatoes $\left(D_{T}\right)$ go from about 5 to 6 to less than 1, then stay at about 1.3. These results suggest that both controls reduce the number of infection stages, even though a few people remain infected under$\;u_{4}$. When mature tomatoes $\left(M_{T}\right)$ are placed under control $\left(u_{5}\right)$, they decrease from approximately 2–2.2 to about 0.2–0.3 over 10–15 days, as shown in Fig. 32, Fig. 33. In a controlled setting, the bacterial population $\left(B\right)$ drops from about 18 to 20 to nearly nothing in 5 to 10 days and stays very low. The results suggest that the steps taken to regulate the situation worked because they lowered the number of mature tomatoes and soil bacteria. Fig. 34, Fig. 35 show that infected tomatoes $\left(I_{T}\right)$ decline from about 65 to 100 to nearly nothing within 5 to 10 days when treated with the control. Without control, the infection status starts at a high level (about 100), then slowly declines and levels off after 30 to 40 days. These results suggest that control methods enable faster eradication of infections and disorders that persist in the system for long periods. Fig. 36, Fig. 37 show that transitional tomatoes $\left(T_{T}\right)$) drop from about fourteen to nearly nothing in ten to fifteen days when the control is used. When the control is not used, they remain around 1. With control, the number of diseased tomatoes $\left(D_{T}\right)$ goes down from 5 to 6 to 1. Without control, it can reach 3. The findings indicate that the control measures significantly diminish the infected stages and equilibrium levels. When there is a control, mature tomatoes$\left(M_{T}\right)$ drop from about 2.5 to 0.3–0.5, as illustrated in Fig. 38, Fig. 39. When there is no control, they stay consistent at around 0.8 to 1 (Fig. 40). When a control is utilized, the bacterial population $\left(B\right)$ drops from about 20 to nearly nothing in 10 to 15 days. When there is no control, it drops more slowly. These results suggest that control techniques lower maturity levels and accelerate the elimination of germs. Fig. 39 shows that the control variables u1 through $u_{6}$ start at high values ($u_{1}$ is about 100, and the others are between 10 and 30) and then drop swiftly to almost zero within 5 to 10 days. After that, all controls are not important. This means that a lot of early intervention is needed to slow the disease's progression, but only a little control is needed to keep things stable and prevent people from getting sick again. Fig. 41 shows how different control strategies for the infected tomato population change over a 100-day experiment. The solid blue curve shows the optimal way to control the situation, showing the path of contaminated tomatoes, while the dashed red curve shows what happens when no action is taken. On the other hand, when control measures are put in place, the levels of infection drop quickly and sharply, with $\left(I_{T}\right)$ going down to almost zero in about 15 days. This shows that the best way to control the disease is highly effective at stopping its spread, which lowers the peak infection rate. As these results show, timely, targeted treatments are very important for controlling tomato disease outbreaks. Fig. 42 compares the growth of the tomato population $\left(T_{T}\right)$ over 100 days in both controlled and uncontrolled settings. These tomatoes have transferred from the diseased compartment to the recovered or removed compartment. In a situation with no controls, the transitory population gets to roughly 12.5 units on day 2 and then slowly drops to practically zero after 40 days. Using the best controls shifts the peak earlier and the drop much faster, but it only reaches approximately 13.8 units. The managed transitional population had fallen to nearly nothing by day 15, well before the uncontrolled situation. As shown in Fig. 43, in the absence of control, the diseased tomato population $\left(D_{T}\right)$ increases quickly to around 6 plants by day 1, decreases to about 1.7 plants by day 8, and stays constant at around 2.8 plants for the whole 100 days. According to the best controls, the early infection pressure stays steady since the initial peak stays about 6. However, the post-peak dip falls below 0.6 after day 8, and$\left(D_{T}\right)$ remains steady at approximately 1.0 after day 20. To rephrase, the control strategy lowers the equilibrium level and temporarily suppresses more effectively, resulting in a 64% reduction in the long-term burden of tomato contamination. Fig. 44 demonstrates how Mature tomatoes $\left(M_{T}\right)$ changed over 100 days in two different situations: one with control (blue line) and one without (red dashed line). $\left(M_{T}\right)$ reaches its highest point, about 4.5, on day 5 without control. After that, it stays around 1.0. They both start at the same level. When there is control, the peak is lower at 2.8 and quickly returns to about 0.5. The fact that control measures can lower the peak and maintain a lower, more consistent level of mature tomatoes shows that disease or growth management is improving. Fig. 45 shows that the soil bacterial population $\left(B\right)$ changed in two ways after one hundred days: with a control (blue solid line) and without (red dashed line). At first, they both start at a high level, close to 20. If nothing is done, the number of bacteria will drop to practically zero by day forty. Control, on the other hand, drops much more quickly, going below 5 by day 10 and very near to zero by day 20. This shows that control methods accelerate the death of harmful soil bacteria, thereby making the intervention more effective at maintaining soil health. The six distinct combined control strategies varied tomato levels over time, as shown in Fig. 45. At the start of the game (day 0), every strategy starts with a high tomato level. The highest starting value is $u_{1}$, which is close to 100. The efficiency of any plan declines a lot in the first ten to fifteen days. It is important to note that $u_{1},u_{2}$, and $u_{4}$ rapidly reduce tomato levels to nearly zero within 10 days. This suggests that they work very well right away. At first, controls $u_{5}$ and $u_{6}$ don't do much, but over time, they keep tomato levels stable. By day 30, all the ways to manage the tomato levels work together to keep them virtually at zero (Fig. 46).

**Fig. 30:**
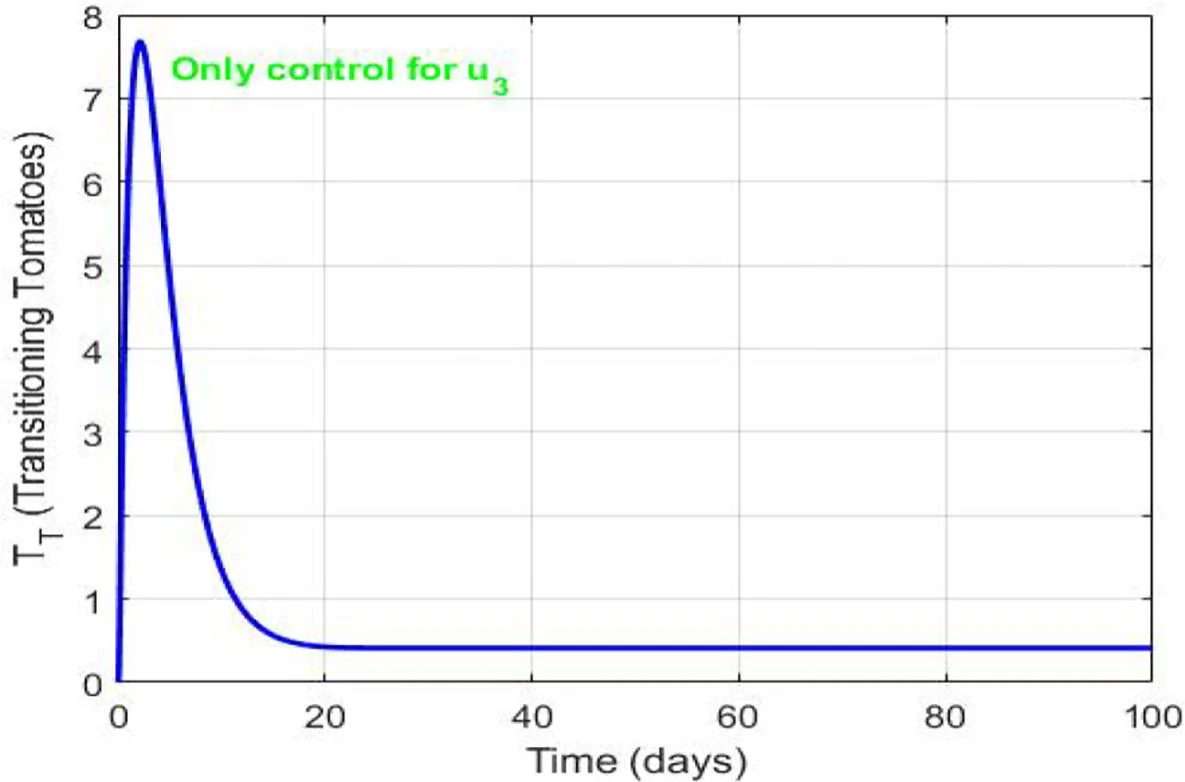
Dynamics of the transitioning tomatoes $\left(T_{T}\right)$ under control $u_{3}$ over 100 days.

**Fig. 31:**
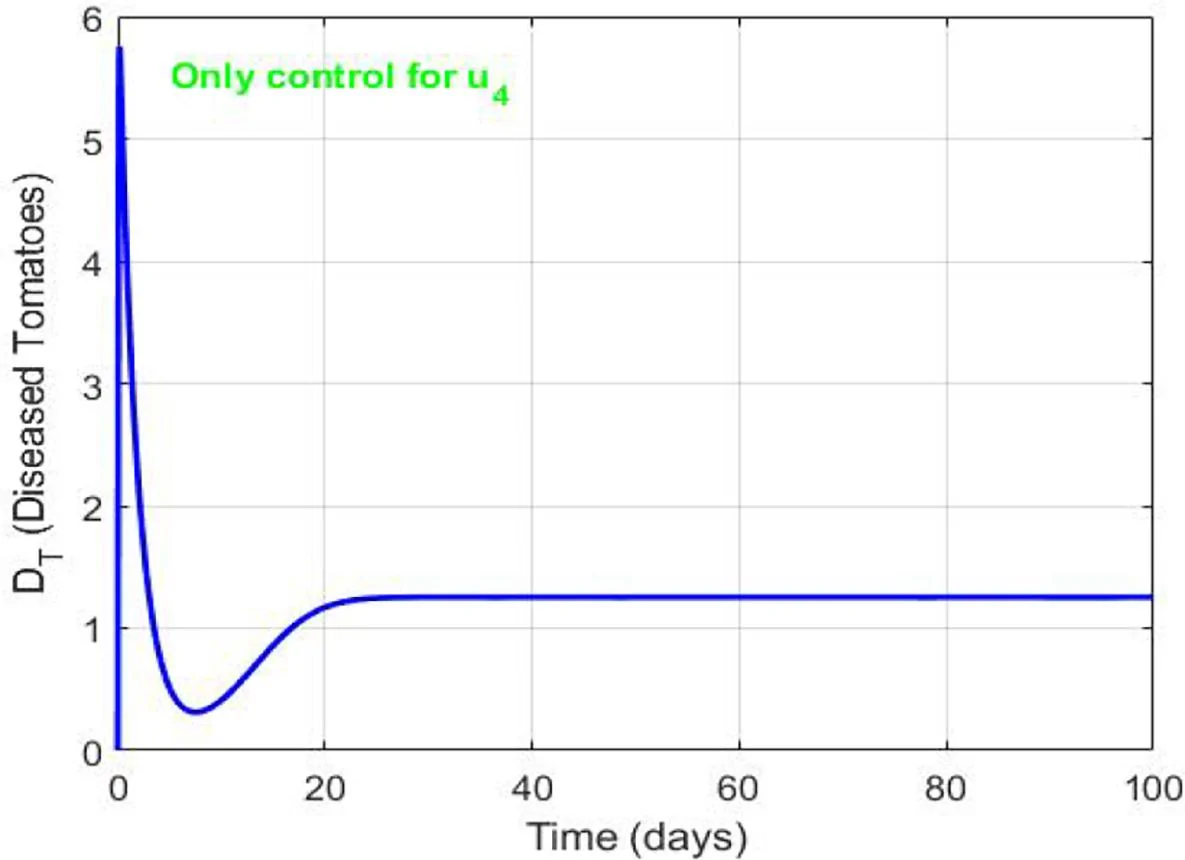
Dynamics of the diseased tomatoes $\left(D_{T}\right)$ under control $u_{4}$ over 100 days.

**Fig. 32:**
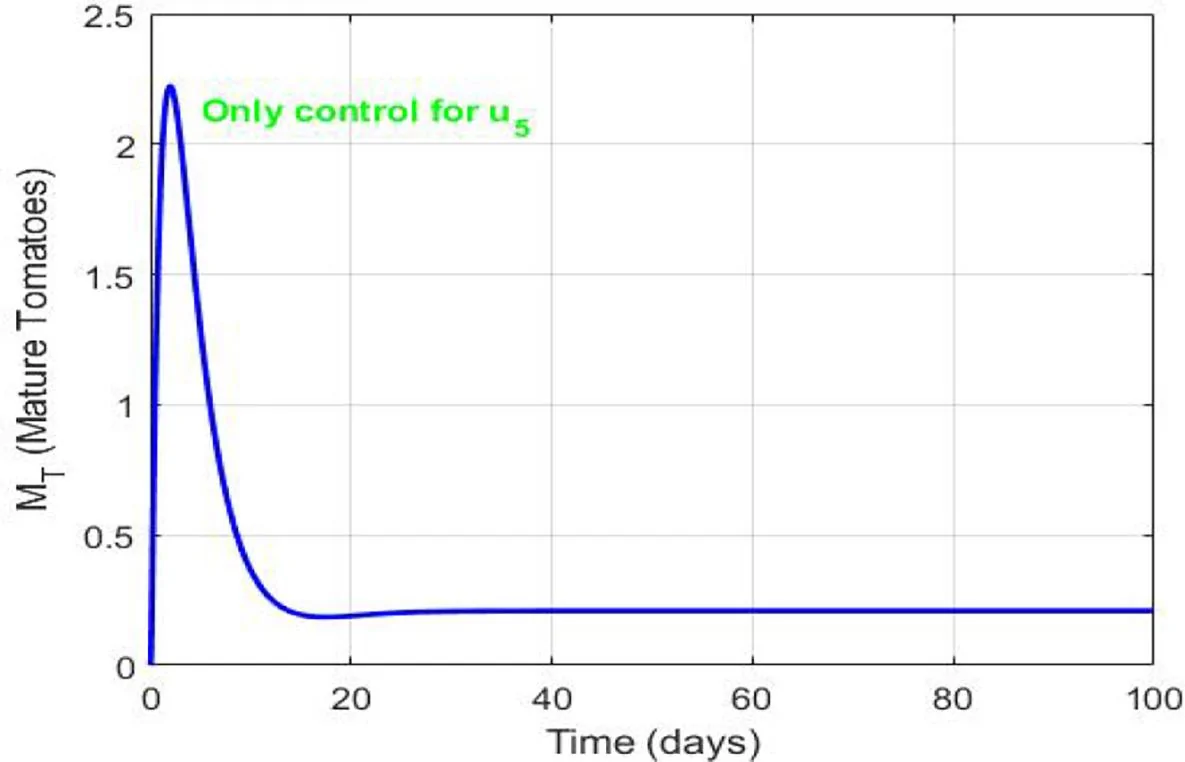
Dynamics of the mature tomatoes $\left(M_{T}\right)$ under control $u_{5}$ over 100 days.

**Fig. 33:**
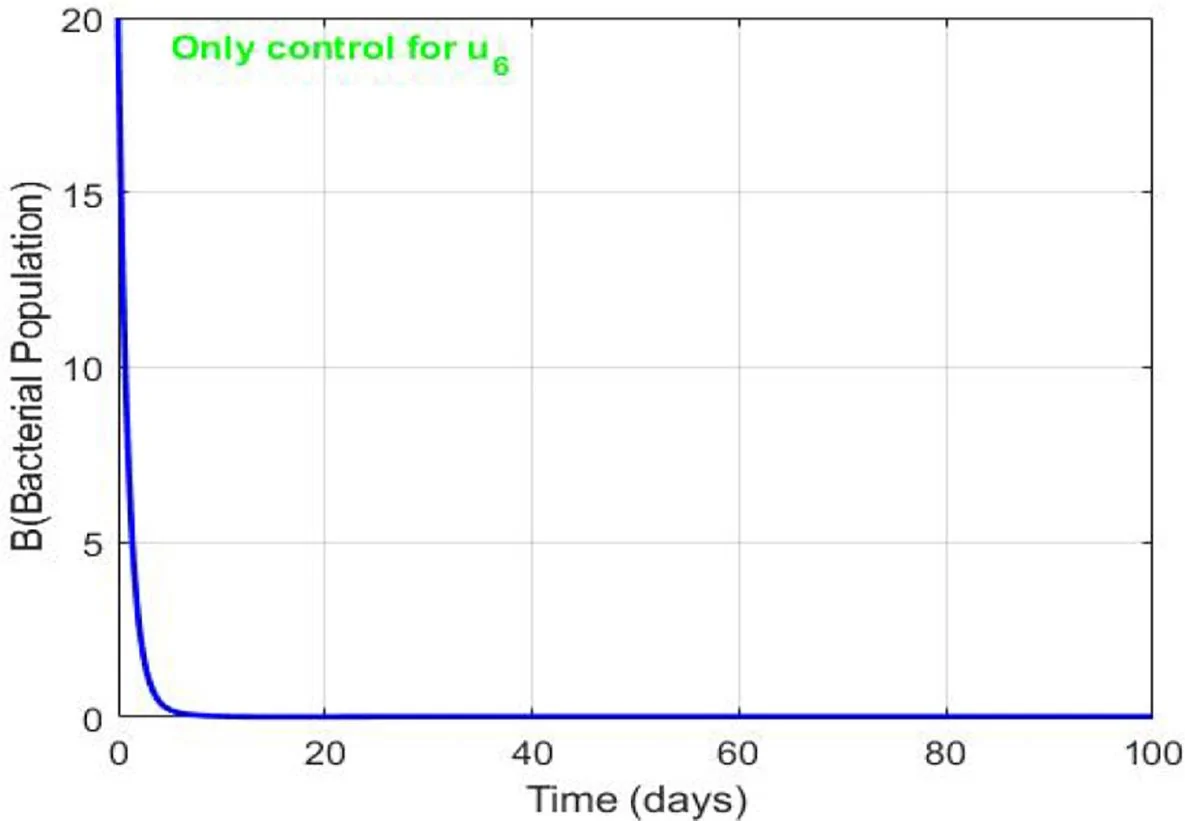
Dynamics of the bacterial population $\left(B\right)$ under control $u_{6}$ over 100 days.

**Fig. 34:**
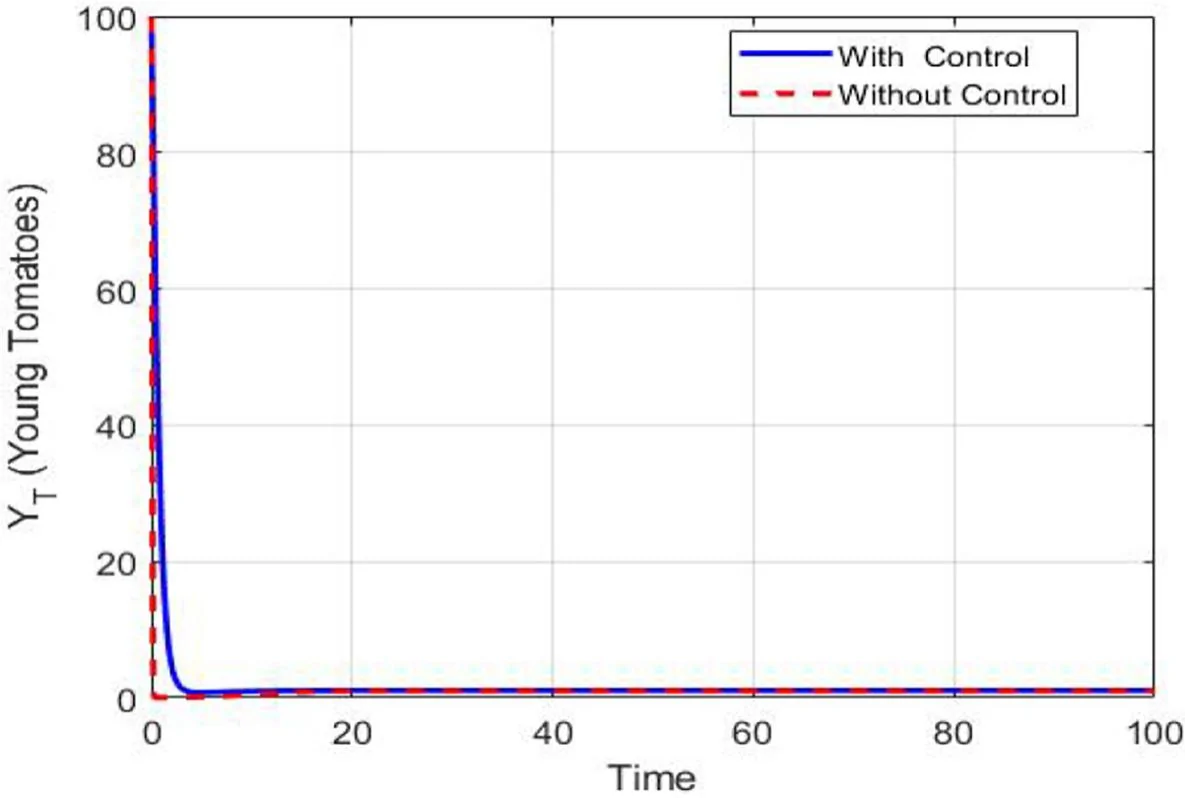
Dynamics of infected tomatoes$\left(Y_{T}\right)$ and without control.

**Fig. 35:**
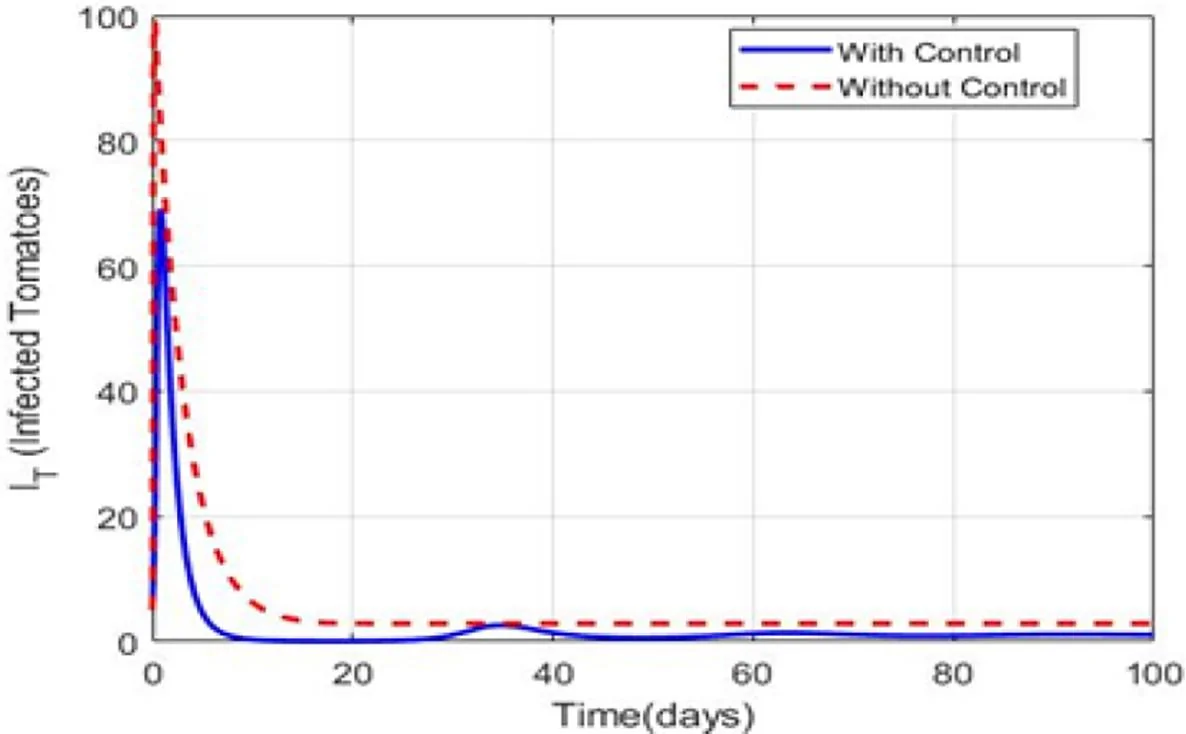
Dynamics of infected tomatoes$\left(I_{T}\right)$ over time with over time with and without control.

**Fig. 36:**
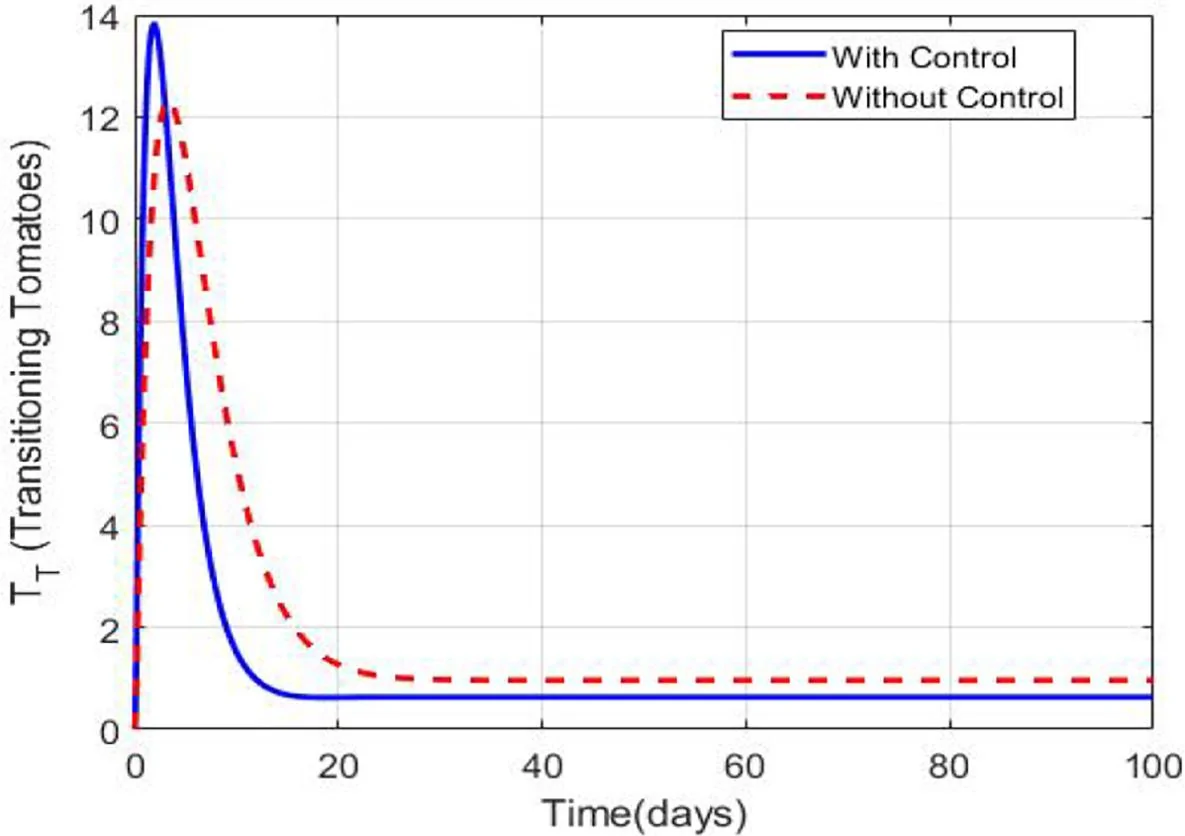
Dynamics of transitioning tomatoes$\left(T_{T}\right)$ and without control.

**Fig. 37:**
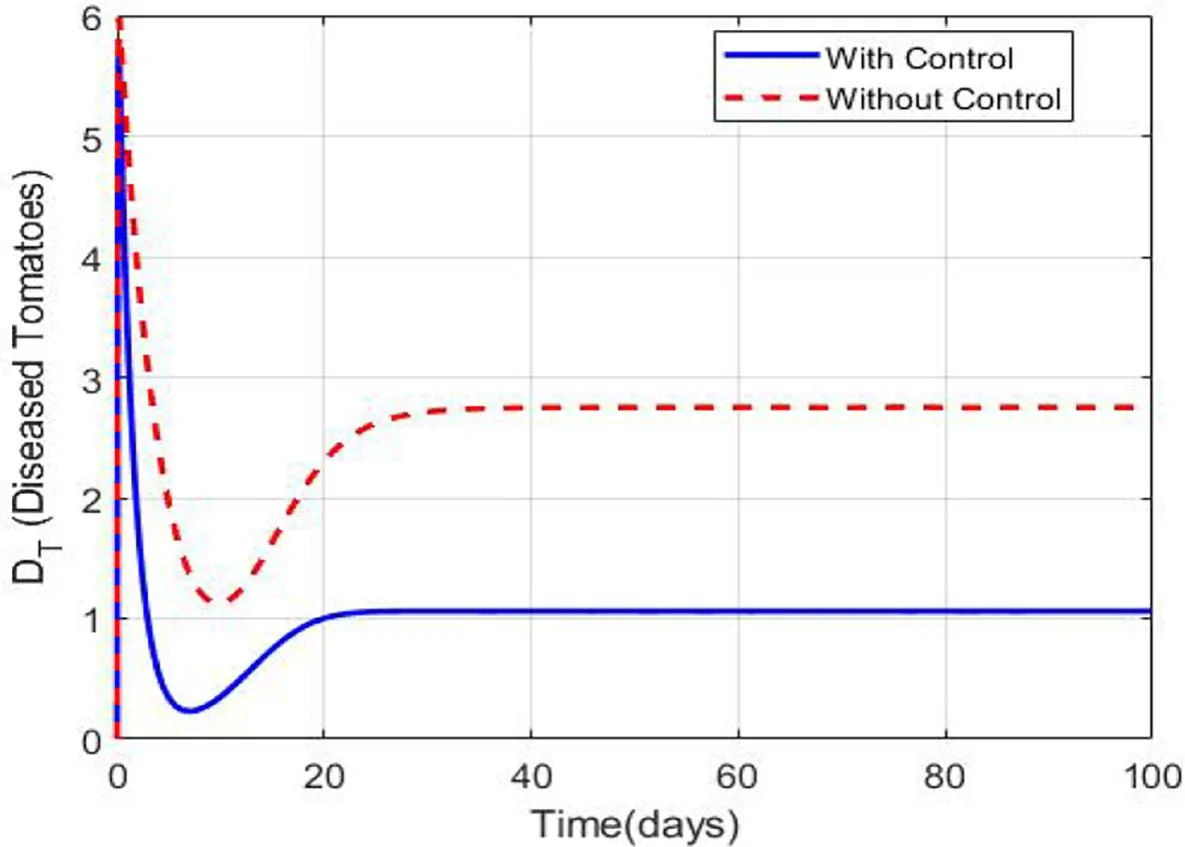
Dynamics of diseased tomatoes$\left(D_{T}\right)$ over time with over time with and without control.

**Fig. 38:**
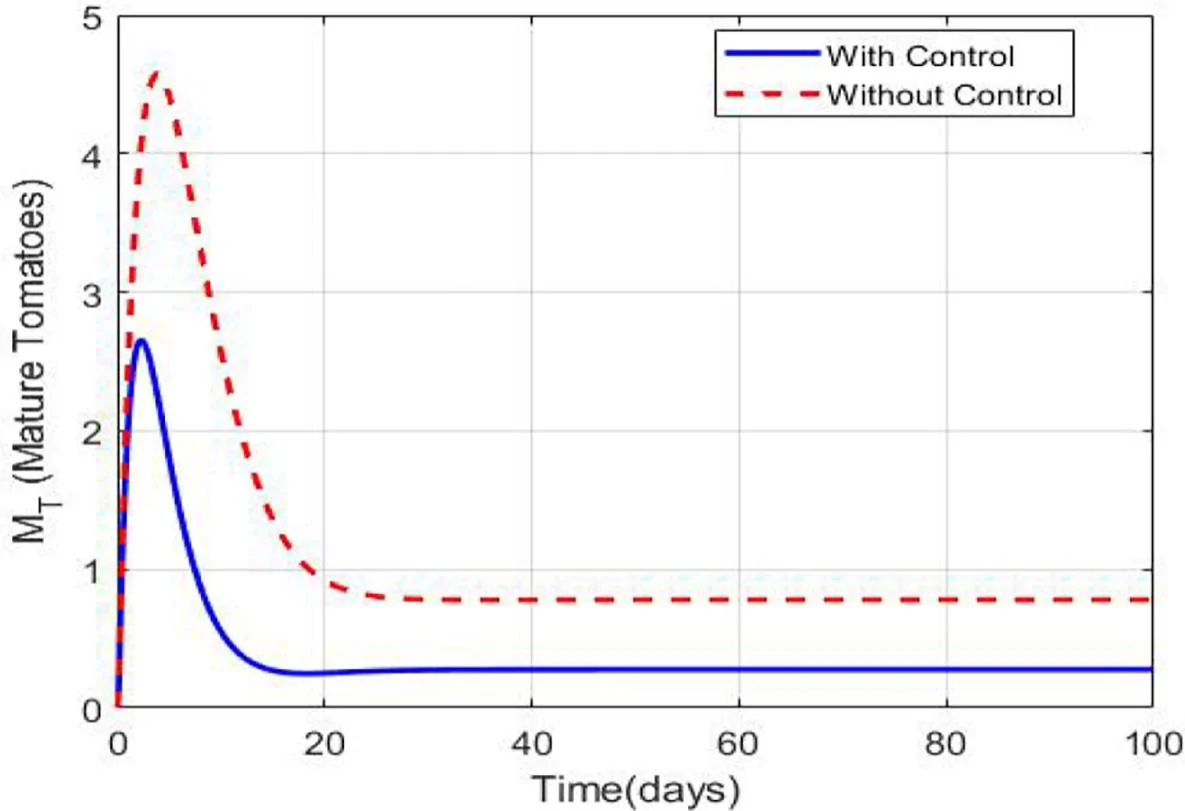
Dynamics of mature tomatoes$\left(M_{T}\right)$ over time over time with and without control.

**Fig. 39:**
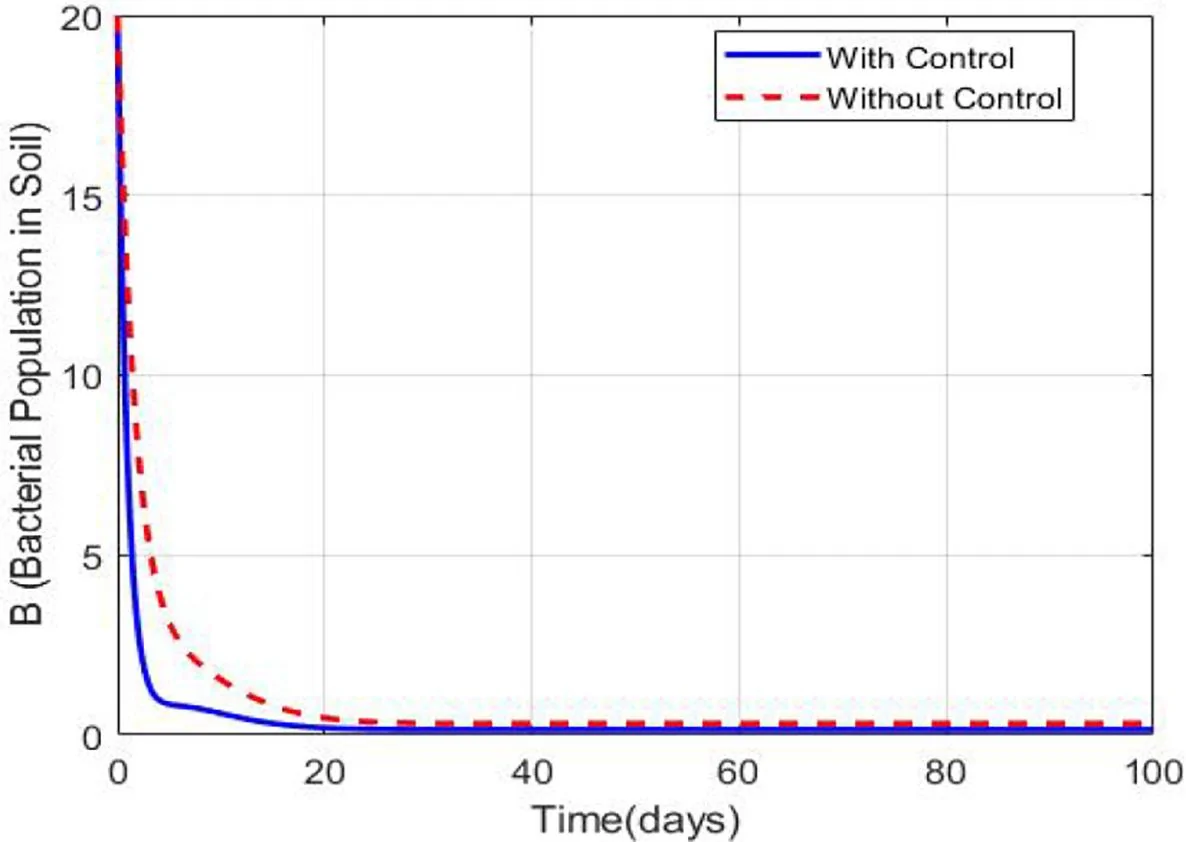
Dynamics of bacterial population in soil$\left(B\right)$ with and without control.

**Fig. 40:**
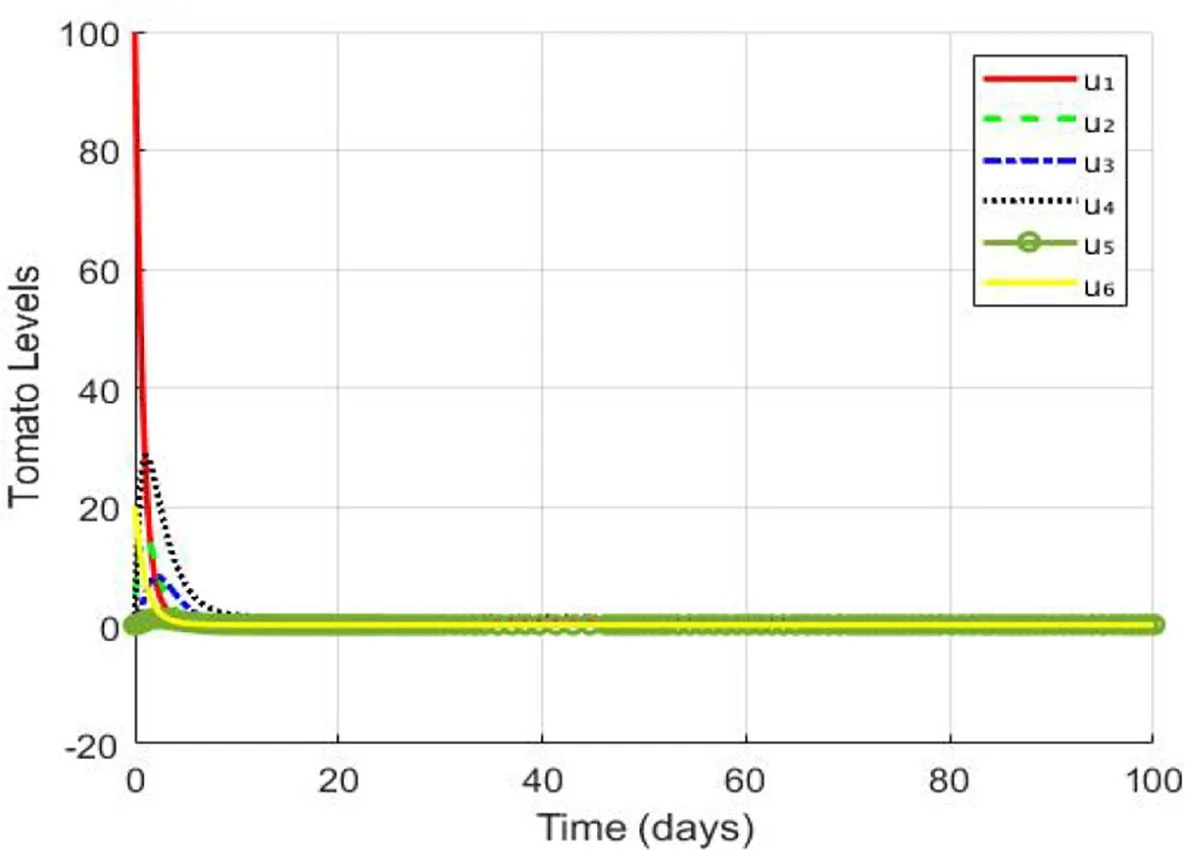
Dynamic impact of individual optimal controls $u_{1}$ to $u_{6}$ levels over time.

**Fig. 41:**
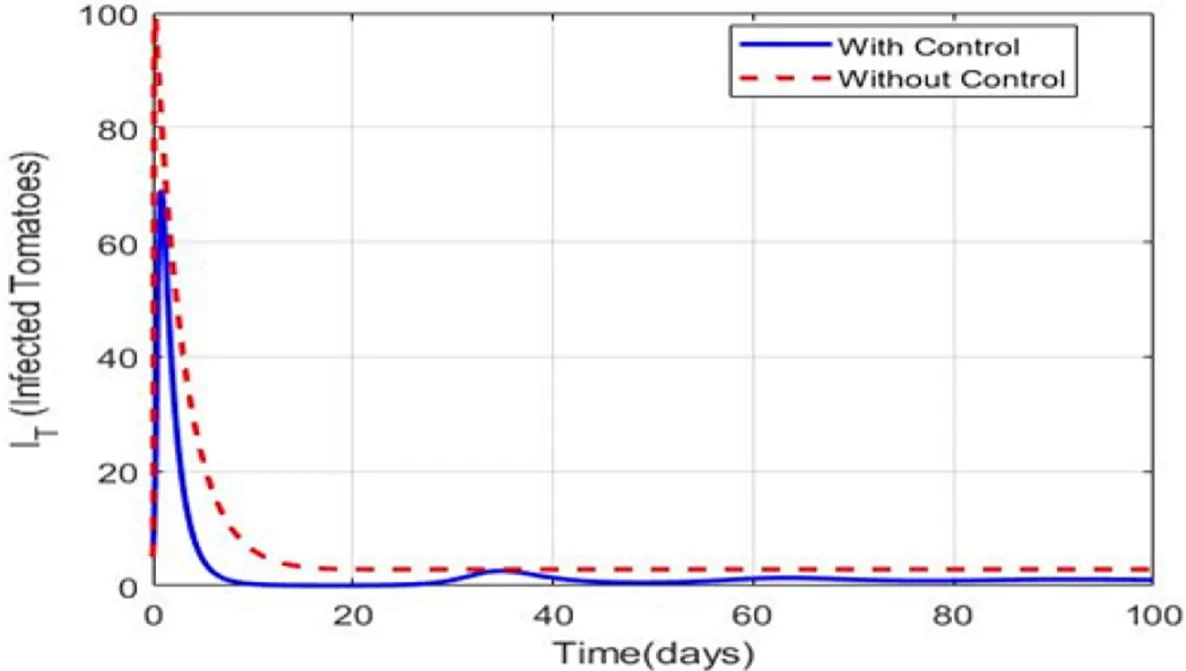
Dynamics of infected tomatoes$\left(I_{T}\right)$ over time with and without control.control.

**Fig. 42:**
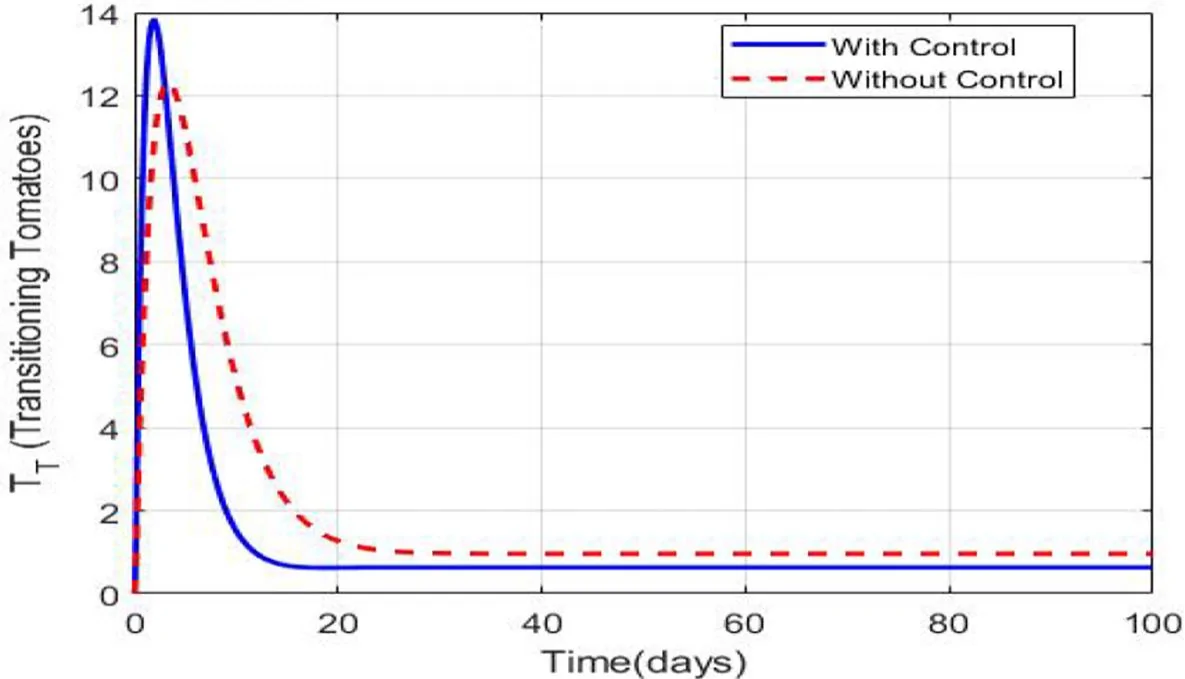
Dynamics of transitioning tomatoes$\left(T_{T}\right)$ over time with and without control.

**Fig. 43:**
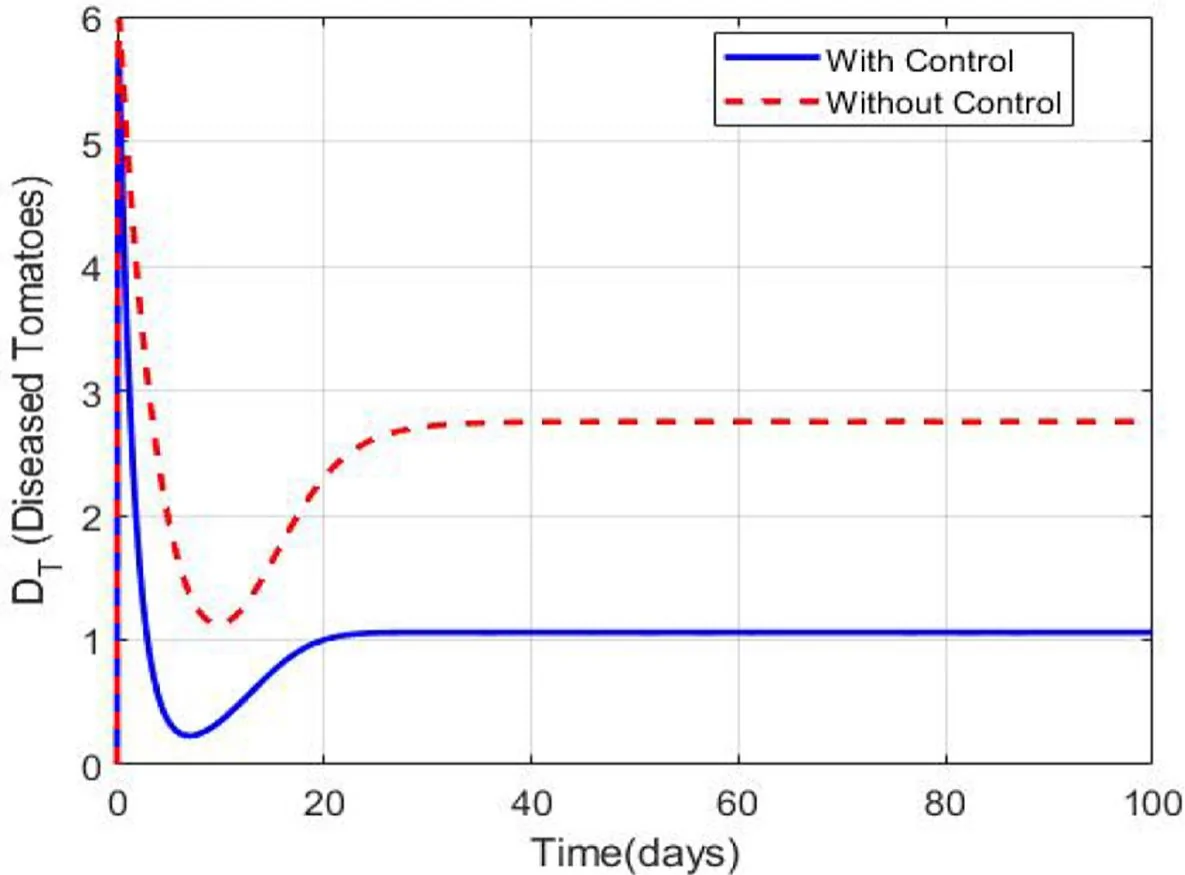
Dynamics of diseased tomatoes$\left(D_{T}\right)$ over time with and without control.

**Fig. 44:**
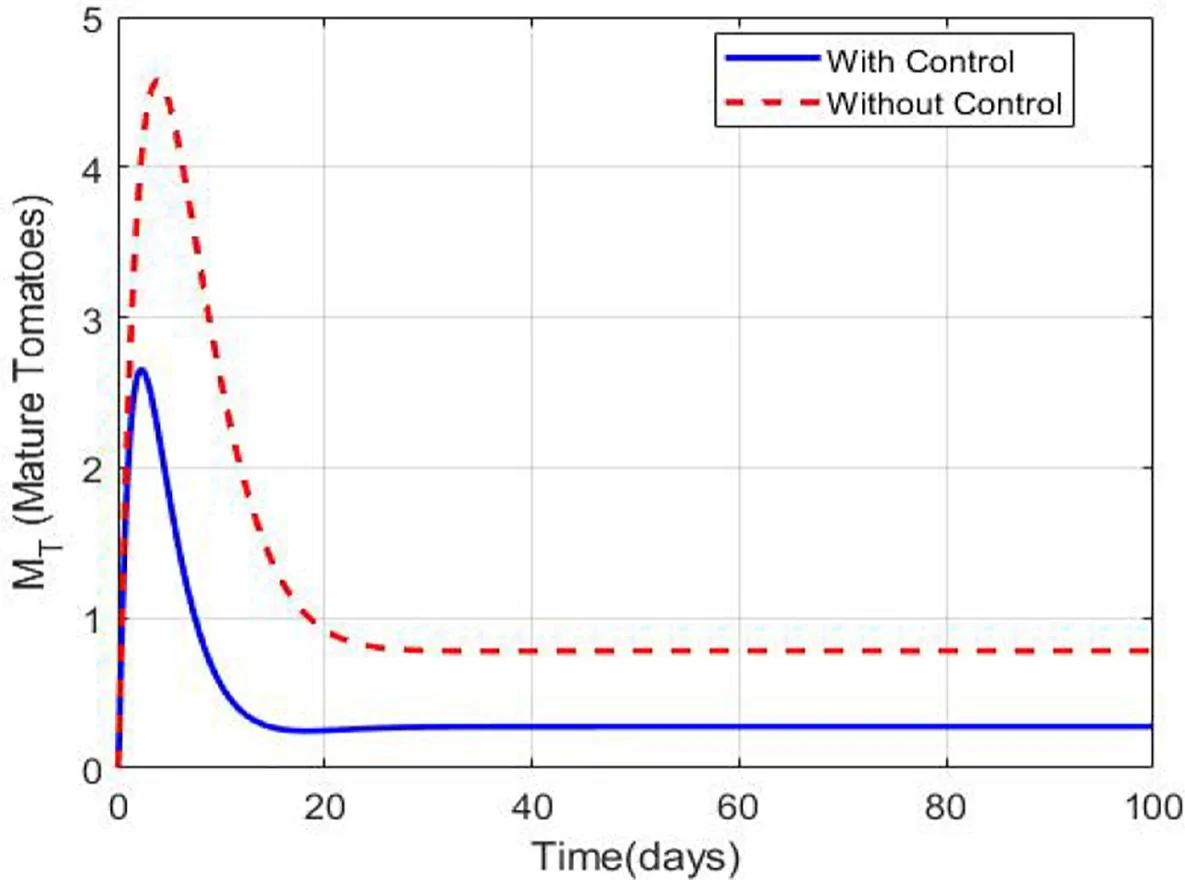
Dynamics of Mature tomatoes$\left(M_{T}\right)$ over time with and without control.

**Fig. 45:**
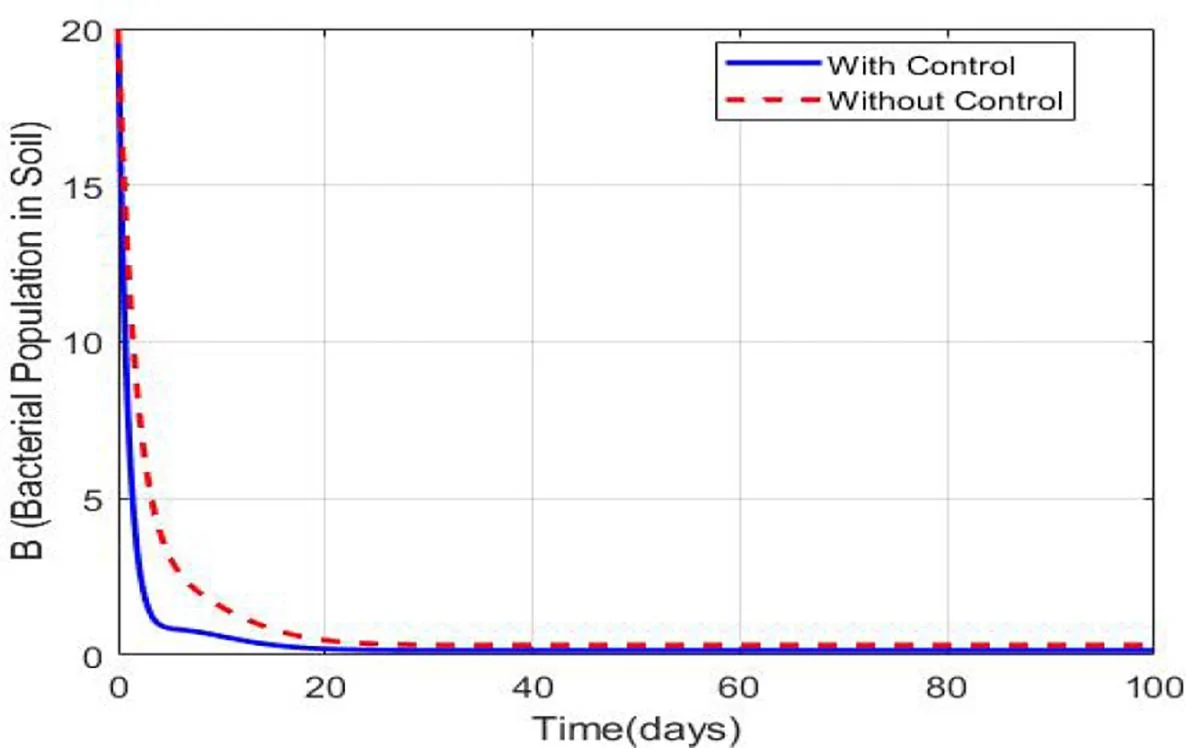
Dynamics of Bacterial Population in Soil$\left(B\right)$ over time with and without control.

**Fig. 46:**
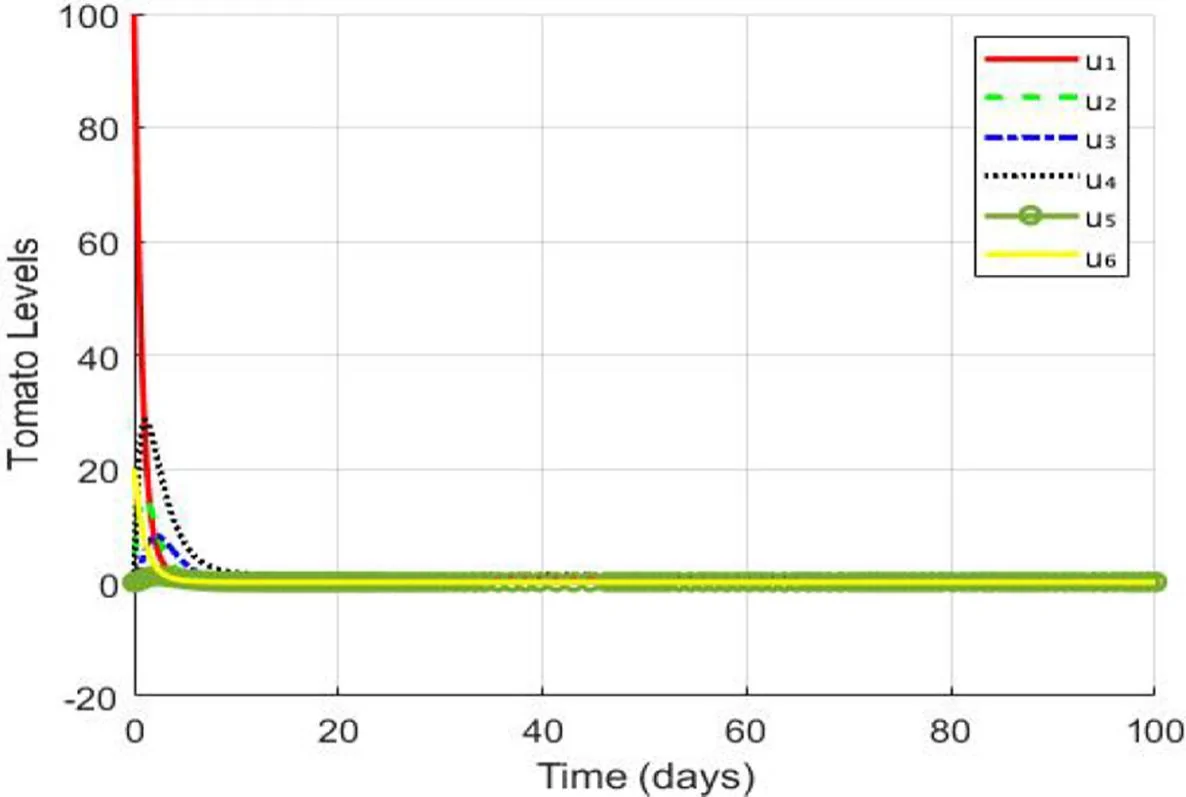
Dynamic impact of individual optimal controls $u_{1}$ to $u_{6}$ on tomato levels over time.

## 11 Concluding remarks

In this work, a mathematical model was developed to explore the transmission of bacterial wilt in tomatoes by taking into account the plant growth stages, disease progression, and soil bacterial dynamics. The numerical and bifurcation analyzes show that the changes of important epidemiological parameters, especially the transmission rate ρ can have a large impact on stability of the system and persistence of diseases. The analytical results show the importance of the basic reproduction number $R_{0}$ as a threshold quantity for disease dynamics. Analysis of the equilibrium of the infected tomato population reveals complex illness dynamics involving bistable, unstable, and stable stages. But it turns out that if one want to get rid of diseases, just maintaining the basic reproduction number below one isn't necessarily enough, because reverse bifurcation occurs. Therefore, to effectively manage tomato bacterial wilt, one must intervene firmly and persistently to restore the system to a disease-free equilibrium condition. The analysis shows backward-bifurcation dynamics, i.e. the existence of an unstable branch besides stable disease-free and endemic equilibria. This bistability implies that eradication of disease may not be attainable if the reproduction number is less than one.

The most relevant parameter to affect $R_{0}$ in sensitivity analysis was determined to be ρ, hence minimization of effective transmission should be a key objective in disease-management techniques. The relative contribution of bacteria and natural decay in pathogen persistence in soil might be very important for the long term outcome however the correct transition and removal processes can reduce infection related compartments as shown by the simulations. The behavioral research also suggests that control measures may not be applied because of high intervention costs, highlighting the need for economically viable management strategies. Optimal-control results show that early coordinated therapies can reduce disease faster and bring the system to a low disease state with less intense maintenance. These interventions can target sensitive plants, diseased plants and soil microbes. The results suggest that reducing transmission, rapidly treating or removing infected plants, and properly managing soil pathogens are essential for the long-term effective management of tomato bacterial wilt. Further work may introduce data-driven estimates of parameters, machine-learning techniques, fractional-order dynamics, economic optimization, spatial and stochastic modeling, etc., into the present framework to improve its biological realism and practical utility.

## CRediT authorship contribution statement

**Md. Asraful Islam:** Writing – review & editing, Validation, Supervision, Resources, Methodology, Investigation, Formal analysis, Conceptualization. **Mymuna Akther:** Writing – original draft, Visualization, Software, Resources.

## Funding statement

This research did not receive any specific grant from funding agencies in the public, commercial, or not-for-profit sectors.

## **Declaration of competing interest**

The authors declare that they have no known competing financial interests or personal relationships that could have appeared to influence the work reported in this paper.

## Data Availability

All data used in this work can be found in the work
